# Human and dog Bayesian dietary mixing models using bone collagen stable isotope ratios from ancestral Iroquoian sites in southern Ontario

**DOI:** 10.1038/s41598-023-34216-6

**Published:** 2023-05-03

**Authors:** John P. Hart

**Affiliations:** Research and Collections Division, New York State Museum, Albany, NY USA

**Keywords:** Ecological modelling, Ecology

## Abstract

Under the archaeological canine surrogacy approach (CSA) it is assumed that because dogs were reliant on humans for food, they had similar diets to the people with whom they lived. As a result, the stable isotope ratios of their tissues (bone collagen and apatite, tooth enamel and dentine collagen) will be close to those of the humans with whom they cohabited. Therefore, in the absence of human tissue, dog tissue isotopes can be used to help reconstruct past human diets. Here *δ*^13^C and *δ*^15^N ratios on previously published dog and human bone collagen from fourteenth-seventeenth century AD ancestral Iroquoian village archaeological sites and ossuaries in southern Ontario are used with MixSIAR, a Bayesian dietary mixing model, to determine if the dog stable isotope ratios are good proxies for human isotope ratios in dietary modeling for this context. The modeling results indicate that human dietary protein came primarily from maize and high trophic level fish and dogs from maize, terrestrial animals, low trophic level fish, and human feces. While isotopes from dog tissues can be used as general analogs for human tissue isotopes under CSA, greater insights into dog diets can be achieved with Bayesian dietary mixing models.

## Introduction

Multiple lines of archaeological evidence are used to reconstruct the diets of past people. These can include macrobotanical, microbotanical, and animal bone assemblages; lipids and proteins extracted from pottery fabric and charred encrusted cooking residues; stable isotope analyses of cooking residues, specific compounds extracted from residues and pottery fabrics, and human tissue (generally bone collagen and apatite and/or tooth dentine collagen and enamel). The last of these can be among the most important because human tissues provide direct evidence of resource consumption: “you are what you eat”^1^. However, in many regions this is not possible because of the lack of suitable remains, objections from descendant communities to destructive analyses of human tissue, and laws that require the repatriation of human remains to descendant communities. One way that archaeologists have responded to the lack of human remains is to analyze dog tissue. This is done under the assumption that dogs relied on humans for much their food and, therefore, stable isotopic ratios of dog tissue may be close to the tissues of human with whom they lived, depending on environmental, bio-behavioral, and cultural factors (^2^, p. 362). This has been labeled the canine surrogacy approach (CSA;^2^) and has been applied in several studies in northeastern North America (e.g.,^3–6^) as well as other regions of the continent (e.g.,^7,8^). These studies have either used dogs as direct analogs for human diets or as indirect analogs to assess the extent to which maize, a C_4_ plant in an otherwise C_3_ plant biome, was a significant food resource^2^.

Recently, Glencross and colleagues^9^ used new *δ*^13^C and *δ*^15^N ratios on large series of dog bone collagen from Iroquoian village sites in southern Ontario to test their applicability as direct analogs for reconstruction of fourteenth–seventeenth century AD human diets by comparing them to previously published ratios on human bone collagen from the same village site or associated ossuary. They conclude that *δ*^13^C ratios are good proxies for human ratios. While dog *δ*^15^N ratios are < 2‰ lower than human ratios, they “generally support the premise that dogs can serve as dietary proxies for humans, any approximations would likely underestimate the actual human values” (^9^, p. 12). However, these results do not directly address the issue of whether dog and human diets were similar enough that stable isotope ratios of dog bone are suitable direct analogs for humans. While the values are similar, dogs are not people and have different consumption patterns (e.g., caecotrophy), the possibilities of which Glencross and colleagues (^9^, pp. 7, 12) mention and is recognized in the broader CSA literature^2^ While this analysis resulted in important insights into human and dog diets in the region, it is possible to move beyond the typical applications of CSA by using Bayesian dietary mixing models to gain more detailed dietary estimates for dogs as isotopic analogs for associated humans.

Used extensively in other disciplines (e.g.,^10–13^) and in archaeological human and dog dietary studies globally (e.g.,^8,14–18^) but rarely in northeastern North American archaeology^19–21^, Bayesian dietary mixing models can be used with multiple tracers (e.g., stable isotope ratios) of consumers and potential resources (sources), taking into account trophic enrichment factors (TEF), and other sources of prior information, to estimate dietary fractions of specific resources and resource groups. Here I use MixSIAR^22^, which uses a Markov Chain Monte Carlo simulation to produce models of probable food source fractions in consumers’ diets. Included in the simulations are isotopic uncertainties of sources, consumers, and TEFs. This results in probabilities of resource fractions in consumer diets, allowing for more informed assessments of diets than those based on single isotopes or two isotope scatterplots as are typically used in CSA. I compare modeled protein sources in the diets of humans and dogs by century and specific village sites and, where appropriate, associated ossuaries. In the models I use large datasets of source and human and dog isotopic ratios on bone collagen extracted from the literature; collagen isotopes reflect primarily the protein fraction of diets^23^.

## Contemporary free-ranging dog diets

Globally, most contemporary dogs are not household pets^24^. Rather, they are associated with households but range freely within and beyond settlements, relying primarily on human-sourced food for sustenance. This includes direct feeding by humans and opportunistic scavenging from garbage dumps and other human-derived waste, and sheltering in human structures (e.g.,^24^, p. 147). Based on a series of experiments with free-ranging dogs in India, Sarkar and colleagues (^25^, p. 43) found that foraging free-ranging dogs preferred protein-rich over carbohydrate-rich foods and followed a “rule of thumb” that “if it smells like meat, eat it.” Butler and colleagues^26^ report on the diets of free-ranging dogs in remote agricultural communities of northwestern Zimbabwe. Dogs are associated with specific households but are unrestrained and roam widely. The primary component of human diets in the region is *sadza* a porridge composed primarily of maize (*Zea mays* ssp. *mays*) but which may include other grains such as millet (*Pennisetum americanum*), sorghum (*Sorghum bicolor*), or finger millet (*Eleusine coracana*), and is also fed to dogs. Uneaten food and other waste are disposed of in open pits external to houses, which are accessible to dogs. Most households lack plumbing or latrines, and people defecate in the open away from their houses, shallowly burying their feces, and so are accessible by dogs. Resource frequency occurrence in 945 analyzed dog scats (^26^, p. 6) were: *sadza* (87.9%), mammal remains (81.3%), vegetables and fruit (69.8%), and human feces (56.2%). Observation of 689 meals recorded consumption of animal remains in 48.8%, *sadza* in 22.1%, and human feces in 20.5% (^26^, p. 8). In total, 13% of dog diets were directly fed by humans, while the remainder was obtained by foraging resources ultimately derived from human activities. Similar results have been found for free-ranging dogs in other regions (e.g.,^27,28^), with a primary dietary emphasis on animal carcasses, human feces, and grains.

## Evidence for Iroquoian dog and human diets

Very little evidence for Iroquoian dog behavior and consumption patterns is provided in the 17^th^-century southern Ontario ethnohistorical record. Relevant to current purposes is a brief account by Sagard (^29^, p. 226) suggestive of contemporary free-ranging dog dietary behavior: “their most usual fare is nothing but the refuse they find in the streets and on the roads. They also very frequently put their pointed nose into the savages’ pot of *sagamité.*” *Sagamité*, like *sadza* in Zimbabwe, was a maize-based stew or porridge into which was added fish or terrestrial animal tissue and/or other crops such as common bean (*Phaseolus vulgaris*), squash (*Cucurbita pepo*), and sunflower (*Helianthus annuus*)^30,31^.

Maize is generally the most ubiquitous and abundant food-related macrobotanical remain recovered from ancestral Iroquoian archaeological sites (e.g.^32^). Isotopic analysis of fourteenth-seventeenth century ancestral Iroquoian bone and teeth indicate the importance of maize in diets; maize accounting for > 50% to > 70% of consumed resources (e.g.^20,33^). Freshwater fish remains are common on ancestral Iroquoian archaeological sites in southern Ontario (e.g.^34^), and isotopic analyses of human tissue confirms the importance of fish in human diets^20,33,35^. While mammal and bird bone are common on ancestral Iroquoian sites in southern Ontario (e.g.,^36,37^), isotopic analyses suggest terrestrial animal resources did not contribute substantially to human diets^20^.

## Results

The CSA literature indicates dog collagen *δ*^15^N ratios are typically 2–3‰ lower than human ratios, although the differences between the two species vary widely (^2^, pp. 354–356); Glencross and colleagues (^9^, p. 12) indicate a mean difference of < 2.0‰ in *δ*^15^N ratios between Iroquoian dogs and humans. Nonparametric Mann–Whitney tests of dog and human *δ*^15^N ratios by century have significant differences in sample medians and the nonparametric Epps-Singleton test indicates the sample distributions are significantly different (Table 1). This suggests that the isotope ratios potentially reflect different diets. While caceotrophy has been suggested as a possible cause of the lower *δ*^15^N ratios in dogs^2^, it is also possible based on what is known about free-range dog dietary behavior that differences may result from consumption of different resources than those comprising human diets or the same resources but in different proportions. The MixSIAR results indicate that both are likely causes. Results of the models are presented in Tables 2, 3, Figs. 2, 3, 4, 5, and Supplementary Data S1 Tables 1, 2, 3, 4, 5, 6, online. Model diagnostics are also provided in Supplementary Data S1, online.

**Table 1:** Results of non-parametric two-sample tests for human and dog δ^15^N and δ^13^C ratios by century.

Century (AD)	Human	Dog	Mann–Whitney			Epp-Singleton test	
	n	n	*U*	*z*	*p*^a^	*W*_2_	*p*
14^th^	23	13	3.5	4.7994	0.00001	95.42	0.00000
15^th^	22	19	7.0	5.2731	0.00001	100.91	0.00000
16^th^	20	21	5.5	5.3279	0.00001	125.09	0.00000
17^th^	16	41	9.0	5.6616	0.00001	254.05	0.00000

^a^Monte Carlo permutation *p*-values.

**Table 2:** Dog and human MixSIAR model 2.5% (50.0%) 97.5% quantiles by century.

Source	Human	Dog Model 1	Dog Model 2	Dog Model 3
Fourteenth century human (n = 23), dog (n = 13)				
Maize	0.417 (0.474) 0.526	0.409 (0.498) 0.576	0.312 (0.451) 0.547c	0.300 (0.443) 0.546
Terrestrial	0.002 (0.050) 0.150	0.041 (0.285) 0.429b	0.040 (0.269) 0.411d	0.052 (0.283) 0.425e
High δ^15^N Fish	0.055 (0.181) 0.343a	0.001 (0.028) 0.106	0.001 (0.023) 0.093	0.000 (0.018) 0.084
Med. δ^15^N Fish	0.008 (0.132) 0.406a	0.002 (0.045) 0.162	0.002 (0.035) 0.148	0.001 (0.030) 0.136
Low δ^15^N Fish	0.005 (0.077) 0.231	0.004 (0.111) 0.421b	0.005 (0.092) 0.388d	0.002 (0.080) 0.378e
Human Feces			0.003 (0.080) 0.326c	0.003 (0.080) 0.326
Micromammals				0.001 (0.021) 0.098
Fifteenth century human (n = 22), dog (n = 19)				
Maize	0.460 (0.508) 0.552	0.509 (0.562) 0.608	0.430 (0.530) 0.589c	0.429 (0.528) 0.591
Terrestrial	0.002 (0.049) 0.159	0.158 (0.328) 0.406b	0.136 (0.310) 0.391d	0.134 (0.315) 0.396e
High δ^15^N Fish	0.026 (0.200) 0.339a	0.000 (0.015) 0.060	0.000 (0.012) 0.050	0.000 (0.010) 0.043
Med. δ^15^N Fish	0.007 (0.133) 0.387a	0.001 (0.022) 0.092	0.001 (0.019) 0.084	0.000 (0.017) 0.074
Low δ^15^N Fish	0.004 (0.073) 0.254	0.003 (0.053) 0.277b	0.001 (0.047) 0.268d	0.002 (0.038) 0.252e
Human Feces			0.002 (0.051) 0.268c	0.002 (0.054) 0.225
Micromammals				0.000 (0.011) 0.052
Sixteenth century human (n = 20), dog (n = 21)				
Maize	0.410 (0.461) 0.506	0.315 (0.412) 0.485	0.231 (0.362) 0.455 c	0.222 (0.351) 0.444
Terrestrial	0.003 (0.046) 0.152	0.029 (0.290) 0.434 b	0.046 (0.289) 0.421 d	0.038 (0.314) 0.456e
High δ^15^N Fish	0.070 (0.282) 0.395a	0.002 (0.041) 0.127	0.002 (0.031) 0.109	0.001 (0.025) 0.098
Med. δ^15^N Fish	0.003 (0.117) 0.409a	0.004 (0.064) 0.206	0.002 (0.045) 0.173	0.001 (0.034) 0.146
Low δ^15^N Fish	0.002 (0.062) 0.214	0.007 (0.167) 0.517b	0.004 (0.123) 0.479 d	0.04 (0.108) 0.470e
Human Feces			0.005 (0.097) 0.364 c	0.005 (0.097) 0.364
Micromammals				0.001 (0.027) 0.110
Seventeenth century human (n = 16), dog (n = 41)				
Maize	0.414 (0.470) 0.522	0.491 (0.538) 0.574	0.446 (0.514) 0.559c	0.443 (0.511) 0.556d
Terrestrial	0.002 (0.037) 0.126	0.189 (0.363) 0.432 b	0.221 (0.354) 0.420d	0.213 (0.361) 0.424e
High δ^15^N Fish	0.107 (0.308) 0.429a	0.000 (0.013) 0.048	0.000 (0.011) 0.045	0.000 (0.009) 0.040
Med. δ^15^N Fish	0.005 (0.130) 0.359a	0.000 (0.019) 0.077	0.001 (0.017) 0.070	0.000 (0.014) 0.061
Low δ^15^N Fish	0.003 (0.054) 0.183	0.002 (0.050) 0.271 b	0.002 (0.037) 0.214d	0.001 (0.033) 0.219e
Human Feces			0.002 (0.042) 0.166c	0.002 (0.042) 0.166d
Micromammals				0.000 (0.010) 0.042

Letters adjacent to ranges indicate sources with strong negative correlations.

**Table 3:** Dog and human MixSIAR model 2.5% (50.0%) 97.5% quantiles by sites.

Source	Human	Dog Model 1	Dog Model 2	Dog Model 3
Fairty ossuary human (n = 8)-Robb village dog (n = 9), fourteenth century AD				
Maize	0.402 (0.517) 0.607	0.375 (0.468) 0.560	0.200(0.388) 0.531	0.187 (0.393) 0.532c
Terrestrial	0.003 (0.072) 0.243	0.042 (0.326) 0.498a	0.020 (0.266) 0.477	0.030 (0.260) 0.445d
High δ^15^N Fish	0.010 (0.142) 0.228	0.001 (0.024) 0.094	0.001 (0.024) 0.111	0.000 (0.021) 0.101
Med. δ^15^N Fish	0.005 (0.132) 0.385	0.002 (0.039) 0.165	0.001 (0.040) 0,183	0.001 (0.035) 0.159
Low δ^15^N Fish	0.003 (0.095) 0.311	0.005 (0.112) 0.445a	0.004 (0.121) 0.437	0.004 (0.100) 0.416d
Human Feces			0.004 (0.095) 0.392	0.006 (0.089) 0.371c
Micromammals				0.001 (0.029) 0.140
Kleinberg ossuary human (n = 12)-Seed Barker village dog (n = 5), sixteenth century AD				
Maize	0.381 (0.457) 0.526	0.268 (0.442) 0.578	0.200 (0.388) 0.531	0.082 (0.364) 0.535
Terrestrial	0.002 (0.054) 0.177	0.016 (0.213) 0.465b	0.020 (0.266) 0.477	0.010 (0.245) 0.372
High δ^15^N Fish	0.069 (0.264) 0.382a	0.002 (0.048) 0.211	0.001 (0.024) 0.111	0.001 (0.037) 0.182
Med. δ^15^N Fish	0.006 (0.125) 0.406a	0.003 (0.077) 0.321	0.001 (0.040) 0.183	0.002 (0.054) 0.279
Low δ^15^N Fish	0.003 (0.067) 0.225	0.07 (0.152) 0.470b	0.004 (0.121) 0.437	0.005 (0.127) 0.411
Human Feces			0.004 (0.095) 0.392	0.004 (0.094) 0.406
Micromammals				0.002 (0.053) 0.288
Ball village human (n = 6), dog (n = 12). sixteenth century AD				
Maize	0.271 (0.424) 0.540	0.183 (0.313) 0.430	0.100 (0.259) 0.393	0.100 (0.262) 0.393
Terrestrial	0.004 (0.083) 0.316	0.018 (0.300) 0.556a	0.026 (0.271) 0.509b	0.033 (0.272) 0.472c
High δ^15^N Fish	0.010 (0.182) 0.397	0.002 (0.052) 0.169	0.002 (0.040) 0.146	0.001 (0.033) 0.133
Med. δ^15^N Fish	0.006 (0.140) 0.449	0.004 (0.089) 0.295	0.003 (0.063) 0.247	0.002 (0.056) 0.219
Low δ^15^N Fish	0.004 (0.099) 0.389	0.009 (0.205) 0.592a	0.008 (0.177) 0.533b	0.006 (0.147) 0.506
Human Feces			0.006 (0.112) 0.435	0.005 (0.100) 0.406
Micromammals				0.001 (0.043) 0.179
Kelly-Campbell human (n = 6), dog (n = 8), seventeenth century AD				
Maize	0.305 (0.452) 0.576	0.462 (0.576) 0.662	0.322(0.497) 0.611	0.287 (0.506) 0.621
Terrestrial	0.001 (0.041) 0.180	0.043 (0.248) 0.375a	0.017 (0.235) 0.405b	0.030 (0.234) 0.365c
High δ^15^N Fish	0.049 (0.291) 0.480	0.001 (0.023) 0.100	0.001 (0.020) 0.104	0.001 (0.018) 0.095
Med. δ^15^N Fish	0.006 (0.113) 0.405	0.002 (0.039) 0.162	0.001 (0.034) 0.158	0.001 (0.029) 0.155
Low δ^15^N Fish	0.003 (0.096) 0.343	0.005 (0.085) 0.349a	0.003 (0.093) 0.359b	0.002 (0.071) 0.332c
Human Feces			0.003 (0.068) 0.305	0.003 (0.061) 0.284d
Micromammals				0.001 (0.022) 0.126
Ossossané ossuary human (n = 5)-Ossossané village dog (n = 11), seventeenth century AD				
Maize	0.297 (0.469) 0.590	0.451 (0.542) 0.618	0.295 (0.482) 0.587b	0.291 (0.473) 0.583c
Terrestrial	0.003 (0.069) 0.278	0.007 (0.173) 0.339a	0.016 (0.178) 0.325c	0.015 (0.166) 0.300d
High δ^15^N Fish	0.010 (0.179) 0.390	0.001 (0.040) 0.132	0.001 (0.031) 0.118	0.001 (0.026) 0.111
Med. δ^15^N Fish	0.007 (0.126) 0.406	0.003 (0.064) 0.226	0.002 (0.049) 0.194	0.002 (0.043) 0.180
Low δ^15^N Fish	0.006 (0.061) 0.250	0.009 (0.150) 0.388a	0.003 (0.110) 0.342c	0.005 (0.105) 0.346d
Human Feces			0.003 (0.096) 0.405b	0.003 (0.085) 0.367c
Micromammals				0.001 (0.032) 0.159

Letters adjacent to ranges indicate sources with strong negative correlations.

### Century models

**Figure 1:**
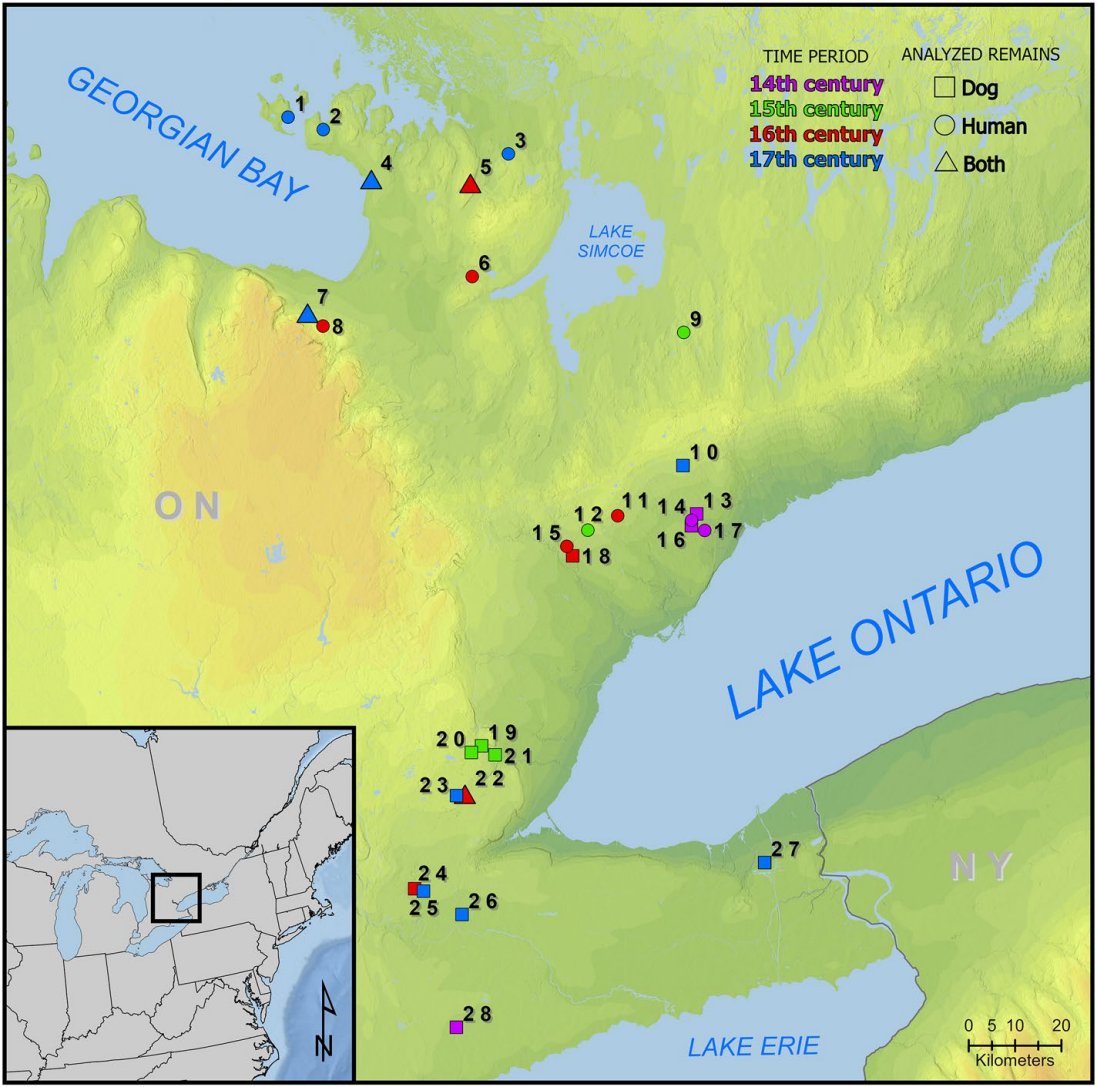
General locations of sites with dog and/or human bone collagen isotope data used in the models. 1. Christian Island, 2. Maurice Ossuary, 3. Warminster/Cahiague, 4. Ossossané Village/Ossuary, 5. Ball, 6. Carson, 7. Kelly-Campbell, 8. Milne, 9. Uxbridge Ossuary, 10. Mantle, 11. Hidden Springs, 12. Teston, 13. New, 14. Fairty Ossuary, 15. Kleinburg Ossuary, 16. Robb, 17. Staines Ossuary, 18. Seed-Barker, 19. Crawford Lake, 20. Rife, 21. Pipeline, 22. Bogle 2, 23. Hamilton, 24. Cleveland, 25. Fonger, 26. Walker, 27. Thorold, 28. Slack-Caswell.

**Figure 2:**
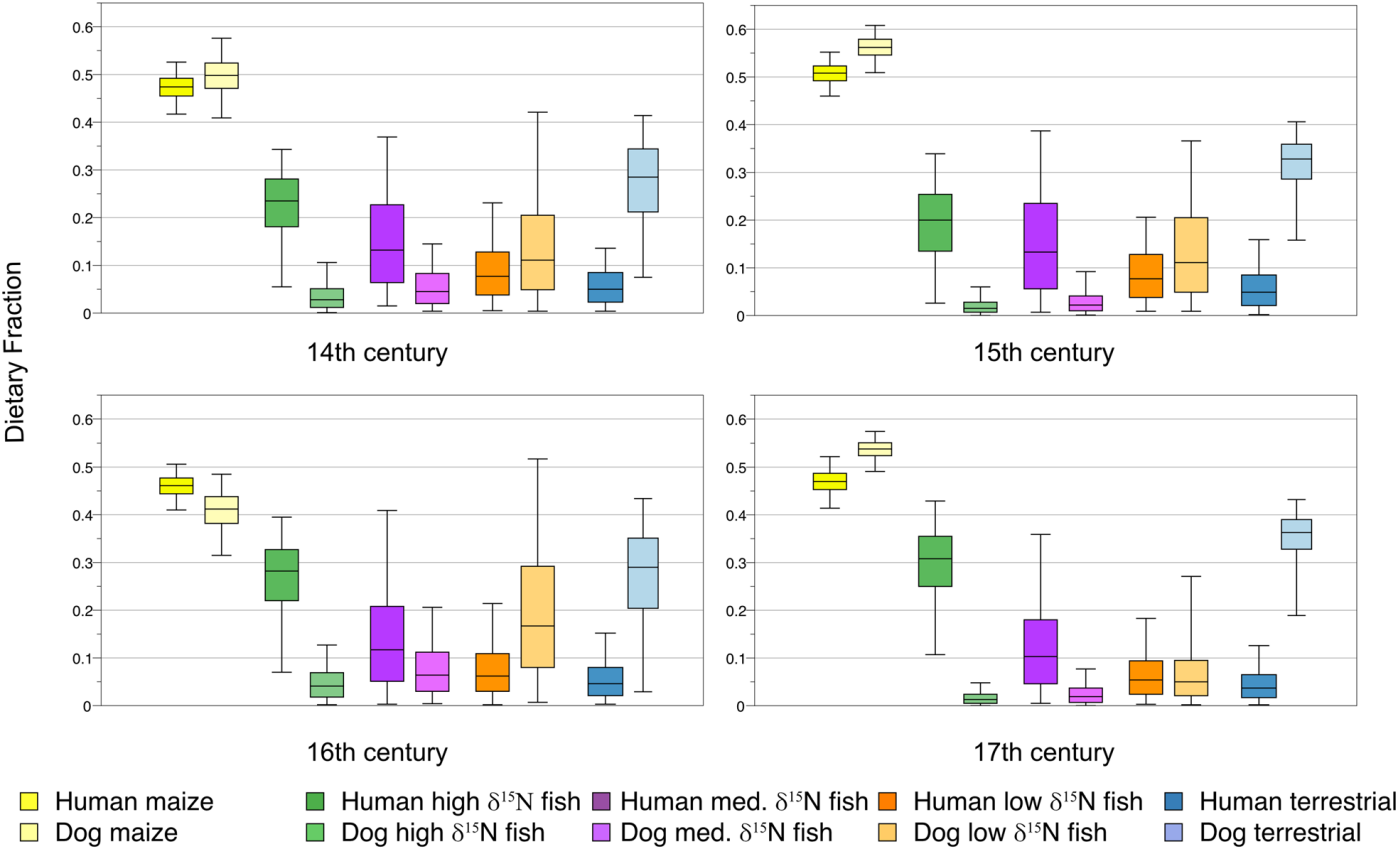
Model 1 dietary fractions of by century. Whiskers are the 2.5% and 97.5 quantiles, the box represents the 25%, 50% (median) and 75% quantiles.

Site locations are provided in Fig. 1. Consistent with previous modeling^20^, source dietary fraction estimates for humans indicate maize and high and medium *δ*^15^N fish were the primary contributors of dietary protein in each century (Fig. 2, Tables 2, S1 Table 1); high and medium *δ*^15^N fish representing high trophic-level fish. Maize dietary protein medians range from 0.461 to 0.508 (Table 2). There is a strong negative correlation (> 0.70) between high and medium *δ*^15^N fish in each of the models, indicating that the human models cannot cleanly distinguish between these resources (^38^, p. 294). In these cases, the resources may be under- or overestimated relative to one another^38^. The medians for these sources range from 0.181 to 0.308 and 0.117 to 0.132, respectively (Table 2). Terrestrial resource medians range from 0.054 to 0.077 (Table 2).

The human results contrast with Model 1 for dogs, which uses the same sources as the human models (Fig. 2, Table 2, S1 Table 1, online). As in the human models, in all dog models, maize is estimated to be the highest contributor of dietary protein in dog diets, and estimated fractions for dogs are close to those for humans. However, unlike the human models, terrestrial animals are consistently estimated to be the second largest contributor to dog dietary protein with low *δ*^15^N fish being the third largest contributor; low *δ*^15^N fish representing lower trophic-level fish than high and medium *δ*^15^N fish. In all the models there are strong negative correlations between terrestrial animals and low *δ*^15^N fish indicating they may be under- or overestimated relative to one another. Regardless, dietary fraction medians for these two sources together range from 0.285 to 0.363 and 0.50 to 0.167, respectively, which contrasts with combined human medians 0.037 to 0.050 and 0.054 to 0.077, respectively. High and medium *δ*^15^N fish are estimated to have contributed substantially lower dietary protein fractions to dogs (medians = 0.013–0.041) than in the human models. Assuming dogs only ate what humans ate, then, the models indicate substantially different dietary protein fractions for all resources other than maize, with dogs more reliant on terrestrial animal and low *δ*^15^N fish tissue and humans more reliant on high and medium *δ*^15^N fish tissue.

Because caceotrophy is a dog behavioral trait, dog Model 2 included isotope and concentration estimates for human feces (Table 3, S1 Table 2, online). Maize and terrestrial animals continue to be the primary sources of dietary protein. Human feces are estimated to be the fourth highest contributor of protein to dog diets after maize, terrestrial animals, and low *δ*^15^N fish. Estimates for low *δ*^15^N fish continue to be higher than medium and high *δ*^15^N fish individually and together. There are strong negative correlations between low δ^15^N fish and terrestrial animals in models and in the 16th-century model between maize and human feces, indicating these source pairs may be under- or overestimated relative to one another.

**Figure 3:**
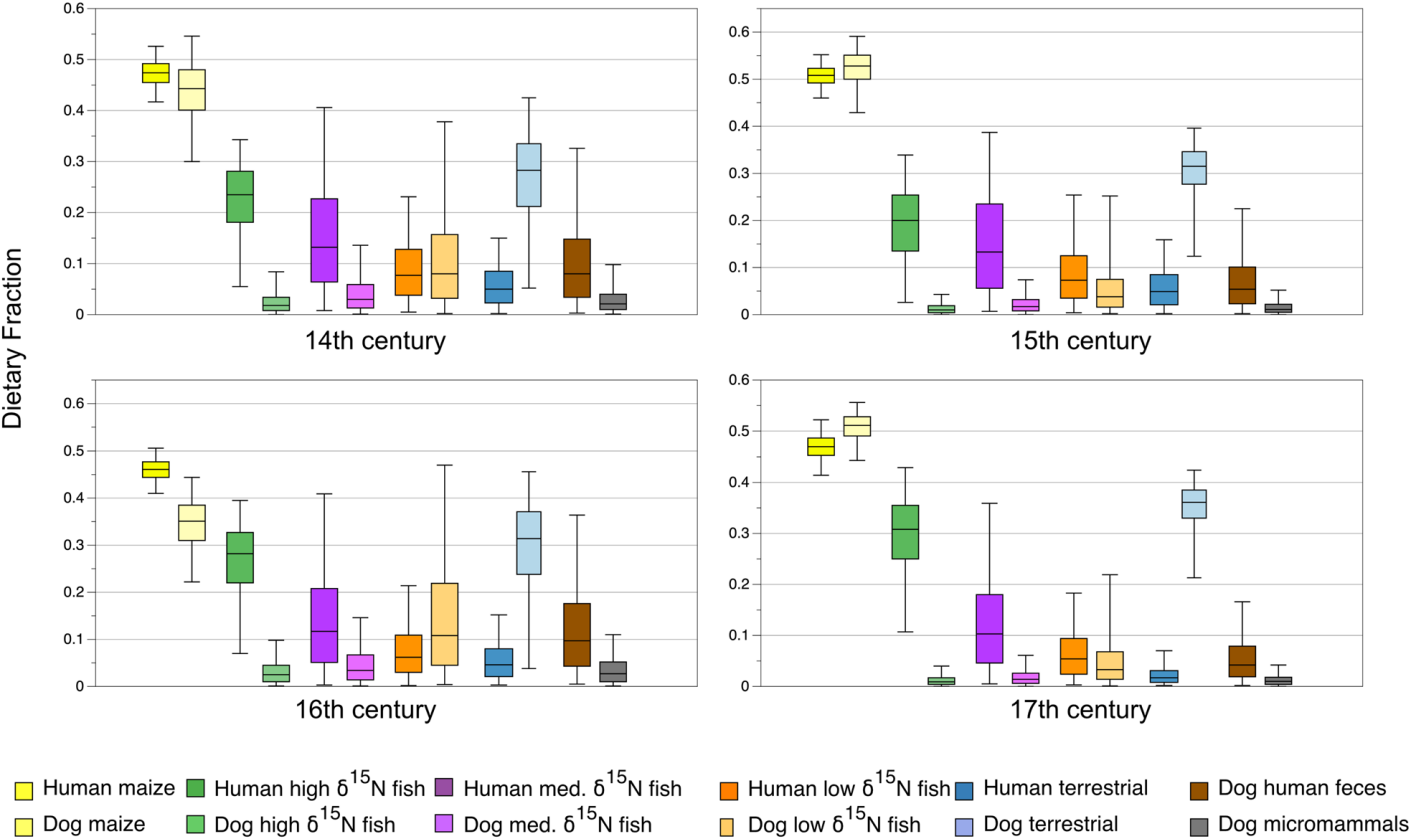
Model 3 dietary fractions of by century.

Glencross et al. (^9^, p. 7) suggest that micromammal (mice shrews, voles) consumption may account, in part, for the high *δ*^13^C ratios in dogs, with dogs hunting and consuming rodents that fed on maize stores. To assess this possibility, dog Model 3 includes micromammals with high *δ*^13^C ratios (> − 17.00‰) taken from^37^. This model has little effect on overall estimated resource fractions, with the estimate medians for micromammals ranging from 0.010 to 0.027 (Fig. 3, Table 1, S1 Table 3, online).

### Site specific models

**Figure 4:**
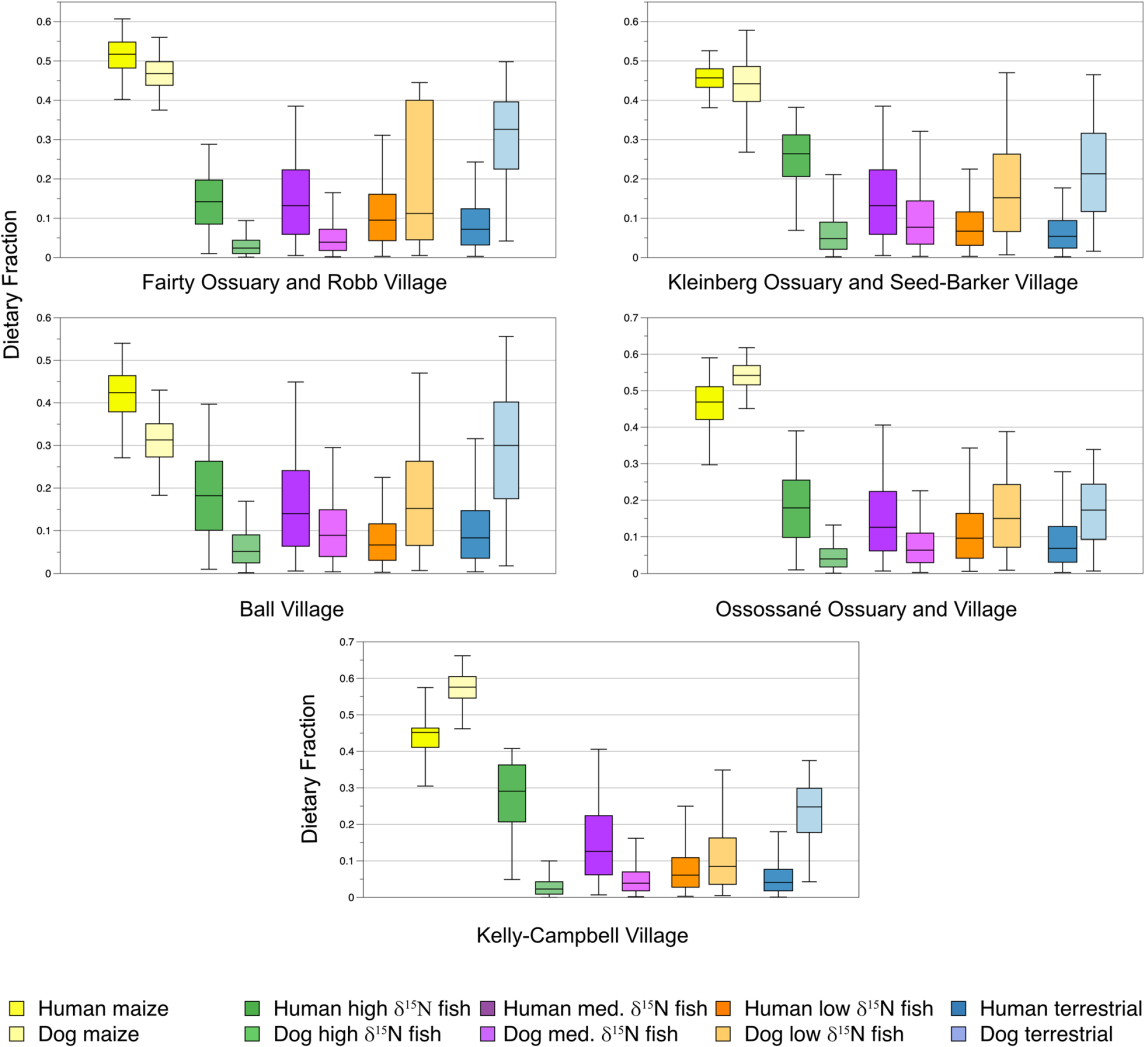
Model 1 dietary fractions of by sites.

**Figure 5:**
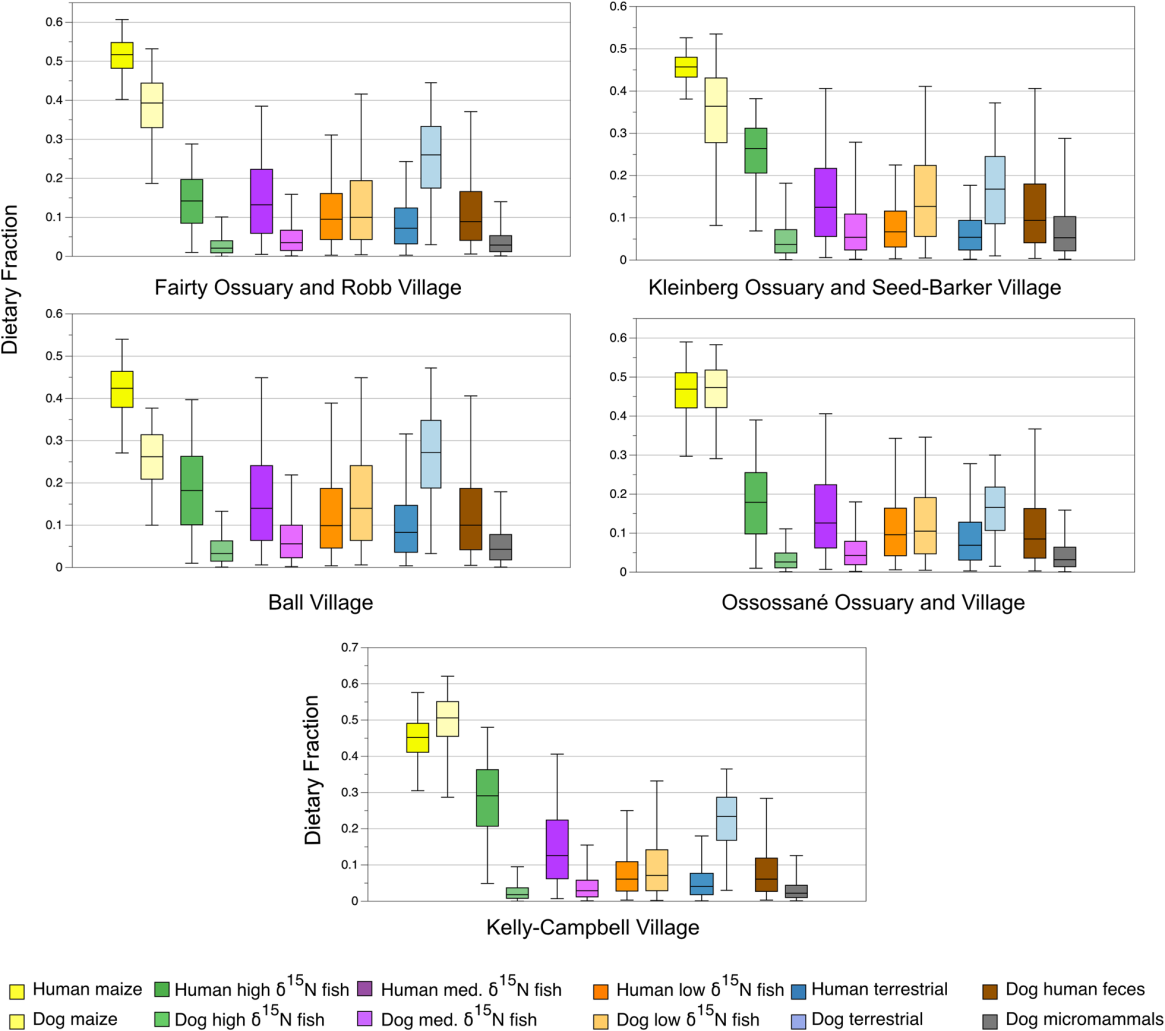
Model 3 dietary fractions of by sites.

Models for humans and dogs from the same village sites (Ball and Kelly-Campbell) or ossuaries for humans and village sites for dogs (Fairty Ossuary-Robb village; Kleinberg Ossuary-Seed-Barker village; Ossossané ossuary-Ossossané village) following Glencross and colleagues^9^ were run using the same sources. Results are presented in Figs. 4 and 5, Table 3, and S1 Tables 4, 5, 6, online. In each of the human models, maize is the highest estimated source of dietary protein, followed by high and medium *δ*^*1*5^N fish (Fig. 4, Table 3, S1 Table 4, online).

In dog Model 1 maize is estimated to be the highest contributor of dietary protein followed by terrestrial prey and low *δ*^15^N fish (Fig. 4, Table 3, S1 Table 4, online). In each model the latter two sources are highly negatively correlated and so may be under- or overestimated to one another. This ranking continues in Model 2 (Table 3, S1 Table 5, online), with human feces ranking as the fourth highest estimated source of dietary protein. In the Ossossané model maize and human feces are strongly negatively correlated and so may be under- or overestimated relatively to one another. Model 3 results in human feces as the fourth-ranked estimated source of dietary protein for each site (Fig. 5, Table 3, S1 Table 6, online). Dietary fraction estimate medians for micromammals range from 0.029 to 0.053.

In sum, while there is variation in dietary fraction between centuries and sites, the primary modeled sources of protein in human diets were maize and high and medium *δ*^15^N fish. The primary sources of protein in dog diets were maize and terrestrial animals followed by low *δ*^15^N fish and human feces.

## Discussion and conclusions

The primary assumption of CSA is that stable isotope ratios obtained on dog tissues reflect diets that are reasonably close to the diets of humans with whom the dogs lived. Glencross and associates^9^ recently performed the first CSA analysis for ancestral Iroquoian sites in southern Ontario by comparing *δ*^15^N and *δ*^13^C ratios of dog tissues with those of human tissues from related contexts. They conclude that despite small differences in isotopic ratios between dogs and humans, that dog tissue *δ*^13^C and *δ*^15^N ratios are reasonable analogs for ancestral fourteenth through seventeenth century human ratios.

Few CSA studies in North America have moved beyond direct comparison of dog and human *δ*^13^C and *δ*^15^N ratios; with few exceptions (e.g.,^8^), Bayesian dietary mixing models have not been performed. Here I have used dog and human *δ*^13^C and *δ*^15^N ratios extracted from the literature, including those recently published by Glencross and associates^9^, in Bayesian dietary mixing models to obtain estimates of the sources of protein for ancestral Iroquoians and their dogs. The results indicate variation in *δ*^13^C and *δ*^15^N ratios between humans and dogs reflect dietary differences. Human dietary protein derived primarily from maize and high and medium *δ*^15^N fish. Dog dietary protein, on the other hand, derived primarily from maize and terrestrial animals, with contributions from low *δ*^15^N fish and human feces.

Maize was the primary source of calories for ancestral Iroquoian populations in southern Ontario as reflected in isotopic analyses of human tissues^20,33,35^ and sixteenth and seventeenth-century AD ethnohistorical accounts^29,39,40^. Ethnohistorical accounts indicate that a common dish was *sagamité* a stew/soup with a maize base to which other foods were added. The comment by Sagard^29^ on dogs eating directly from human-held pots of *sagamité* indicates that the animals were accustomed to eating food prepared for human consumption. This suggests that dogs were directly fed portions of their diets by their associated humans as is typical for free-ranging dogs. It is also possible that dogs also obtained maize from food discarded by people as in contemporary rural Zimbabwe communities.

Free-ranging dogs obtain large proportions of food from opportunistic scavenging, including the carcasses of animals away from settlements and tissue scraps from human-processed carcasses. This was apparently the case for Iroquoian dogs given that terrestrial animals were consistently the second ranked estimated source of dietary protein. Given that terrestrial animals were not a primary source of dietary protein for ancestral Iroquoian individuals it is likely that dogs obtained this source through scavenging carcasses away from villages. While fish were important in both human and dog diets, humans consumed primarily high trophic-level fish (high and medium *δ*^15^N fish), while dogs consumed lower trophic-level fish (low *δ*^15^N fish). This is consistent with ethnohistorical and ethnographic records for northeastern North America, in which humans fed large proportions of harvested fish to dogs with a preference for human consumption of large fatty fish rather than small, leaner fish, which were fed to dogs^41^. Although observations for this pattern are not evident in the Iroquoian ethnohistorical record, it is a possible explanation for the differences in the model estimates. Another source of protein in dog diets was human feces. On average humans produce 29 g of fecal solids each day (^42^, p. 1854). Given that fourteenth−sixteenth century AD Iroquoian villages in southern Ontario housed hundreds to over 1000 individuals^43^, human feces were a potentially large source of dietary protein for dogs. Depending on disposal patterns, free-ranging dogs would have had ample opportunities to consume human feces. This was evidently the case given that the fourteenth-sixteenth-century models estimated median fractions range from 0.042 to 0.097 and the site-specific models estimated median fractions ranging from 0.085 to 0.124.

In conclusion, results of the Bayesian dietary mixing models indicate that dog diets were not the same as ancestral Iroquoian diets. This is consistent with what is known about contemporary free-ranging dog dietary behaviors; dogs are reliant on human-sourced food, but do not have the same diets as humans. Despite its limitations, CSA can provide important insights into human diets as has been demonstrated in numerous applications. Of particular note in eastern North America is the use of CSA to assess the presence of maize in dog diets as a potential indicator of the extent of its consumption by humans^3,6,44^. However, as the current analysis demonstrates, moving beyond standard approaches to CSA through the application of Bayesian dietary mixing models has the potential to provide more detailed assessments of dog diets in given environmental and cultural settings. To accomplish this stable isotope ratios must be available for a range of potential food sources as in the current study.

## Methods and materials

Bayesian dietary mixing models were done with MixSIAR version 3.1.11^22^ in R version 4.1.2 within R Studio version 2022.12.0 + 35 using the default MixSIAR parameters. An example model code is provided in Supplemental Data S2, online. Isotopic data for humans and dogs are *δ*^13^C and *δ*^15^N ratios measured on bone collagen obtained from the literature (S2 Tables 1 and 2, online). Collagen *δ*^13^C ratios primarily reflect the protein fraction of diets, while bone apatite and tooth enamel reflect the whole diet^23^. Therefore, dietary sources that were likely to have contributed substantively to human and dog dietary protein were used in the models. Complementary dog and human bone apatite and tooth enamel isotope ratio datasets are not available to model whole diets. Models were run for humans and dogs grouped by century and by specific site pairs. Site pairs were those used in^9^.

Samples sizes by century were: fourteenth century humans = 23, dogs = 13; fifteenth century humans = 22, dogs = 19; 16th century humans = 20, dogs = 21; seventeenth century humans = 16, dogs = 41. Human and dog samples from specific sites/site pairs were modeled separately. Sample sizes for site pairs were: Fairty Ossuary humans = 8, Robb Village dogs = 9; Kleinberg Ossuary humans = 12, Seed-Barker Village dogs = 5; Ball Village humans = 6, dogs = 12; Kelly-Campbell Village humans = 6, dogs = 8; Ossossoané Ossuary humans = 5, Ossossoané Village dogs = 11.

C and N concentrations for representative resources (S2 Table 7, online) were calculated with the following formulas:

- %C = Protein*0.52 + Carbohydrate*0.45 + Fat*0.75^45^
- %N = Protein/6.25^46^

Data for these calculations were obtained from the USDA FoodData Central database: https://fdc.nal.usda.gov/. C and N percentages for feces were calculated from^26^.

A large dataset of *δ*^13^C and *δ*^15^N ratios obtained on collagen from terrestrial animal and fish bone recovered at Iroquoian sites in southern Ontario previously compiled from the literature^20^, was used as sources in the Bayesian models (S2 Tables 3 and 4, online). Isotopic data for micromammals (mice, shrews, voles) with high *δ*^13^C ratios (> − 17‰) from Iroquoian sites in southern Ontario (S2 Table 5, online) were obtained from^37^. Following^20^ fish were separated into high, medium, and low *δ*^15^N statistically significant clusters reflecting trophic levels. High *δ*^15^N fish contain offshore species, while medium and low *δ*^15^N fish contain in-shore species (^20^, p. 7). These clusters were in the current modeling.

Given that free-ranging dogs are known to eat human feces, *δ*^13^C and *δ*^15^N estimates for human feces were used in dog models 2 and 3. Isotope ratios for human feces were estimated based on a controlled human dietary study^47^. This included a diet with animal tissue derived from fish^48^. Mean offsets between the fish and vegetable diet and feces were + 1.1 for δ^13^C and + 0.4 for *δ*^15^N (^47^, p. 393). To obtain *δ*^13^C and *δ*^15^N estimates for human feces, the TEF (5 for δ^13^C, 3 for *δ*^15^N) was subtracted from and then offsets from^47^ were added to each of the human ratios (S2 Table 1, online). Maize *δ*^13^C and *δ*^15^N were taken from^49^. Source means and concentration values are provided in S2 Table 6, online. Collagen (source) to collagen (consumer) TEFs used in the models were + 1.1‰ ± 0.2‰ for *δ*^13^C and + 3.8‰ ± 0.2‰ for *δ*^15^N; for maize and human feces the TEFs were 5‰ ± 0.1‰ for *δ*^13^C and + 3.0‰ ± 0.1‰ for *δ*^15^N^21,50^ (S2 Table 8, online). Mann–Whitney and Epps-Singleton statistical tests were done in PAST version 4.11^51^.

Models for humans included maize, terrestrial prey, and low, medium, and high *δ*^15^N fish. Three models were run for dogs. Model 1 included the same five sources as in the human models, Model 2 added human feces, and Model 3 added high *δ*^13^C micromammals per^37^.

## Supplementary Information

Supplementary Information 1.

Supplementary Information 2.

## Data Availability

All data generated or analyzed during this study are included in this published article (and its Supplementary Information files).
