# Supplementary material for: Human and dog Bayesian dietary mixing models using bone collagen stable isotope ratios from ancestral Iroquoian sites in southern Ontario
Source: Sci Rep. 2023 May 3;13:7177. doi: 10.1038/s41598-023-34216-6 (PMC10156743; doi:10.1038/s41598-023-34216-6)

Human and Dog Bayesian Dietary Mixing Models using Bone Collagen Stable Isotope Ratios from Ancestral Iroquoian Sites in Southern Ontario

Supplementary Data S1
Model Summary Statistics and Diagnostics

John P. Hart
Research and Collections Division
New York State Museum
Albany, New York 12203

Source	Consumer	Mean±1σ	2.5%	5%	25%	50%	75%	95%	97.5%
		14th century human (n=23), dog (n=13)							
High $\delta^{15}N$ Fish	Human a	0.226±0.073	0.055	0.088	0.181	0.235	0.281	0.329	0.343
	Dog	0.035±0.029	0.001	0.002	0.012	0.028	0.051	0.092	0.106
Low $\delta^{15}N$ Fish	Human	0.058±0.041	0.005	0.009	0.038	0.077	0.128	0.206	0.231
	Dog b	0.140±0.114	0.004	0.009	0.049	0.111	0.205	0.366	0.421
Maize	Human	0.473±0.028	0.417	0.427	0.455	0.474	0.492	0.518	0.526
	Dog	0.497±0.043	0.409	0.426	0.471	0.498	0.524	0.562	0.576
Medium $\delta^{15}N$ Fish	Human a	0.155±0.111	0.008	0.015	0.064	0.132	0.227	0.369	0.406
	Dog	0.057±0.046	0.002	0.004	0.020	0.045	0.083	0.145	0.162
Terrestrial	Human	0.058±0.041	0.002	0.004	0.023	0.050	0.085	0.136	0.150
	Dog b	0.272±0.099	0.041	0.075	0.212	0.285	0.344	0.414	0.429
		15th century human (n=22), dog (n=19)							
High $\delta^{15}N$ Fish	Human c	0.193±0.082	0.026	0.047	0.135	0.200	0.254	0.318	0.339
	Dog	0.019±0.016	0.000	0.001	0.007	0.015	0.028	0.052	0.060
Low $\delta^{15}N$ Fish	Human	0.088±0.067	0.004	0.008	0.035	0.073	0.125	0.219	0.254
	Dog d	0.079±0.076	0.003	0.005	0.022	0.053	0.104	0.216	0.277
Maize	Human	0.508±0.023	0.460	0.469	0.492	0.508	0.523	0.545	0.552
	Dog	0.564±0.027	0.509	0.518	0.546	0.562	0.579	0.600	0.608
Medium $\delta^{15}N$ Fish	Human c	0.154±0.112	0.007	0.013	0.056	0.133	0.235	0.363	0.387
	Dog	0.028±0.025	0.001	0.002	0.010	0.022	0.041	0.077	0.092
Terrestrial	Human	0.057±0.044	0.002	0.003	0.021	0.049	0.085	0.142	0.159
	Dog d	0.310±0.068	0.158	0.199	0.286	0.328	0.359	0.396	0.406
		16th century human (n=20), dog (n=21)							
High $\delta^{15}N$ Fish	Human e	0.268±0.082	0.070	0.112	0.220	0.282	0.327	0.379	0.395
	Dog	0.047±0.034	0.002	0.004	0.018	0.041	0.069	0.112	0.127
Low $\delta^{15}N$ Fish	Human	0.075±0.058	0.002	0.005	0.030	0.062	0.109	0.188	0.214
	Dog f	0.196±0.144	0.007	0.013	0.080	0.167	0.292	0.477	0.517
Maize	Human	0.460±0.024	0.410	0.421	0.444	0.461	0.477	0.499	0.506
	Dog	0.409±0.034	0.315	0.334	0.382	0.412	0.438	0.474	0.485
Medium $\delta^{15}N$ Fish	Human e	0.142±0.112	0.003	0.007	0.051	0.117	0.208	0.365	0.409
	Dog	0.076±0.056	0.004	0.006	0.030	0.064	0.112	0.183	0.206
Terrestrial	Human	0.055±0.041	0.003	0.005	0.021	0.046	0.080	0.135	0.152
	Dog f	0.272±0.105	0.029	0.068	0.204	0.290	0.351	0.418	0.434
		17th century human (n=16), dog (n=41)							
High $\delta^{15}N$ Fish	Human g	0.298±0.081	0.107	0.146	0.250	0.308	0.355	0.412	0.429
	Dog	0.016±0.013	0.000	0.001	0.005	0.013	0.024	0.041	0.048
Low $\delta^{15}N$ Fish	Human	0.064±0.049	0.003	0.005	0.024	0.054	0.094	0.161	0.183
	Dog h	0.070±0.072	0.002	0.004	0.021	0.050	0.095	0.205	0.271
Maize	Human	0.470±0.027	0.414	0.425	0.453	0.470	0.487	0.512	0.522
	Dog	0.527±0.020	0.491	0.504	0.524	0.538	0.551	0.568	0.574
Medium $\delta^{15}N$ Fish	Human g	0.124±0.096	0.005	0.008	0.046	0.103	0.180	0.315	0.359
	Dog	0.025±0.022	0.000	0.001	0.007	0.019	0.037	0.067	0.077
Terrestrial	Human	0.045±0.034	0.002	0.004	0.017	0.037	0.065	0.111	0.126
	Dog h	0.352±0.060	0.189	0.241	0.328	0.363	0.390	0.423	0.432

S1 Table 1. Dog and human MixSIAR Model 1 means and quantiles by century. Letters adjacent to means indicate sources with strong negative correlations (see text for explanation)

Consumer	Species	Mean±1σ	2.5%	5%	25%	50%	75%	95%	97.5%
14th century human (n=23), dog (n=13)									
High δ^{15}N Fish	Human a	0.226±0.073	0.055	0.088	0.181	0.235	0.281	0.329	0.343
	Dog	0.029±0.025	0.001	0.002	0.010	0.023	0.042	0.077	0.093
Low δ^{15}N Fish	Human b	0.058±0.041	0.005	0.009	0.038	0.077	0.128	0.206	0.231
	Dog	0.121±0.105	0.005	0.009	0.040	0.092	0.178	0.337	0.388
Maize	Human	0.473±0.028	0.417	0.427	0.455	0.474	0.492	0.518	0.526
	Dog c	0.445±0.061	0.312	0.339	0.408	0.451	0.486	0.533	0.547
Med. δ^{15}N Fish	Human a	0.155±0.111	0.008	0.015	0.064	0.132	0.227	0.369	0.406
	Dog	0.046±0.040	0.002	0.003	0.016	0.035	0.064	0.125	0.148
Terrestrial	Human	0.058±0.041	0.002	0.004	0.023	0.050	0.085	0.136	0.150
	Dog b	0.256±0.092	0.040	0.075	0.203	0.269	0.321	0.387	0.411
Human Feces	Dog c	0.103±0.088	0.003	0.007	0.034	0.080	0.148	0.272	0.326
15th century human (n=22), dog (n=19)									
High δ^{15}N Fish	Human d	0.193±0.082	0.026	0.047	0.135	0.200	0.254	0.318	0.339
	Dog	0.015±0.014	0.000	0.001	0.005	0.012	0.022	0.043	0.050
Low δ^{15}N Fish	Human	0.088±0.067	0.004	0.008	0.035	0.073	0.125	0.219	0.254
	Dog e	0.068±0.070	0.001	0.002	0.019	0.047	0.094	0.211	0.268
Maize	Human	0.508±0.023	0.460	0.469	0.492	0.508	0.523	0.545	0.552
	Dog	0.524±0.041	0.430	0.450	0.501	0.530	0.553	0.581	0.589
Med. δ^{15}N Fish	Human d	0.154±0.112	0.007	0.013	0.056	0.133	0.235	0.363	0.387
	Dog	0.025±0.022	0.001	0.002	0.008	0.019	0.036	0.069	0.084
Terrestrial	Human	0.057±0.044	0.002	0.003	0.021	0.049	0.085	0.142	0.159
	Dog e	0.299±0.064	0.136	0.178	0.270	0.310	0.342	0.379	0.391
Human Feces	Dog	0.068±0.059	0.002	0.004	0.023	0.051	0.098	0.187	0.268
16th century human (n=20), dog (n=21)									
High δ^{15}N Fish	Human f	0.268±0.082	0.070	0.112	0.220	0.282	0.327	0.379	0.395
	Dog	0.038±0.029	0.002	0.003	0.014	0.031	0.055	0.096	0.109
Low δ^{15}N Fish	Human	0.075±0.058	0.002	0.005	0.030	0.062	0.109	0.188	0.214
	Dog g	0.157±0.128	0.004	0.010	0.055	0.123	0.227	0.421	0.479
Maize	Human	0.460±0.024	0.410	0.421	0.444	0.461	0.477	0.499	0.506
	Dog h	0.356±0.058	0.231	0.249	0.319	0.362	0.397	0.442	0.455
Med. δ^{15}N Fish	Human f	0.142±0.112	0.003	0.007	0.051	0.117	0.208	0.365	0.409
	Dog	0.057±0.047	0.002	0.004	0.019	0.045	0.082	0.150	0.173
Terrestrial	Human	0.055±0.041	0.003	0.005	0.021	0.046	0.080	0.135	0.152
	Dog g	0.273±0.096	0.046	0.081	0.217	0.289	0.343	0.403	0.421
Human Feces	Dog h	0.121±0.097	0.005	0.010	0.043	0.097	0.176	0.317	0.364
17th century human (n=16), dog (n=41)									
High δ^{15}N Fish	Human i	0.298±0.081	0.107	0.146	0.250	0.308	0.355	0.412	0.429
	Dog	0.014±0.012	0.000	0.001	0.005	0.011	0.020	0.038	0.045
Low δ^{15}N Fish	Human	0.064±0.049	0.003	0.005	0.024	0.054	0.094	0.161	0.183
	Dog j	0.055±0.058	0.002	0.003	0.016	0.037	0.077	0.168	0.214
Maize	Human	0.470±0.027	0.414	0.425	0.453	0.470	0.487	0.512	0.522
	Dog k	0.510±0.029	0.446	0.458	0.491	0.514	0.531	0.553	0.559
Med. δ^{15}N Fish	Human i	0.124±0.096	0.005	0.008	0.046	0.103	0.180	0.315	0.359
	Dog	0.022±0.019	0.001	0.001	0.008	0.017	0.031	0.058	0.070
Terrestrial	Human	0.045±0.034	0.002	0.004	0.017	0.037	0.065	0.111	0.126
	Dog j	0.345±0.051	0.221	0.255	0.322	0.354	0.377	0.409	0.420
Human Feces	Dog k	0.054±0.058	0.002	0.004	0.019	0.042	0.079	0.143	0.166

S1 Table 2. MixSIAR human model 1 and dog model 2 means and quantiles by century. Letters adjacent to means indicate sources with strong negative correlations (see text for explanation

	Model	Mean±1σ	2.5%	5%	25%	50%	75%	95%	97.5%
14th century human (n=23), dog (n=13)									
High δ15N Fish	Human a	0.226±0.073	0.055	0.088	0.181	0.235	0.281	0.329	0.343
	Dog	0.024±0.022	0.000	0.001	0.008	0.018	0.034	0.070	0.084
Low δ15N Fish	Human	0.058±0.041	0.005	0.009	0.038	0.077	0.128	0.206	0.231
	Dog b	0.110±0.101	0.002	0.005	0.032	0.080	0.157	0.320	0.378
Maize	Human	0.473±0.028	0.417	0.427	0.455	0.474	0.492	0.518	0.526
	Dog c	0.437±0.061	0.300	0.329	0.401	0.443	0.480	0.529	0.546
Med. δ15N Fish	Human a	0.155±0.111	0.008	0.015	0.064	0.132	0.227	0.369	0.406
	Dog	0.040±0.036	0.001	0.002	0.013	0.030	0.059	0.113	0.136
Terrestrial	Human	0.058±0.041	0.002	0.004	0.023	0.050	0.085	0.136	0.150
	Dog b	0.269±0.094	0.052	0.086	0.212	0.283	0.335	0.404	0.425
Human Feces	Dog c	0.103±0.088	0.003	0.007	0.034	0.080	0.148	0.272	0.326
Micomammals	Dog	0.029±0.026	0.001	0.002	0.010	0.021	0.040	0.080	0.098
15th century human (n=22), dog (n=19)									
High δ15N Fish	Human d	0.193±0.082	0.026	0.047	0.135	0.200	0.254	0.318	0.339
	Dog	0.013±0.012	0.000	0.001	0.004	0.010	0.019	0.036	0.043
Low δ15N Fish	Human	0.088±0.067	0.004	0.008	0.035	0.073	0.125	0.219	0.254
	Dog e	0.058±0.064	0.002	0.004	0.016	0.038	0.075	0.186	0.252
Maize	Human	0.508±0.023	0.460	0.469	0.492	0.508	0.523	0.545	0.552
	Dog f	0.534±0.040	0.429	0.453	0.500	0.528	0.551	0.581	0.591
Med. δ15N Fish	Human d	0.154±0.112	0.007	0.013	0.056	0.133	0.235	0.363	0.387
	Dog	0.022±0.020	0.000	0.001	0.008	0.017	0.032	0.061	0.074
Terrestrial	Human	0.057±0.044	0.002	0.003	0.021	0.049	0.085	0.142	0.159
	Dog e	0.304±0.064	0.134	0.184	0.277	0.315	0.346	0.385	0.396
Human Feces	Dog f	0.070±0.061	0.002	0.004	0.023	0.054	0.101	0.192	0.225
Micomammals	Dog	0.015±0.014	0.000	0.001	0.005	0.011	0.022	0.043	0.052
16th century human (n=20), dog (n=21)									
High δ15N Fish	Human g	0.268±0.082	0.070	0.112	0.220	0.282	0.327	0.379	0.395
	Dog	0.031±0.026	0.001	0.002	0.010	0.025	0.045	0.083	0.098
Low δ15N Fish	Human	0.075±0.058	0.002	0.005	0.030	0.062	0.109	0.188	0.214
	Dog h	0.148±0.131	0.004	0.008	0.045	0.108	0.219	0.423	0.470
Maize	Human	0.460±0.024	0.410	0.421	0.444	0.461	0.477	0.499	0.506
	Dog i	0.346±0.057	0.222	0.245	0.310	0.351	0.385	0.430	0.444
Med. δ15N Fish	Human g	0.142±0.112	0.003	0.007	0.051	0.117	0.208	0.365	0.409
	Dog	0.045±0.040	0.001	0.002	0.014	0.034	0.067	0.124	0.146
Terrestrial	Human	0.055±0.041	0.003	0.005	0.021	0.046	0.080	0.135	0.152
	Dog h	0.295±0.106	0.038	0.076	0.238	0.314	0.371	0.438	0.456
Human Feces	Dog i	0.121±0.097	0.005	0.010	0.043	0.097	0.176	0.317	0.364
Micomammals	Dog	0.035±0.031	0.001	0.002	0.010	0.027	0.052	0.096	0.110
17th century human (n=16), dog (n=41)									
High δ15N Fish	Human j	0.298±0.081	0.107	0.146	0.250	0.308	0.355	0.412	0.429
	Dog	0.012±0.011	0.000	0.001	0.004	0.009	0.017	0.033	0.040
Low δ15N Fish	Human	0.064±0.049	0.003	0.005	0.024	0.054	0.094	0.161	0.183
	Dog k	0.052±0.059	0.001	0.002	0.014	0.033	0.068	0.160	0.219
Maize	Human	0.470±0.027	0.414	0.425	0.453	0.470	0.487	0.512	0.522
	Dog l	0.508±0.029	0.443	0.457	0.491	0.511	0.528	0.549	0.556
Med. δ15N Fish	Human j	0.124±0.096	0.005	0.008	0.046	0.103	0.180	0.315	0.359
	Dog	0.019±0.017	0.000	0.001	0.006	0.014	0.026	0.052	0.061
Terrestrial	Human	0.022±0.019	0.001	0.001	0.008	0.017	0.031	0.058	0.070
	Dog k	0.351±0.054	0.213	0.257	0.330	0.361	0.385	0.416	0.424
Human Feces	Dog l	0.054±0.045	0.002	0.004	0.019	0.042	0.079	0.143	0.166
Micomammals	Dog	0.013±0.012	0.000	0.001	0.004	0.010	0.018	0.036	0.042

S1 Table 3. MixSIAR human model 1and dog 3 means and quantiles by century. Letters adjacent to model sources indicate sources with strong negative correlations (see text for explanation)

Source	Consumer	Mean±1σ	2.5%	5%	25%	50%	75%	95%	97.5%
Fairty ossuary human (n=8)-Robb village dog (n=9), 14th century AD									
HighN_Fish	Human	0.142±0.076	0.010	0.019	0.085	0.142	0.197	0.266	0.288
	Dog	0.030±0.026	0.001	0.002	0.010	0.024	0.044	0.080	0.094
LowN_Fish	Human	0.111±0.085	0.003	0.007	0.043	0.095	0.161	0.275	0.311
	Dog a	0.146±0.125	0.005	0.008	0.045	0.112	0.215	0.400	0.445
Maize	Human	0.514±0.053	0.402	0.426	0.482	0.517	0.548	0.595	0.607
	Dog	0.468±0.048	0.375	0.390	0.438	0.468	0.498	0.546	0.560
MedN_Fish	Human	0.148±0.106	0.005	0.010	0.059	0.132	0.223	0.347	0.385
	Dog	0.051±0.045	0.002	0.003	0.018	0.039	0.072	0.139	0.165
Terrestrial	Human	0.085±0.065	0.003	0.007	0.032	0.072	0.124	0.211	0.243
	Dog a	0.305±0.122	0.042	0.070	0.225	0.326	0.396	0.477	0.498
Kleinberg ossuary human (n=12)-Seed Barker village dog (n=5), 16th century AD									
HighN_Fish	Human b	0.290±0.124	0.069	0.100	0.206	0.264	0.312	0.366	0.382
	Dog	0.063±0.056	0.002	0.004	0.021	0.048	0.090	0.175	0.211
LowN_Fish	Human	0.088±0.080	0.003	0.006	0.031	0.067	0.116	0.195	0.225
	Dog c	0.178±0.131	0.007	0.014	0.066	0.152	0.263	0.419	0.470
Maize	Human	0.408±0.081	0.381	0.395	0.433	0.457	0.480	0.515	0.526
	Dog	0.438±0.076	0.268	0.308	0.397	0.442	0.486	0.553	0.578
MedN_Fish	Human b	0.142±0.114	0.006	0.011	0.056	0.125	0.217	0.360	0.406
	Dog	0.101±0.087	0.003	0.006	0.034	0.077	0.144	0.272	0.321
Terrestrial	Human	0.072±0.065	0.002	0.005	0.024	0.054	0.094	0.159	0.177
	Dog c	0.220±0.126	0.016	0.028	0.117	0.213	0.316	0.431	0.465
Ball village human (n=6), dog (n=12). 16th century AD									
HighN_Fish	Human	0.185±0.107	0.010	0.021	0.101	0.182	0.263	0.364	0.397
	Dog	0.062±0.046	0.002	0.005	0.025	0.052	0.090	0.148	0.169
LowN_Fish	Human	0.127±0.104	0.004	0.010	0.046	0.099	0.187	0.332	0.389
	Dog d	0.234±0.165	0.009	0.019	0.095	0.205	0.353	0.538	0.592
Maize	Human	0.419±0.069	0.271	0.300	0.379	0.424	0.464	0.523	0.540
	Dog	0.311±0.062	0.183	0.207	0.273	0.313	0.351	0.409	0.430
MedN_Fish	Human	0.165±0.123	0.006	0.013	0.064	0.140	0.241	0.402	0.449
	Dog	0.104±0.079	0.004	0.008	0.040	0.089	0.149	0.252	0.295
Terrestrial	Human	0.104±0.087	0.004	0.007	0.036	0.083	0.147	0.274	0.316
	Dog d	0.289±0.148	0.018	0.040	0.174	0.300	0.402	0.520	0.556
Kelly-Campbell Village human (n=6), dog (n=11), 17th century AD									
HighN_Fish	Human	0.282±0.112	0.049	0.078	0.207	0.291	0.363	0.451	0.480
	Dog	0.030±0.028	0.000	0.001	0.009	0.023	0.043	0.084	0.100
LowN_Fish	Human	0.078±0.067	0.003	0.005	0.028	0.061	0.109	0.212	0.250
	Dog e	0.112±0.095	0.005	0.009	0.036	0.085	0.163	0.308	0.349
Maize	Human	0.449±0.067	0.305	0.335	0.411	0.452	0.491	0.550	0.576
	Dog	0.573±0.049	0.462	0.486	0.546	0.576	0.605	0.646	0.662
MedN_Fish	Human	0.151±0.111	0.007	0.014	0.062	0.126	0.224	0.367	0.406
	Dog	0.050±0.044	0.002	0.004	0.018	0.039	0.070	0.133	0.162
Terrestrial	Human	0.054±0.048	0.001	0.003	0.018	0.041	0.077	0.146	0.180
	Dog e	0.235±0.088	0.043	0.065	0.178	0.248	0.299	0.358	0.375
Ossossané ossuary human (n=5)-Ossossané village dog (n=11), 17th century AD									
HighN_Fish	Human	0.182±0.105	0.010	0.022	0.098	0.179	0.255	0.360	0.390
	Dog	0.047±0.037	0.001	0.003	0.018	0.040	0.068	0.116	0.132
LowN_Fish	Human	0.116±0.093	0.006	0.009	0.042	0.096	0.164	0.294	0.343
	Dog e	0.164±0.109	0.009	0.015	0.072	0.150	0.243	0.360	0.388
Maize	Human	0.463±0.074	0.297	0.336	0.421	0.469	0.511	0.570	0.590
	Dog	0.541±0.042	0.451	0.472	0.516	0.542	0.569	0.607	0.618
MedN_Fish	Human	0.151±0.111	0.007	0.014	0.062	0.126	0.224	0.367	0.406
	Dog	0.077±0.061	0.003	0.006	0.030	0.064	0.110	0.195	0.226
Terrestrial	Human	0.088±0.075	0.003	0.006	0.031	0.069	0.128	0.239	0.278
	Dog e	0.170±0.094	0.007	0.017	0.093	0.173	0.244	0.318	0.339

S1 Table 4. Dog and human MixSIAR Model 1 results means and quantiles by sites. Letters adjacent to means indicate sources with strong negative correlations (see text for explanation).

Source	Consumer	Mean±1σ	2.5%	5%	25%	50%	75%	95%	97.5%
Fairty ossuary human (n=8)-Robb village dog (n=9), 14th century AD									
High δ^{15}N Fish	Human a	0.226±0.073	0.055	0.088	0.181	0.235	0.281	0.329	0.343
	Dog	0.033±0.031	0.001	0.002	0.011	0.024	0.046	0.092	0.111
Low δ^{15}N Fish	Human	0.058±0.041	0.005	0.009	0.038	0.077	0.128	0.206	0.231
	Dog	0.151±0.123	0.004	0.009	0.049	0.121	0.226	0.393	0.437
Maize	Human	0.473±0.028	0.417	0.427	0.455	0.474	0.492	0.518	0.526
	Dog	0.382±0.083	0.200	0.234	0.332	0.388	0.438	0.508	0.531
Med. δ^{15}N Fish	Human a	0.155±0.111	0.008	0.015	0.064	0.132	0.227	0.369	0.406
	Dog	0.054±0.049	0.001	0.003	0.017	0.040	0.077	0.152	0.183
Terrestrial	Human	0.058±0.041	0.002	0.004	0.023	0.050	0.085	0.136	0.150
	Dog	0.257±0.125	0.020	0.040	0.166	0.266	0.349	0.450	0.477
Human Feces	Dog	0.124±0.106	0.004	0.008	0.041	0.095	0.178	0.328	0.392
Kleinberg ossuary human (n=12)-Seed Barker village dog (n=5), 16th century AD									
High δ^{15}N Fish	Human b	0.193±0.082	0.026	0.047	0.135	0.200	0.254	0.318	0.339
	Dog	0.054±0.049	0.002	0.003	0.018	0.040	0.077	0.152	0.185
Low δ^{15}N Fish	Human	0.088±0.067	0.004	0.008	0.035	0.073	0.125	0.219	0.254
	Dog c	0.150±0.111	0.005	0.011	0.060	0.128	0.221	0.364	0.404
Maize	Human	0.508±0.023	0.460	0.469	0.492	0.508	0.523	0.545	0.552
	Dog d	0.380±0.096	0.165	0.206	0.321	0.390	0.446	0.520	0.546
Med. δ^{15}N Fish	Human b	0.154±0.112	0.007	0.013	0.056	0.133	0.235	0.363	0.387
	Dog	0.084±0.075	0.002	0.006	0.027	0.063	0.121	0.236	0.272
Terrestrial	Human	0.057±0.044	0.002	0.003	0.021	0.049	0.085	0.142	0.159
	Dog c	0.188±0.111	0.011	0.022	0.097	0.183	0.267	0.379	0.412
Human Feces	Dog d	0.143±0.123	0.004	0.009	0.048	0.110	0.206	0.390	0.447
Ball village human (n=6), dog (n=12). 16th century AD									
High δ^{15}N Fish	Human	0.268±0.082	0.070	0.112	0.220	0.282	0.327	0.379	0.395
	Dog	0.050±0.040	0.002	0.004	0.018	0.040	0.070	0.128	0.146
Low δ^{15}N Fish	Human	0.075±0.058	0.002	0.005	0.030	0.062	0.109	0.188	0.214
	Dog e	0.202±0.147	0.008	0.015	0.080	0.177	0.302	0.483	0.533
Maize	Human	0.460±0.024	0.410	0.421	0.444	0.461	0.477	0.499	0.506
	Dog	0.255±0.073	0.100	0.128	0.208	0.259	0.305	0.368	0.393
Med. δ^{15}N Fish	Human	0.142±0.112	0.003	0.007	0.051	0.117	0.208	0.365	0.409
	Dog	0.081±0.068	0.003	0.005	0.028	0.063	0.117	0.216	0.247
Terrestrial	Human	0.055±0.041	0.003	0.005	0.021	0.046	0.080	0.135	0.152
	Dog e	0.268±0.132	0.026	0.044	0.168	0.271	0.364	0.478	0.509
Human Feces	Dog	0.144±0.118	0.006	0.012	0.051	0.112	0.208	0.380	0.435
Kelly-Campbell Village human (n=6), dog (n=11), 17th century AD									
HighN_Fish	Human	0.282±0.112	0.049	0.078	0.207	0.291	0.363	0.451	0.480
	Dog	0.028±0.028	0.001	0.002	0.008	0.020	0.039	0.083	0.104
LowN_Fish	Human	0.078±0.067	0.003	0.005	0.028	0.061	0.109	0.212	0.250
	Dog e	0.121±0.102	0.003	0.005	0.038	0.093	0.179	0.323	0.359
Maize	Human	0.449±0.067	0.305	0.335	0.411	0.452	0.491	0.550	0.576
	Dog	0.488±0.073	0.322	0.356	0.447	0.497	0.539	0.590	0.611
MedN_Fish	Human	0.151±0.111	0.007	0.014	0.062	0.126	0.224	0.367	0.406
	Dog	0.046±0.043	0.001	0.003	0.014	0.034	0.065	0.130	0.158
Terrestrial	Human	0.054±0.048	0.001	0.003	0.018	0.041	0.077	0.146	0.180
	Dog e	0.225±0.105	0.017	0.039	0.148	0.235	0.304	0.384	0.405
Human feces	Dog	0.091±0.082	0.003	0.006	0.029	0.068	0.129	0.255	0.305
Ossossané ossuary human (n=5)-Ossossané village dog (n=11), 17th century AD									
High δ^{15}N Fish	Human	0.298±0.081	0.107	0.146	0.250	0.308	0.355	0.412	0.429
	Dog	0.038±0.033	0.001	0.002	0.013	0.031	0.056	0.101	0.118
Low δ^{15}N Fish	Human	0.064±0.049	0.003	0.005	0.024	0.054	0.094	0.161	0.183
	Dog f	0.127±0.097	0.003	0.006	0.045	0.110	0.195	0.307	0.342
Maize	Human	0.470±0.027	0.414	0.425	0.453	0.470	0.487	0.512	0.522
	Dog g	0.471±0.074	0.295	0.334	0.428	0.482	0.524	0.573	0.587
Med. δ^{15}N Fish	Human	0.124±0.096	0.005	0.008	0.046	0.103	0.180	0.315	0.359
	Dog	0.063±0.053	0.002	0.004	0.022	0.049	0.090	0.165	0.194
Terrestrial	Human	0.045±0.034	0.002	0.004	0.017	0.037	0.065	0.111	0.126
	Dog f	0.173±0.085	0.016	0.029	0.109	0.178	0.237	0.306	0.325
Human Feces	Dog g	0.127±0.110	0.003	0.008	0.042	0.096	0.185	0.350	0.405

S1 Table 5. MixSIAR human model 1 and dog model 2 means and quantiles by sites. Letters adjacent to means indicate sources with strong negative correlations (see text for explanation)

Source	Consumer	Mean±1σ	2.5%	5%	25%	50%	75%	95%	97.5%
Fairty ossuary human (n=8)-Robb village dog (n=9), 14th century AD									
High δ^{15}N Fish	Human	0.142±0.076	0.010	0.019	0.085	0.142	0.197	0.266	0.288
	Dog	0.039±0.039	0.000	0.001	0.009	0.021	0.040	0.084	0.101
Low δ^{15}N Fish	Human	0.111±0.085	0.003	0.007	0.043	0.095	0.161	0.275	0.311
	Dog a	0.132±0.114	0.004	0.008	0.043	0.100	0.194	0.362	0.416
Maize	Human	0.514±0.053	0.402	0.426	0.482	0.517	0.548	0.595	0.607
	Dog b	0.383±0.087	0.187	0.225	0.330	0.393	0.444	0.512	0.532
Med. δ^{15}N Fish	Human	0.148±0.106	0.005	0.010	0.059	0.132	0.223	0.347	0.385
	Dog	0.048±0.045	0.001	0.003	0.015	0.035	0.067	0.139	0.159
Terrestrial	Human	0.085±0.065	0.003	0.007	0.032	0.072	0.124	0.211	0.243
	Dog a	0.252±0.110	0.030	0.052	0.175	0.260	0.333	0.421	0.445
Human Feces	Dog b	0.124±0.106	0.006	0.009	0.041	0.089	0.166	0.316	0.371
Micomammals	Dog	0.039±0.039	0.001	0.002	0.012	0.029	0.053	0.112	0.140
Kleinberg ossuary human (n=12)-Seed Barker village dog (n=5), 16th century AD									
High δ^{15}N Fish	Human c	0.253±0.080	0.069	0.100	0.206	0.264	0.312	0.366	0.382
	Dog	0.052±0.050	0.001	0.003	0.017	0.037	0.072	0.146	0.182
Low δ^{15}N Fish	Human	0.079±0.061	0.003	0.006	0.031	0.067	0.116	0.195	0.225
	Dog	0.150±0.113	0.005	0.011	0.056	0.127	0.224	0.372	0.411
Maize	Human	0.456±0.037	0.381	0.395	0.433	0.457	0.480	0.515	0.526
	Dog	0.348±0.116	0.082	0.131	0.278	0.364	0.431	0.511	0.535
Med. δ^{15}N Fish	Human c	0.147±0.110	0.006	0.011	0.056	0.125	0.217	0.360	0.406
	Dog	0.078±0.075	0.002	0.005	0.024	0.054	0.109	0.230	0.279
Terrestrial	Human	0.064±0.049	0.002	0.005	0.024	0.054	0.094	0.159	0.177
	Dog	0.171±0.101	0.010	0.020	0.086	0.168	0.245	0.341	0.372
Human Feces	Dog	0.125±0.109	0.004	0.008	0.041	0.094	0.180	0.345	0.406
Micomammals	Dog	0.076±0.078	0.002	0.004	0.022	0.053	0.103	0.238	0.288
Ball village human (n=6), dog (n=12). 16th century AD									
High δ^{15}N Fish	Human	0.185±0.107	0.010	0.021	0.101	0.182	0.263	0.364	0.397
	Dog	0.043±0.037	0.001	0.003	0.015	0.033	0.063	0.115	0.133
Low δ^{15}N Fish	Human	0.127±0.104	0.004	0.010	0.046	0.099	0.187	0.332	0.389
	Dog d	0.179±0.139	0.006	0.011	0.065	0.147	0.266	0.446	0.506
Maize	Human	0.419±0.069	0.271	0.300	0.379	0.424	0.464	0.523	0.540
	Dog	0.258±0.076	0.100	0.129	0.209	0.262	0.314	0.377	0.393
Med. δ^{15}N Fish	Human	0.165±0.123	0.006	0.013	0.064	0.140	0.241	0.402	0.449
	Dog	0.071±0.060	0.002	0.004	0.023	0.056	0.100	0.192	0.219
Terrestrial	Human	0.104±0.087	0.004	0.007	0.036	0.083	0.147	0.274	0.316
	Dog d	0.265±0.115	0.033	0.055	0.188	0.272	0.348	0.445	0.472
Human Feces	Dog	0.129±0.111	0.005	0.008	0.042	0.100	0.187	0.351	0.406
Micomammals	Dog	0.055±0.049	0.001	0.003	0.018	0.043	0.078	0.150	0.179
Kelly-Campbell Village human (n=6), dog (n=11), 17th century AD									
HighN_Fish	Human	0.282±0.112	0.049	0.078	0.207	0.291	0.363	0.451	0.480
	Dog	0.026±0.027	0.000	0.001	0.008	0.018	0.037	0.077	0.095
LowN_Fish	Human	0.078±0.067	0.003	0.005	0.028	0.061	0.109	0.212	0.250
	Dog e	0.098±0.089	0.002	0.005	0.029	0.071	0.142	0.280	0.332
Maize	Human	0.449±0.067	0.305	0.335	0.411	0.452	0.491	0.550	0.576
	Dog	0.495±0.083	0.287	0.345	0.455	0.506	0.551	0.604	0.621
MedN_Fish	Human	0.151±0.111	0.007	0.014	0.062	0.126	0.224	0.367	0.406
	Dog	0.042±0.042	0.001	0.002	0.012	0.029	0.058	0.124	0.155
Terrestrial	Human	0.054±0.048	0.001	0.003	0.018	0.041	0.077	0.146	0.180
	Dog e	0.222±0.088	0.030	0.053	0.168	0.234	0.287	0.347	0.365
Human feces	Dog	0.084±0.077	0.003	0.005	0.027	0.061	0.119	0.231	0.284
Micomammals	Dog	0.033±0.036	0.001	0.002	0.010	0.022	0.044	0.098	0.126
Ossossané ossuary human (n=5)-Ossossané village dog (n=11), 17th century AD									
High δ^{15}N Fish	Human	0.182±0.105	0.010	0.022	0.098	0.179	0.255	0.360	0.390
	Dog	0.034±0.030	0.001	0.002	0.011	0.026	0.049	0.091	0.111
Low δ^{15}N Fish	Human	0.116±0.093	0.006	0.009	0.042	0.096	0.164	0.294	0.343
	Dog e	0.126±0.096	0.005	0.009	0.047	0.105	0.191	0.305	0.346
Maize	Human	0.463±0.074	0.297	0.336	0.421	0.469	0.511	0.570	0.590
	Dog f	0.463±0.076	0.291	0.326	0.422	0.473	0.518	0.567	0.583
Med. δ^{15}N Fish	Human	0.151±0.111	0.007	0.014	0.062	0.126	0.224	0.367	0.406
	Dog	0.056±0.048	0.002	0.003	0.019	0.043	0.079	0.153	0.180
Terrestrial	Human	0.088±0.075	0.003	0.006	0.031	0.069	0.128	0.239	0.278
	Dog e	0.162±0.077	0.015	0.029	0.107	0.166	0.218	0.285	0.300
Human Feces	Dog f	0.112±0.099	0.003	0.006	0.036	0.085	0.163	0.309	0.367
Micomammals	Dog	0.045±0.044	0.001	0.003	0.014	0.032	0.064	0.129	0.159

S1 Table 6. MixSIAR human model 1 and dog model 3 means and quantiles by site. Letters adjacent to model sources indicate sources with strong negative correlations (see text for explanation)

14th Century Human Model Diagnostics (n=23)

Gelman Diagnostic (variables=29)

>1.01	>1.05	>1.10
0	0	0

Geweke diagnostic (variables=29)

Chain 1	Chain 2	Chain 3
1	0	0

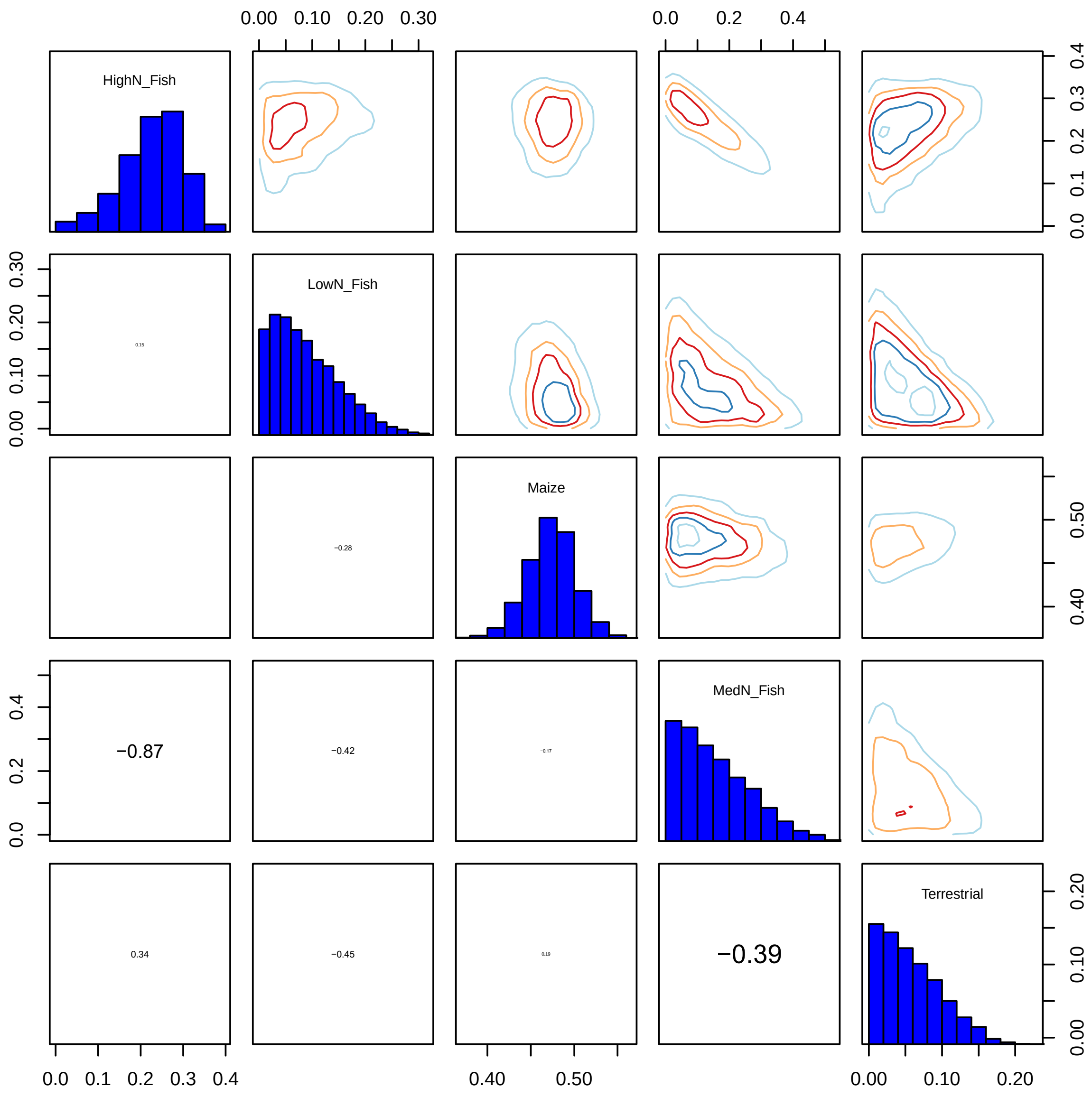

14th Century Dogs Model 1 Diagnostics (n=13)

Gelman Diagnostic (variables=19)

>1.01	>1.05	>1.10
1	0	0

Geweke diagnostic (variables=19)

Chain 1	Chain 2	Chain 3
0	0	0

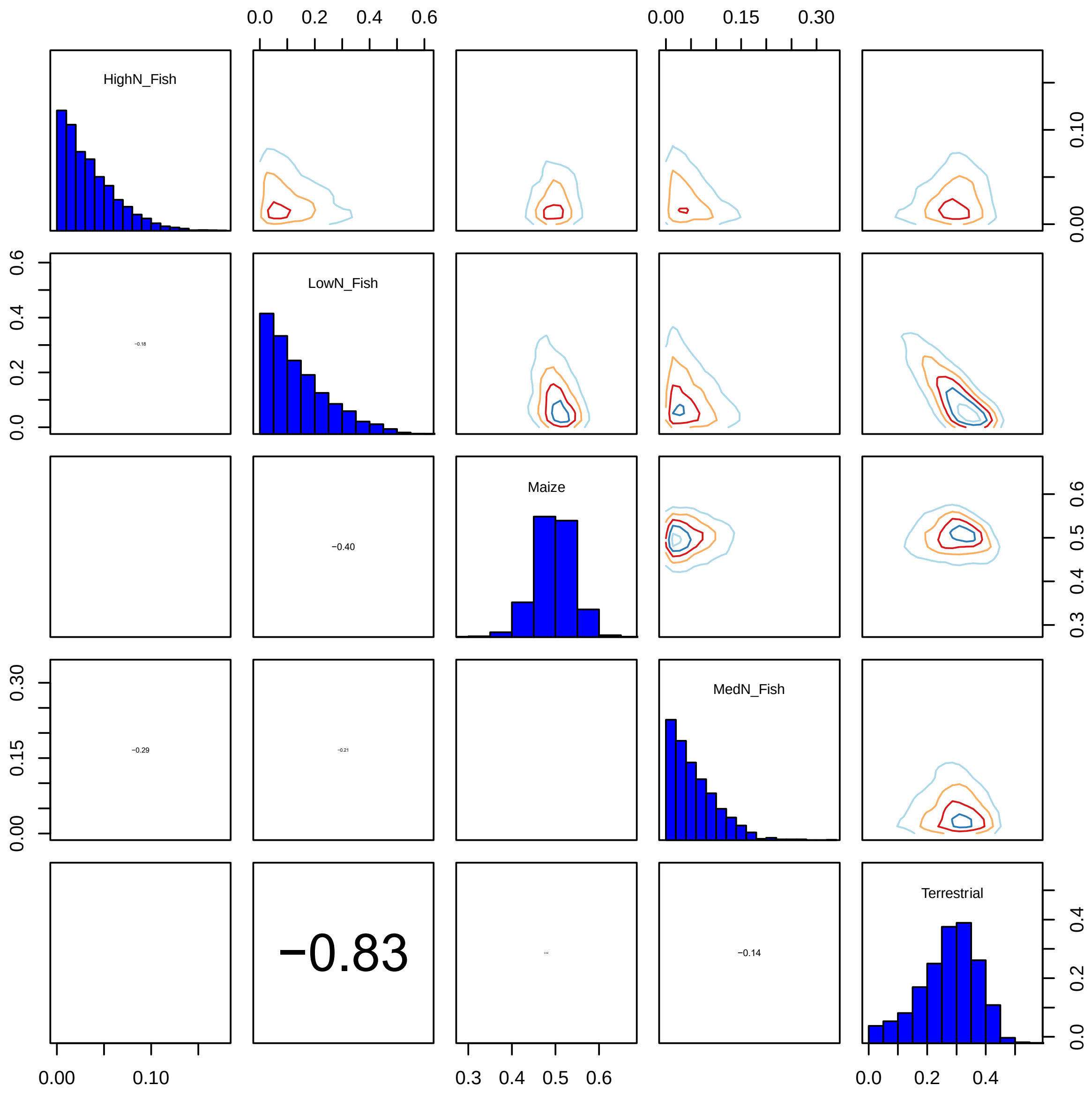

14th Century Dog Model 2 Diagnostics (n=13)

Gelman Diagnostic (variables=20)

>1.01	>1.05	>1.10
0	0	0

Geweke diagnostic (variables=20)

Chain 1	Chain 2	Chain 3
0	0	0

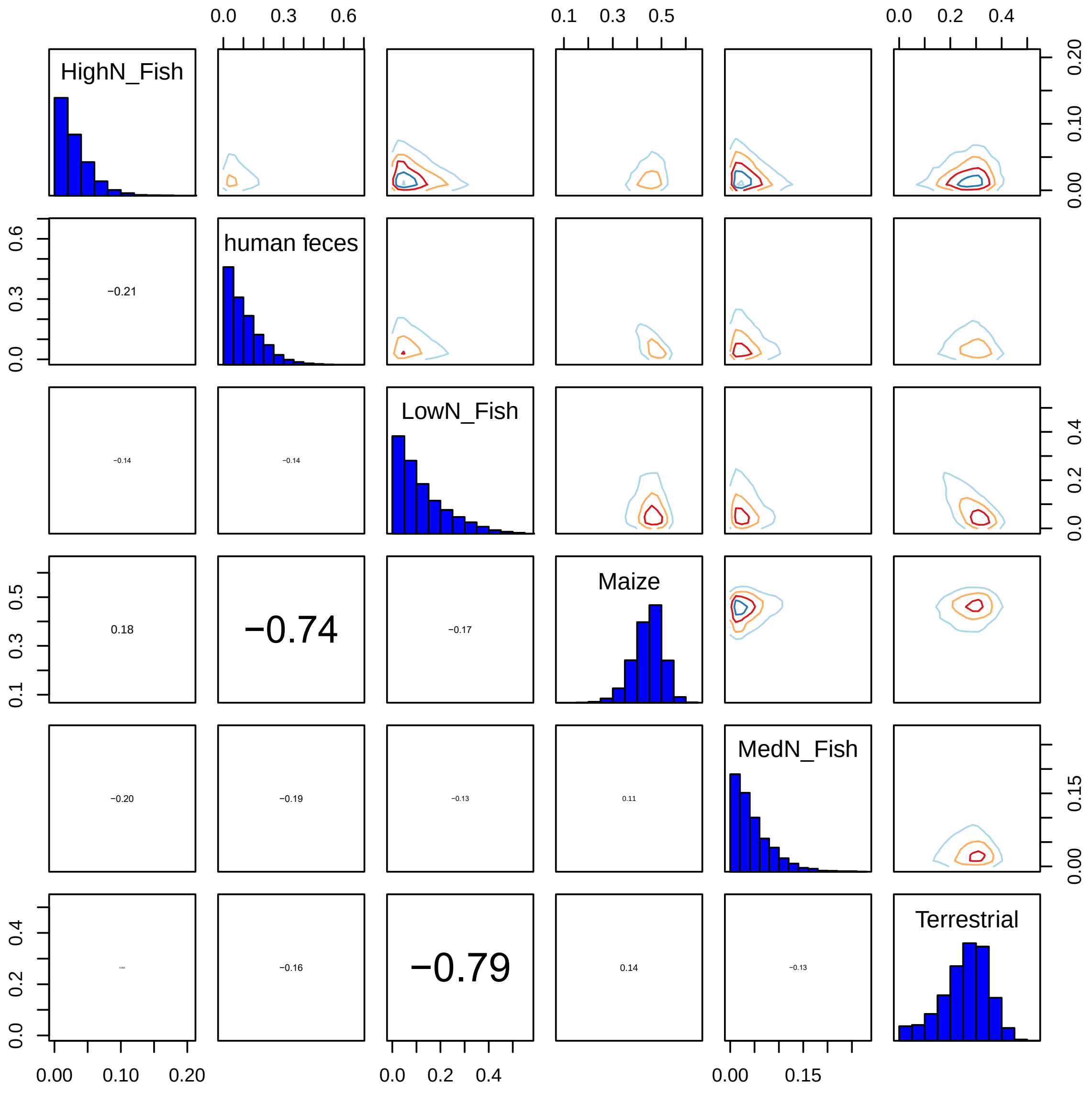

14th Century Dogs Model 3 Diagnostics (n=13)

Gelman Diagnostic (variables=21)

>1.01	>1.05	>1.10
2	0	0

Geweke diagnostic (variables=21)

Chain 1	Chain 2	Chain 3
0	0	0

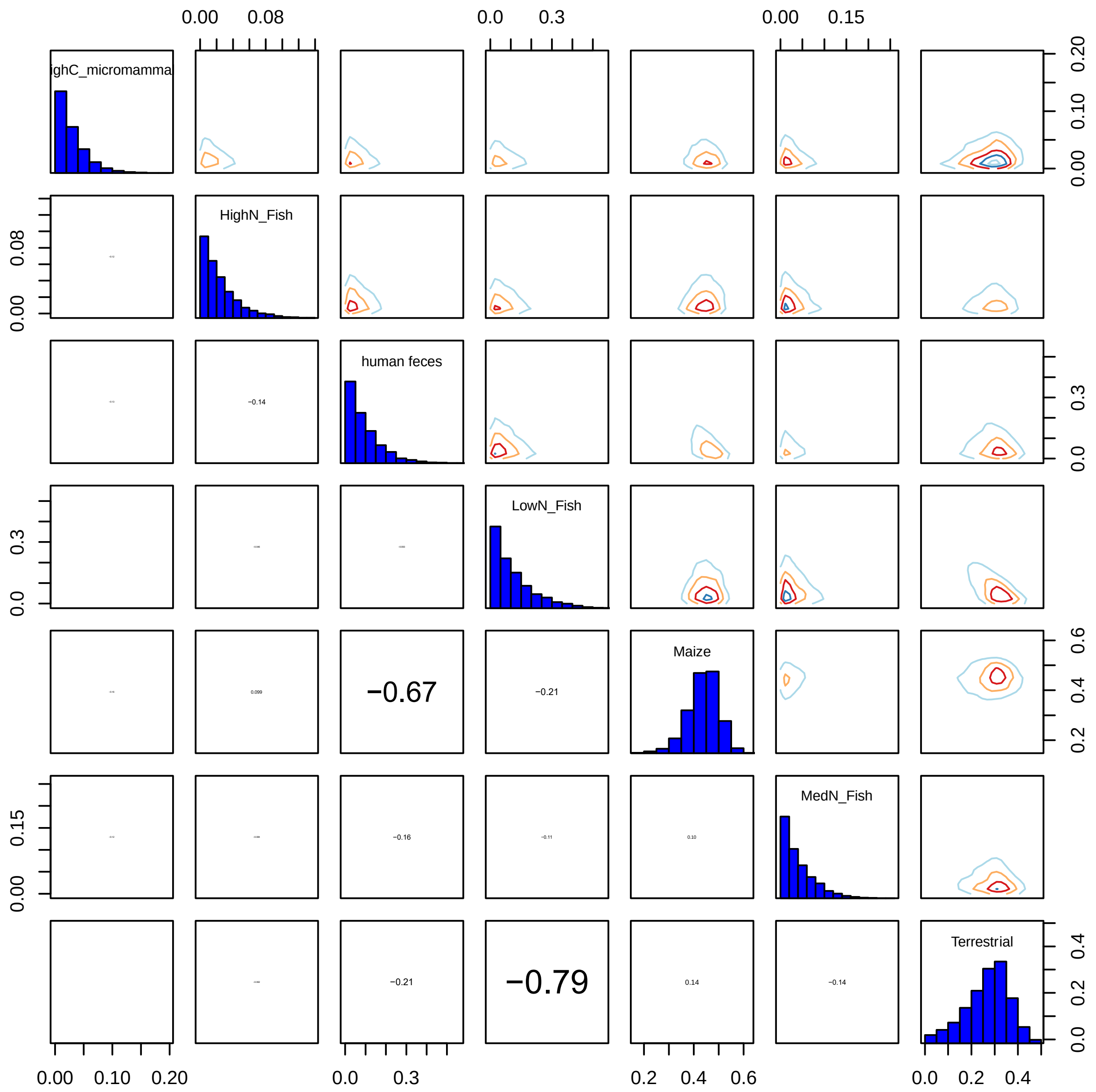

15th Century Human Model Diagnostics (n=22)

Gelman Diagnostic (variables=29)

>1.01	>1.05	>1.10
3	0	0

Geweke diagnostic (variables=29)

Chain 1	Chain 2	Chain 3
1	1	0

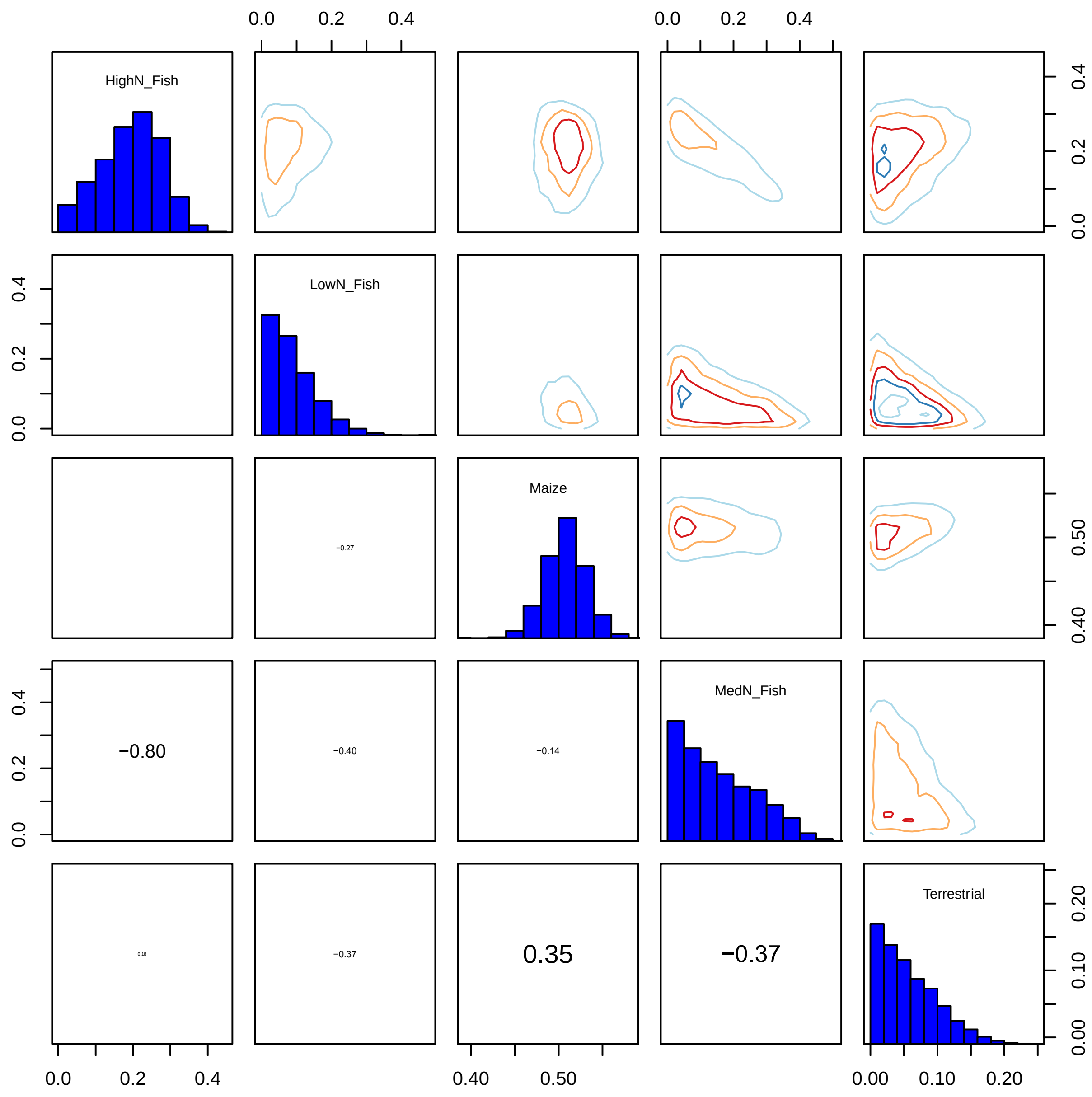

15th Century Dog Model 1 Diagnostics (n=19)

Gelman Diagnostic (variables=25)

>1.01	>1.05	>1.10
0	0	0

Geweke diagnostic (variables=26)

Chain 1	Chain 2	Chain 3
0	0	1

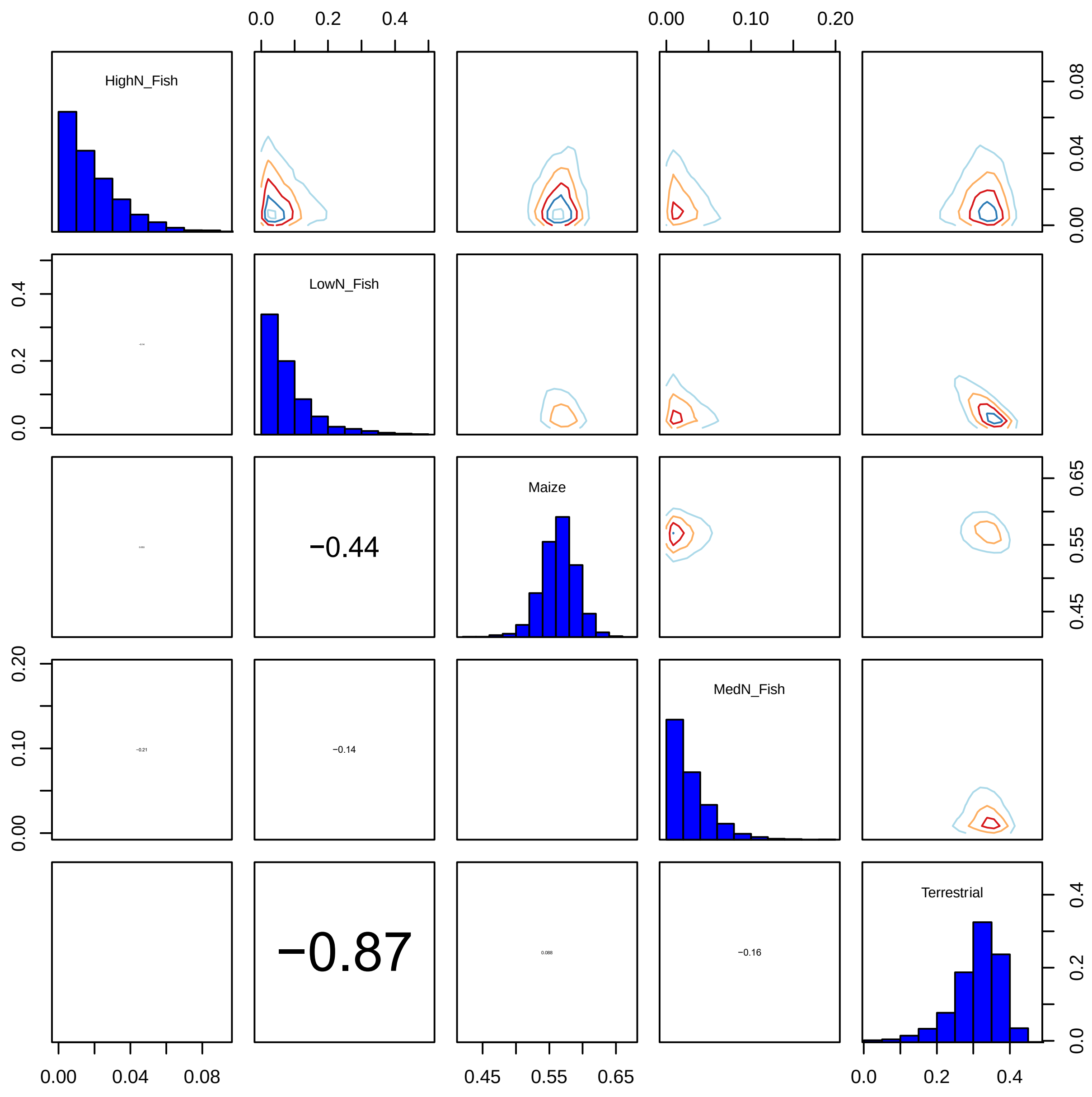

15th Century Dog Model 2 Diagostics (n=19)

Gelman Diagnostic (variables=26)

>1.01	>1.05	>1.10
0	0	0

Geweke diagnostic (variables=26)

Chain 1	Chain 2	Chain 3
0	1	0

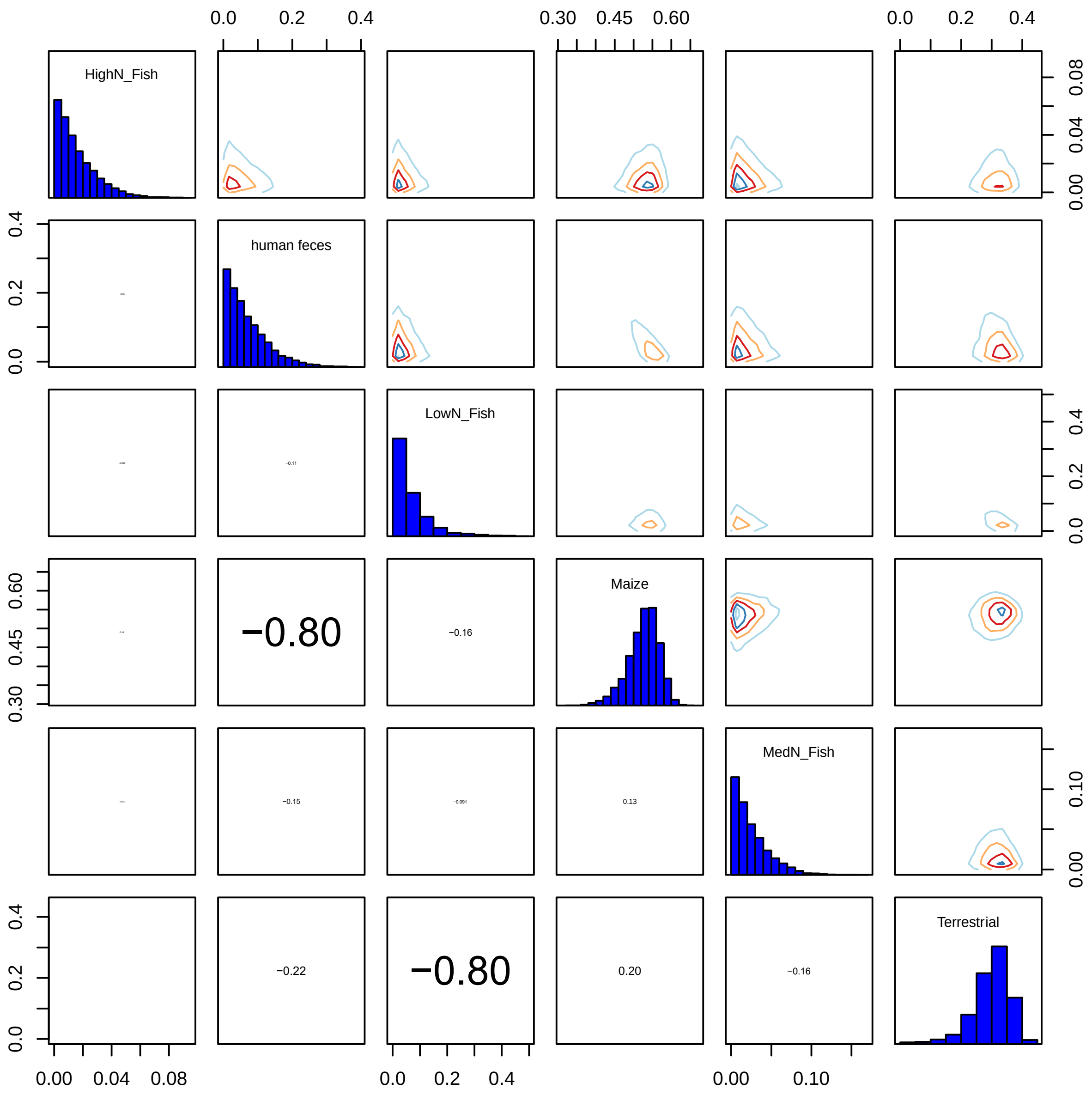

15th Century Dog Model 3 Diagnostics (n=19)

Gelman Diagnostic (variables=27)

>1.01	>1.05	>1.10
0	0	0

Geweke diagnostic (variables=28)

Chain 1	Chain 2	Chain 3
0	0	0

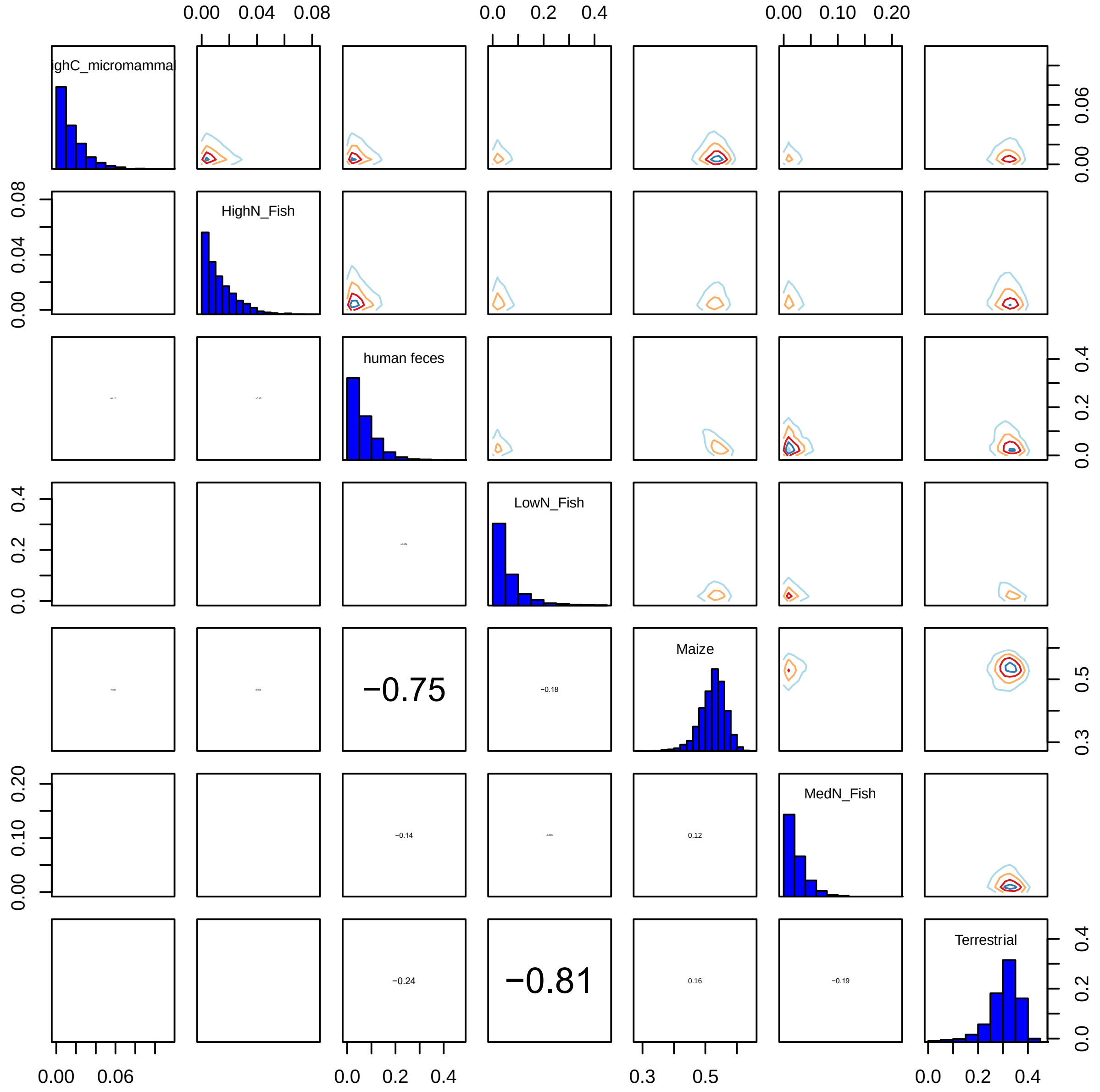

16th Century Human Model Diagnostics (n=20)

Gelman Diagnostic (variables=26)

>1.01	>1.05	>1.10
1	0	0

Geweke diagnostic (variables=26)

Chain 1	Chain 2	Chain 3
0	0	0

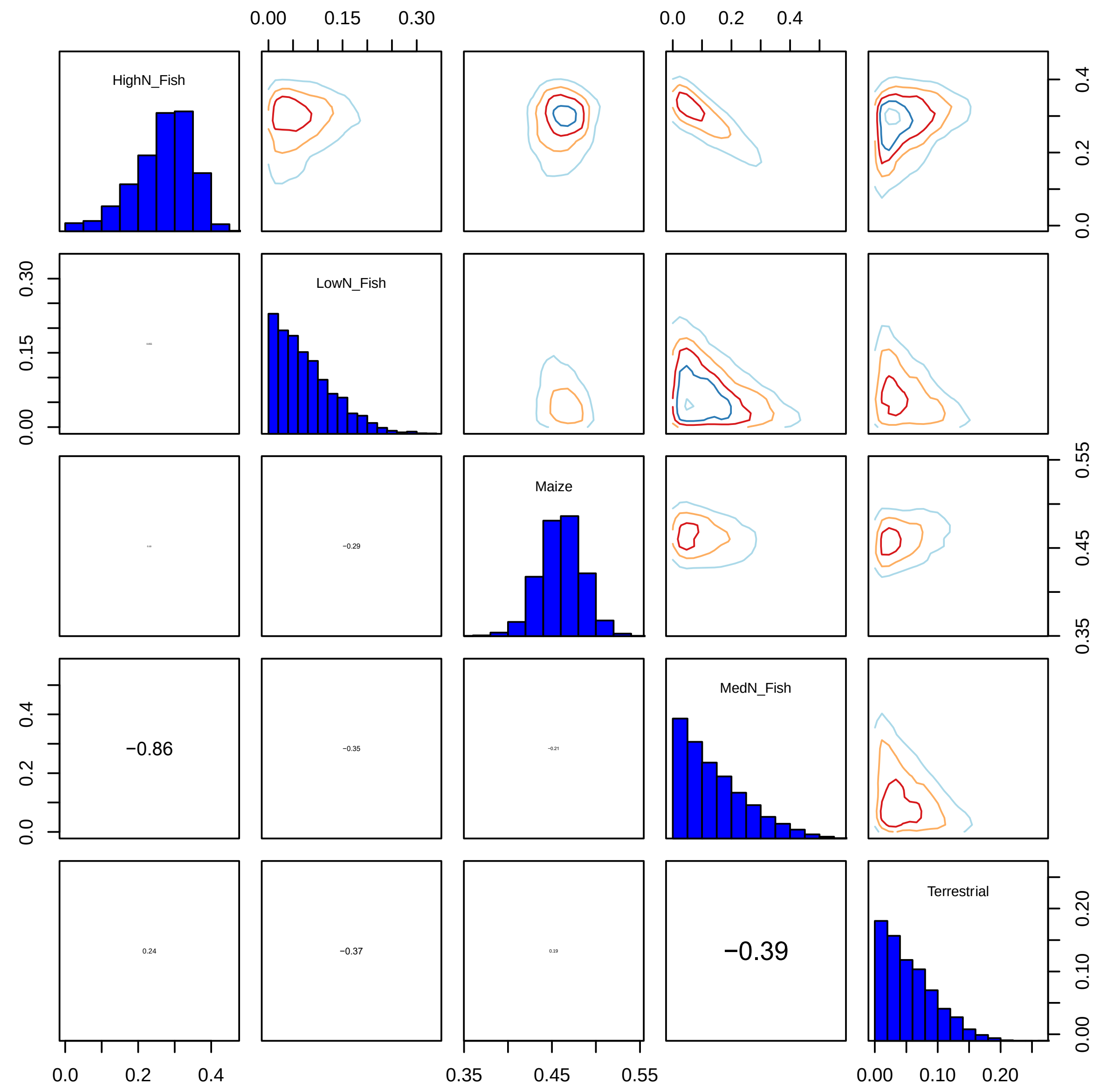

16th Century Dog Model 1 Diagnostics (n=21)

Gelman Diagnostic (variables=27)

>1.01	>1.05	>1.10
0	0	0

Geweke diagnostic (variables=27)

Chain 1	Chain 2	Chain 3
0	0	0

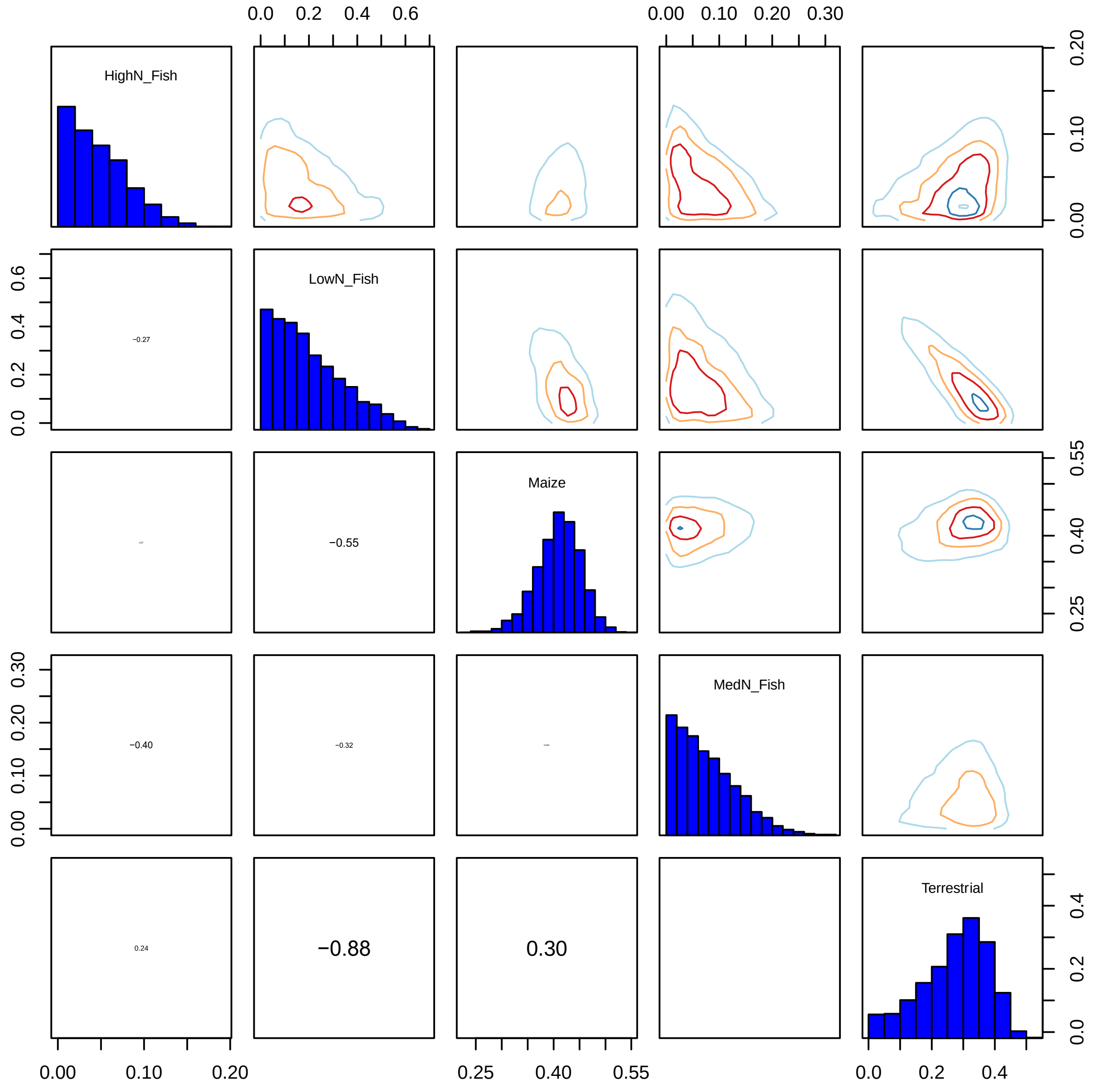

16th Century Dog Model 2 Diagnostics (n=21)

Gelman Diagnostic (variables=28)

>1.01	>1.05	>1.10
1	0	0

Geweke diagnostic (variables=28)

Chain 1	Chain 2	Chain 3
1	1	1

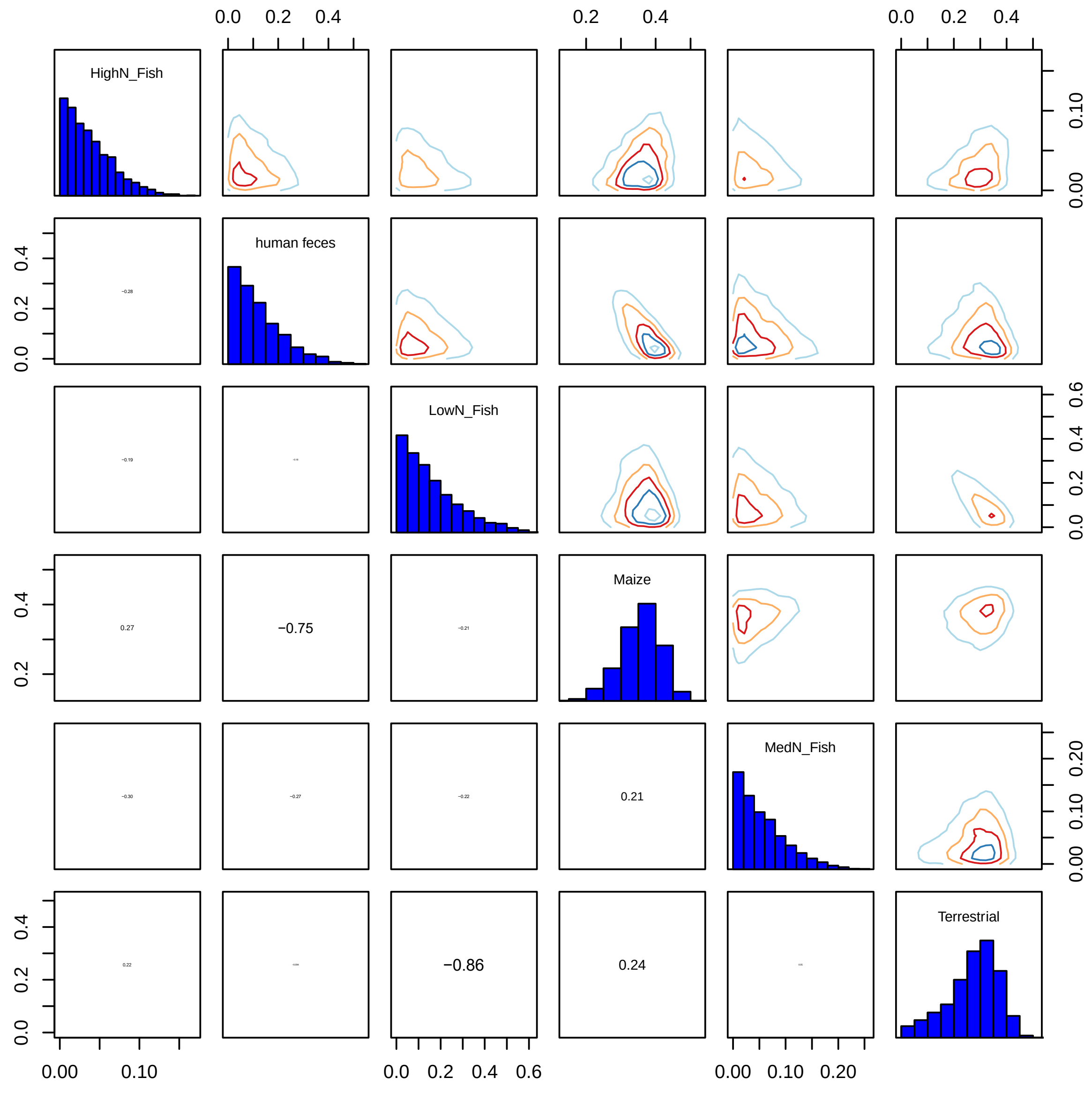

16th Century Dog Model 3 Diagnostics (n=21)

Gelman Diagnostic (variables=29)

>1.01	>1.05	>1.10
3	0	0

Geweke diagnostic (variables=29)

Chain 1	Chain 2	Chain 3
0	0	0

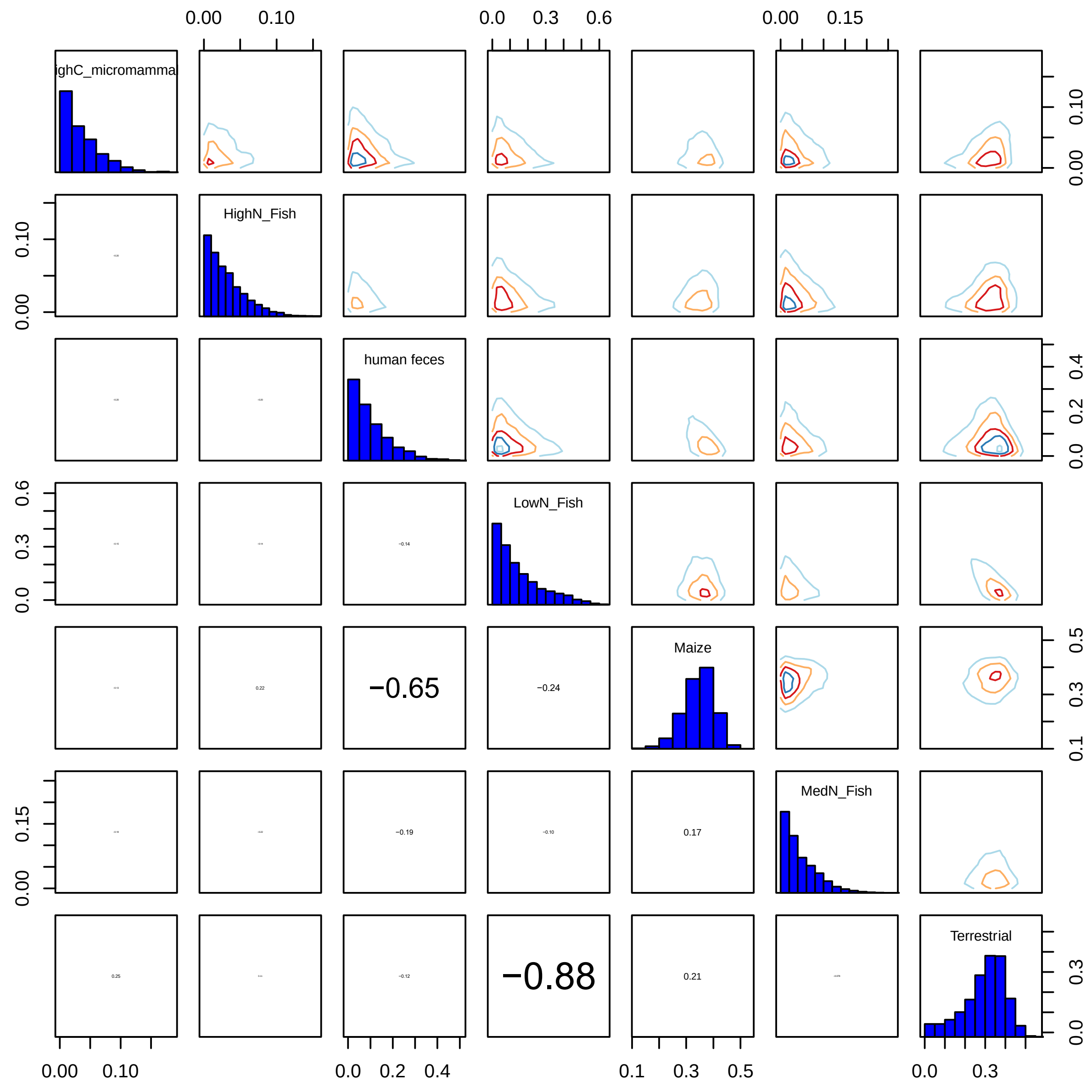

17th Century Human Model Diagnostics (n=16)

Gelman Diagnostic (variables=22)

>1.01	>1.05	>1.10
0	0	0

Geweke diagnostic (variables=22)

Chain 1	Chain 2	Chain 3
1	0	0

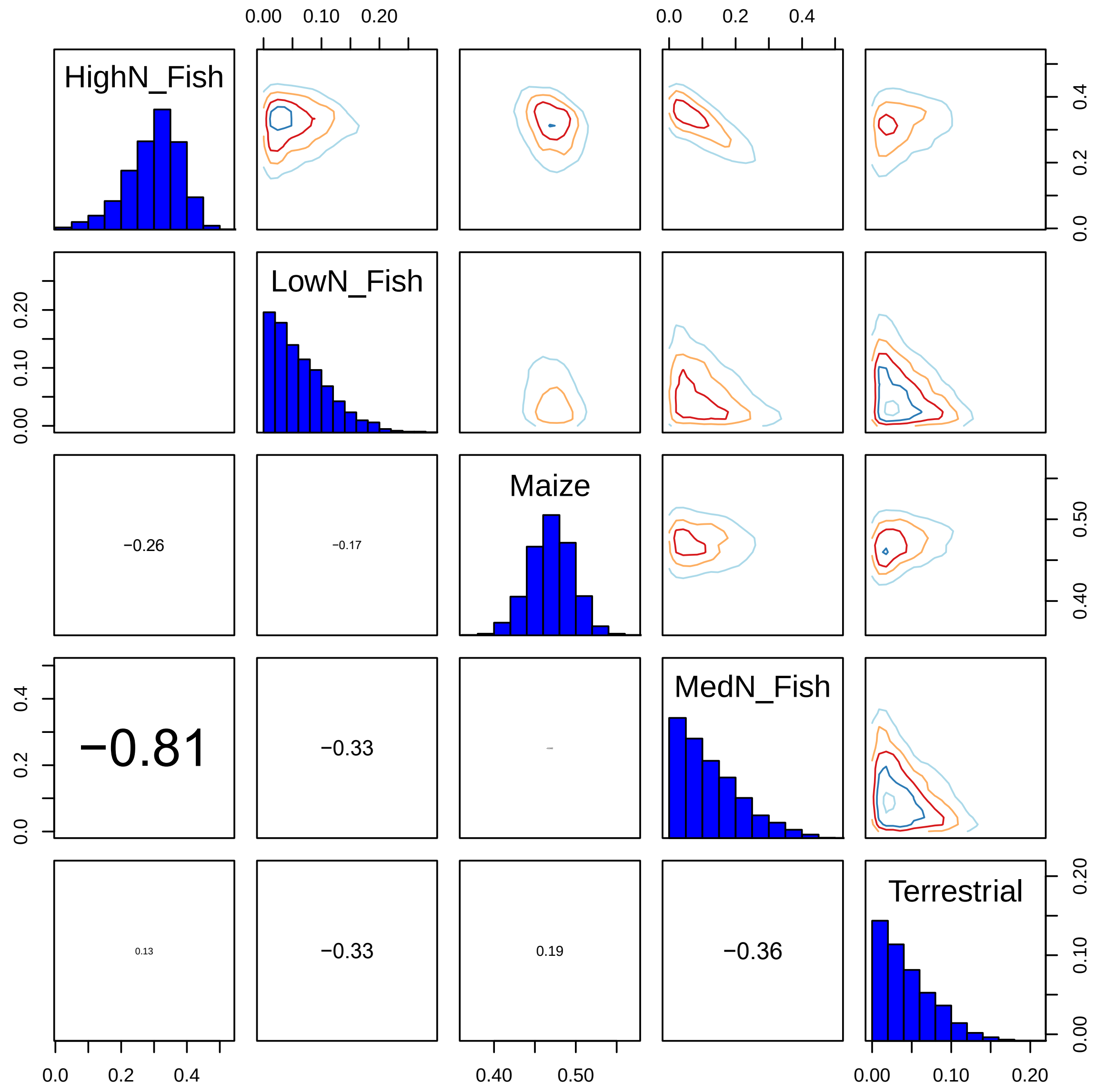

17th Century Dog Model 1 Diagnostics (n=41)

Gelman Diagnostic (variables=47)

>1.01	>1.05	>1.10
0	0	0

Geweke diagnostic (variables=47)

Chain 1	Chain 2	Chain 3
2	0	1

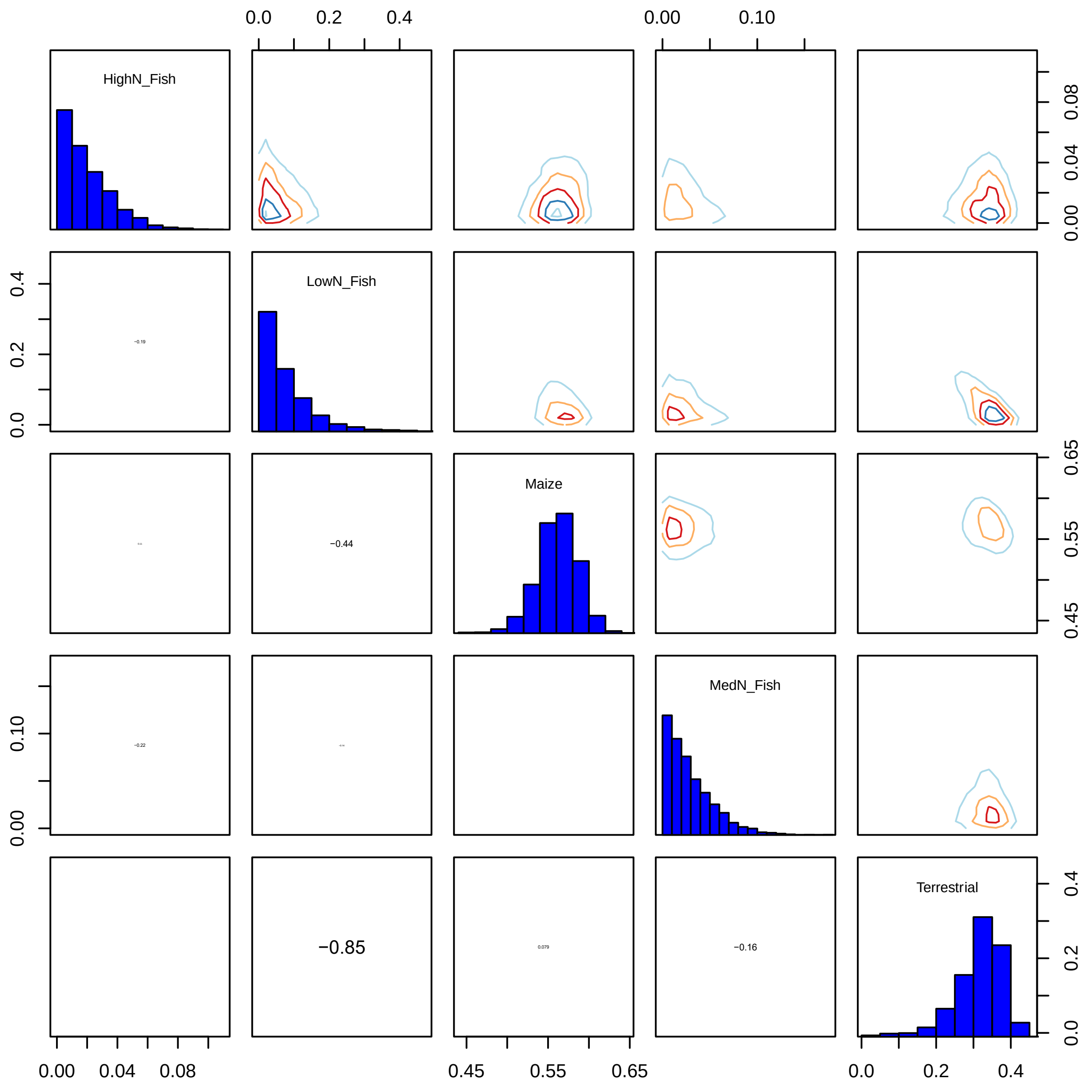

17th Century Dog Model 2 Diagnostics (n=41)

Gelman Diagnostic (variables=48)

>1.01	>1.05	>1.10
0	0	0

Geweke diagnostic (variables=48)

Chain 1	Chain 2	Chain 3
0	0	2

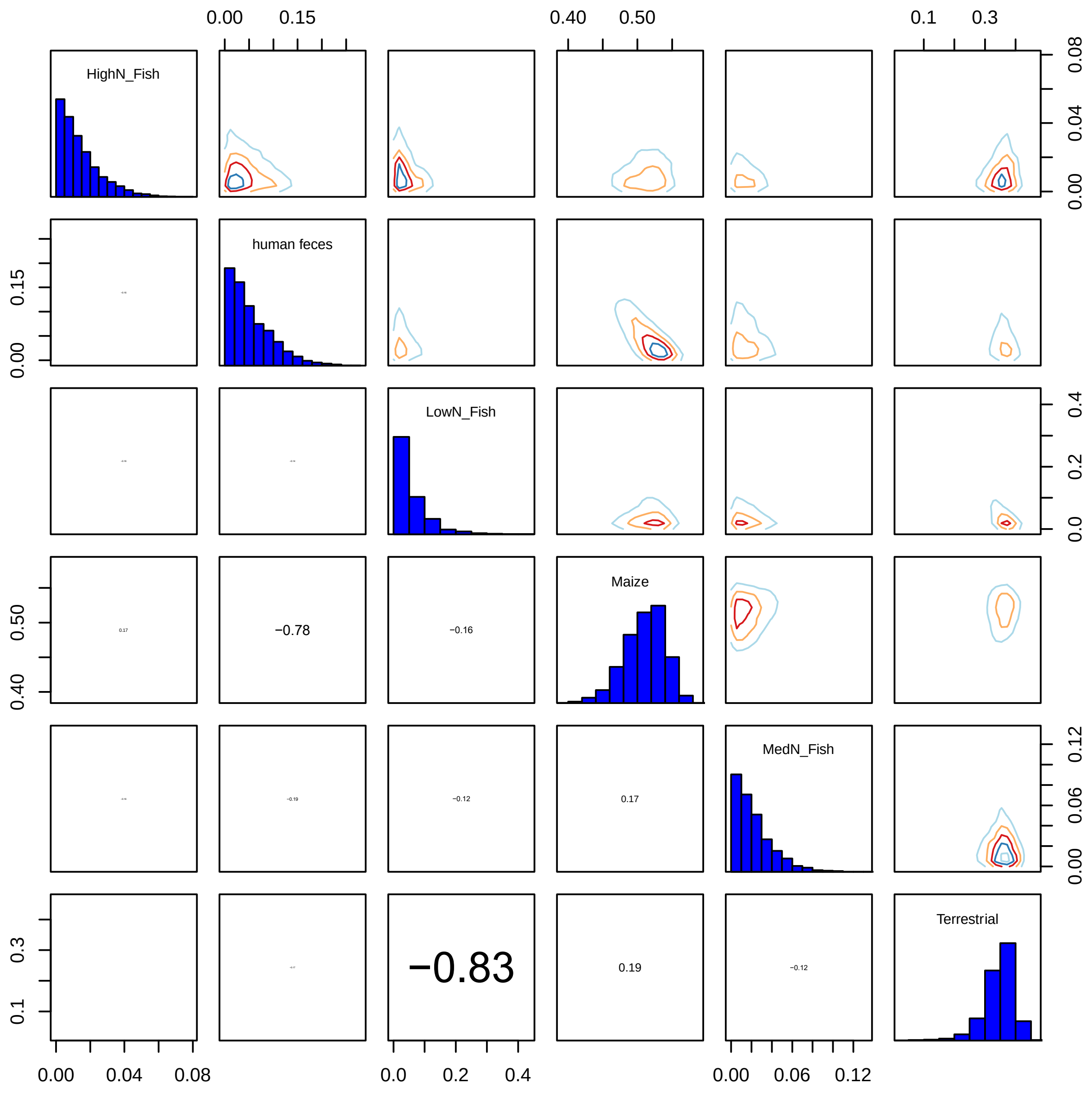

17th Century Dog Model 3 Diagnostics (n=41)

Gelman Diagnostic (variables=49)

>1.01	>1.05	>1.10
0	0	0

Geweke diagnostic (variables=49)

Chain 1	Chain 2	Chain 3
1	0	1

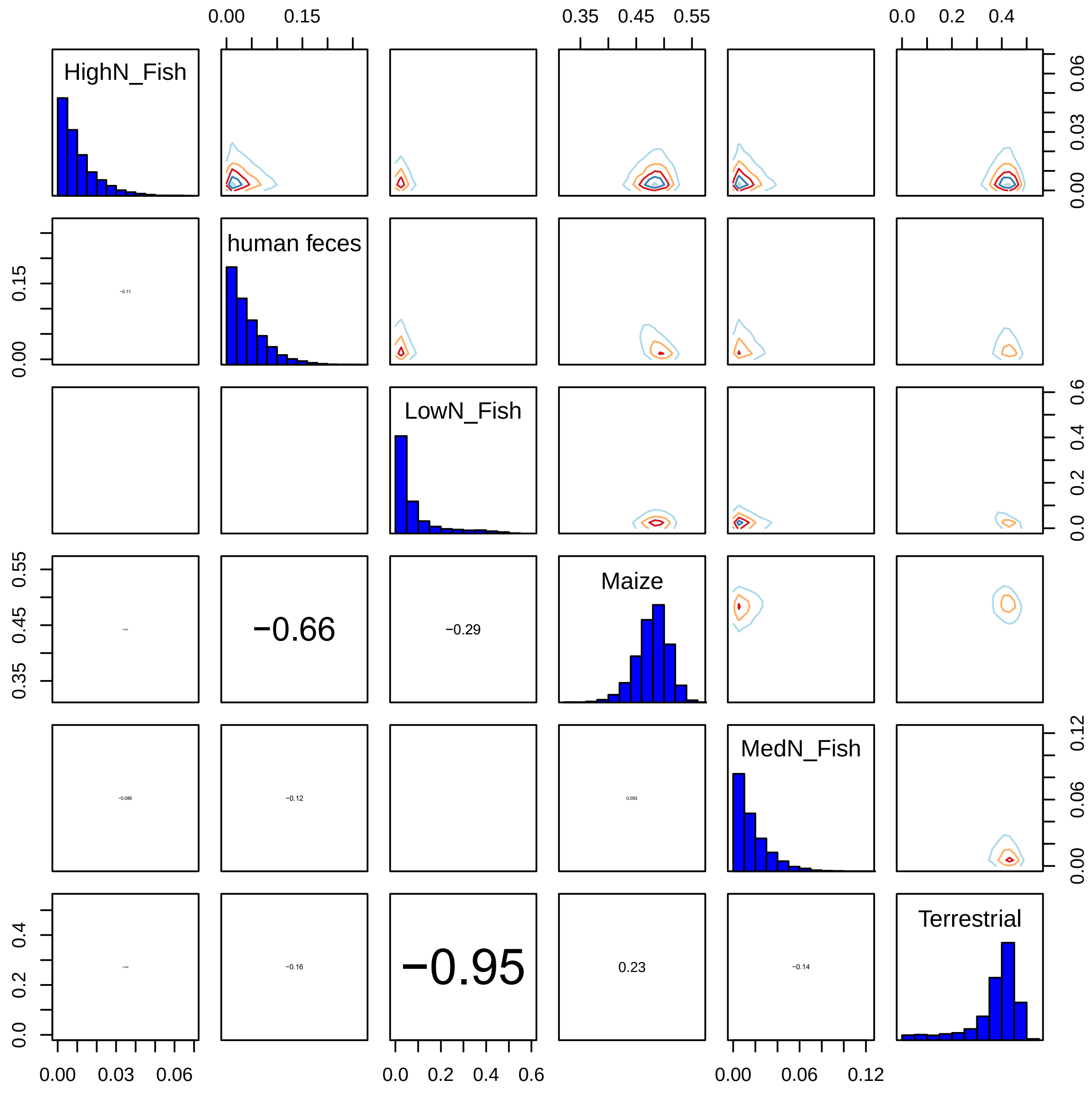

Fairty Ossuary Human Model Diagnostics (n=8)

Gelman Diagnostic (variables=14)

>1.01	>1.05	>1.10
0	0	0

Geweke diagnostic (variables=14)

Chain 1	Chain 2	Chain 3
1	0	0

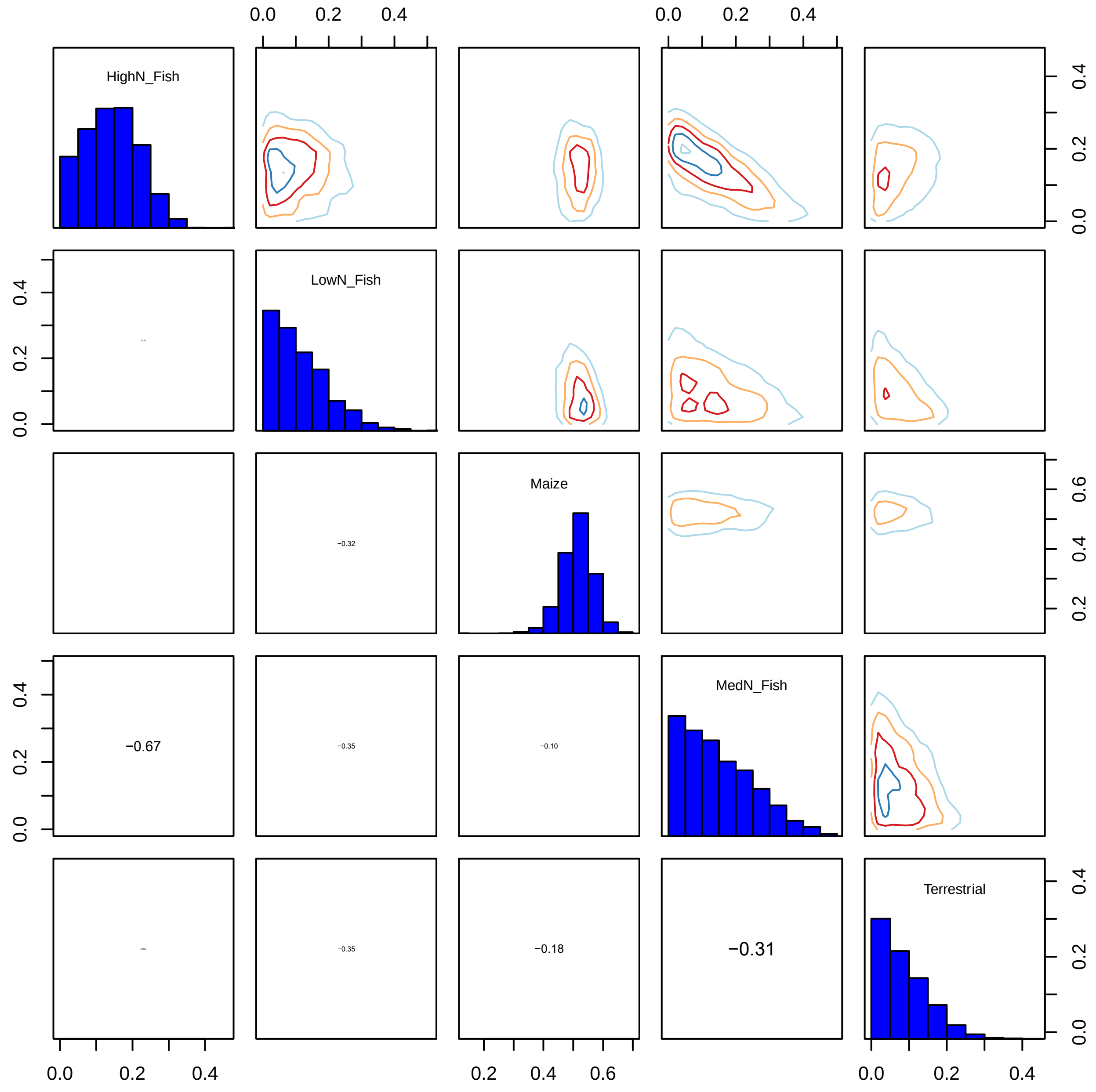

Robb Village Dog Model 1 Diagnostics (n=9)

Gelman Diagnostic (variables=14)

>1.01	>1.05	>1.10
0	0	0

Geweke diagnostic (variables=14)

Chain 1	Chain 2	Chain 3
0	0	0

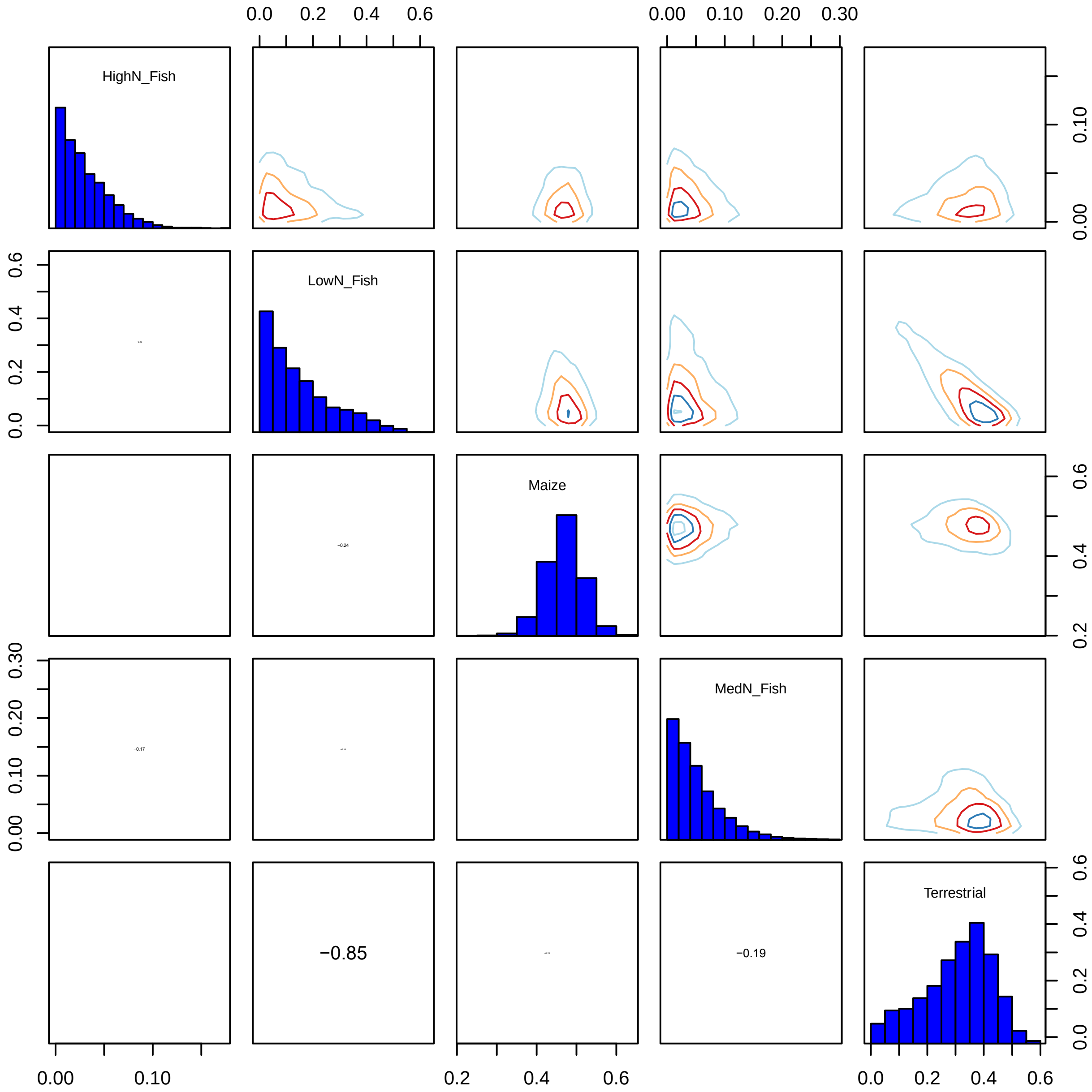

Robb Village Dog Model 2 Diagnostic (n=9)

Gelman Diagnostic (variables=16)

>1.01	>1.05	>1.10
0	0	0

Geweke diagnostic (variables=19)

Chain 1	Chain 2	Chain 3
0	0	0

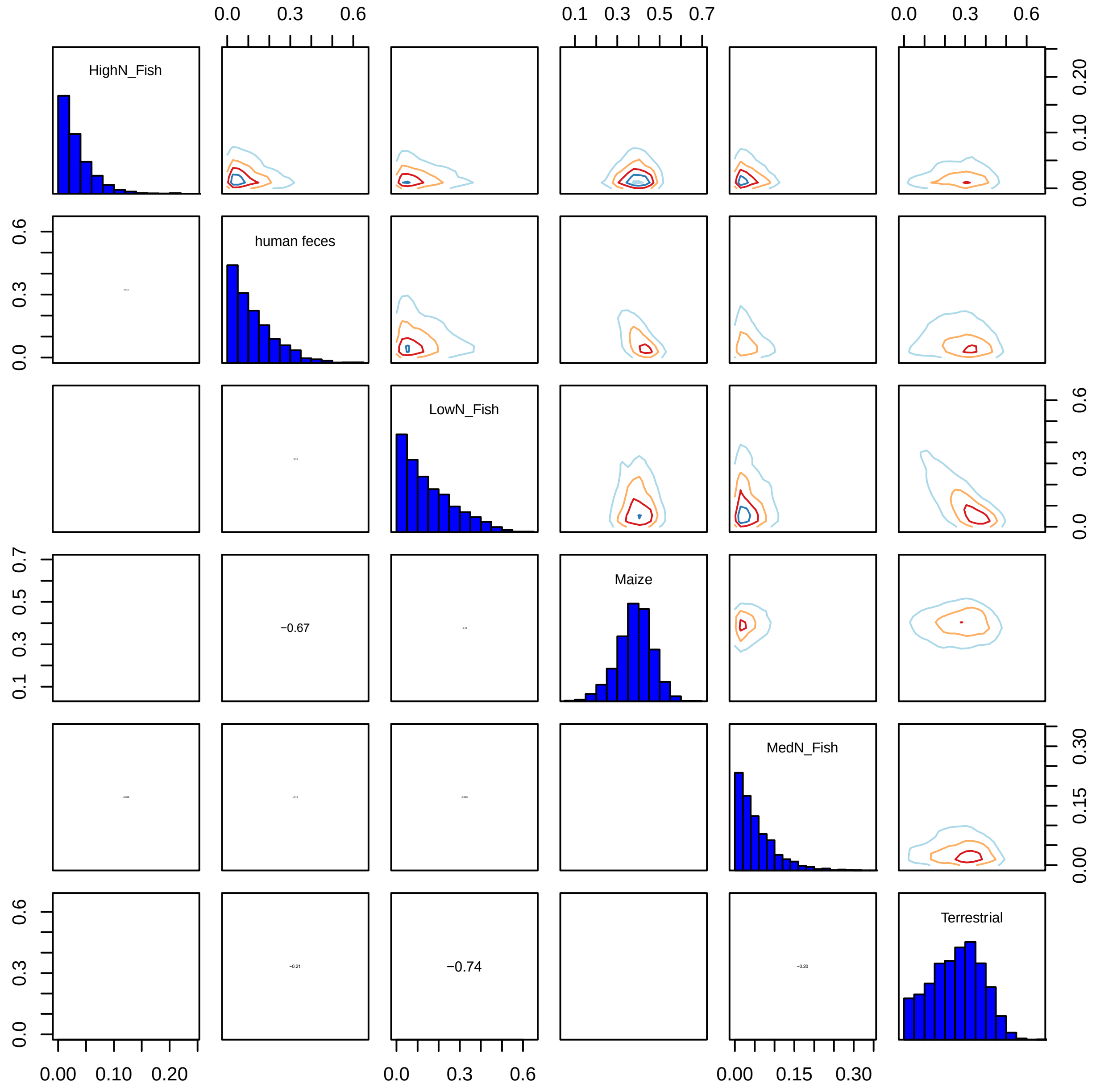

Robb Village Dog Model 3 Diagnostics (n=9)

Gelman Diagnostic (variables=17)

>1.01	>1.05	>1.10
0	0	0

Geweke diagnostic (variables=17)

Chain 1	Chain 2	Chain 3
0	0	0

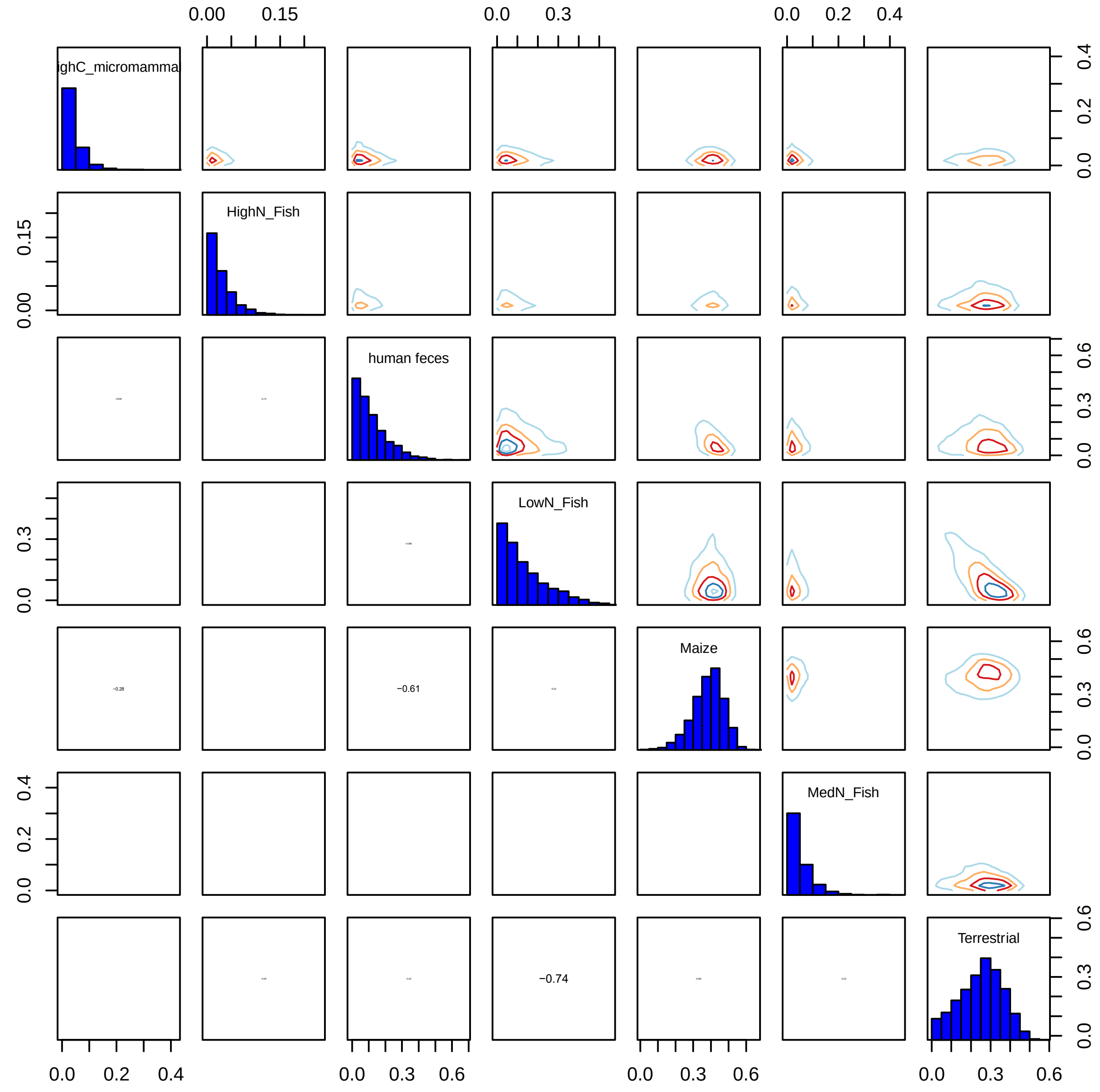

Kleinberg Ossuary Human Model Diagnostics (n=12)

Gelman Diagnostic (variables=17)

>1.01	>1.05	>1.10
0	0	0

Geweke diagnostic (variables=17)

Chain 1	Chain 2	Chain 3
0	0	0

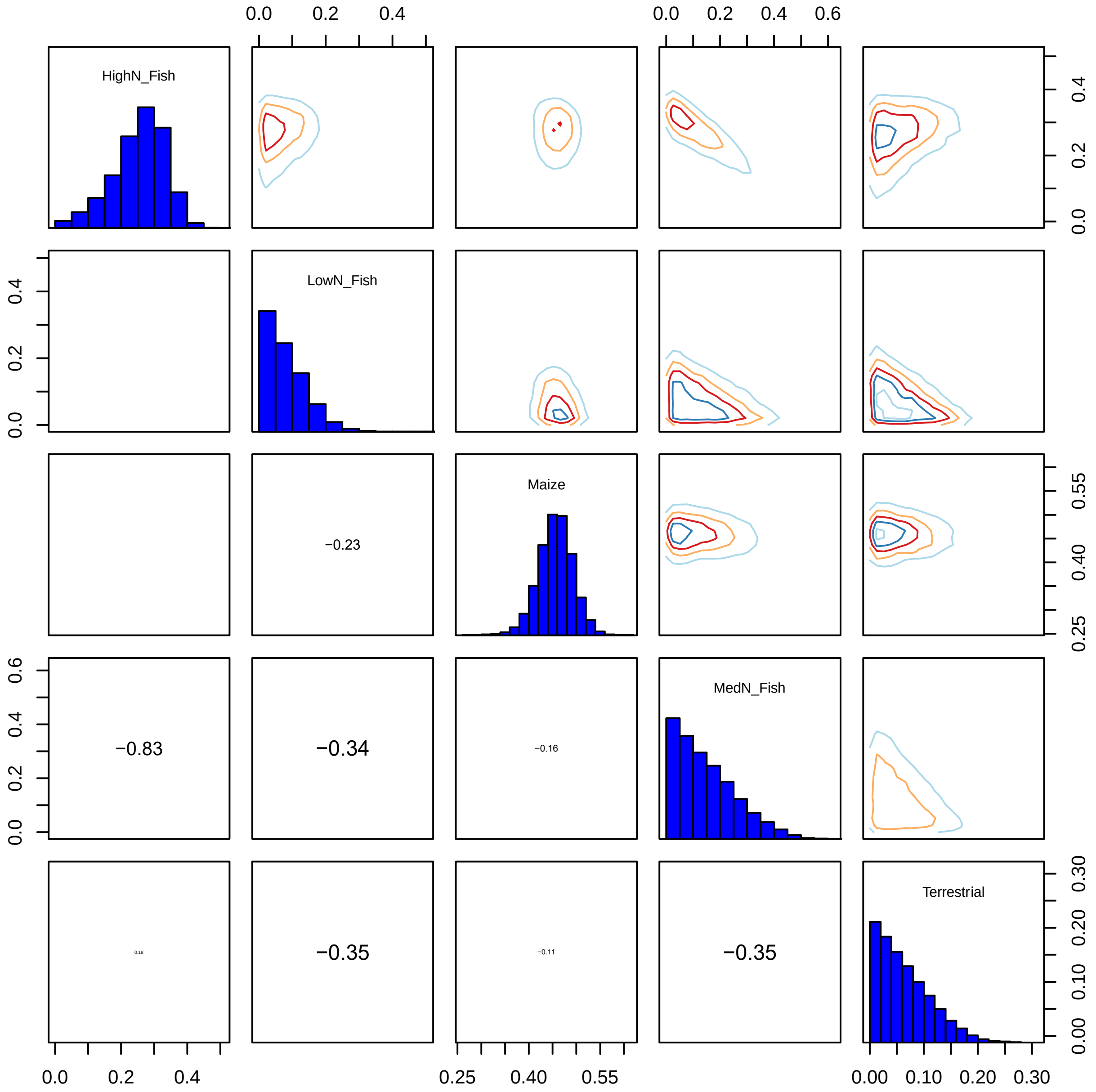

Seed-Barker Village Dog Model 1 Diagnostics (n=5)

Gelman Diagnostic (variables=11)

>1.01	>1.05	>1.10
0	0	0

Geweke diagnostic (variables=11)

Chain 1	Chain 2	Chain 3
0	0	0

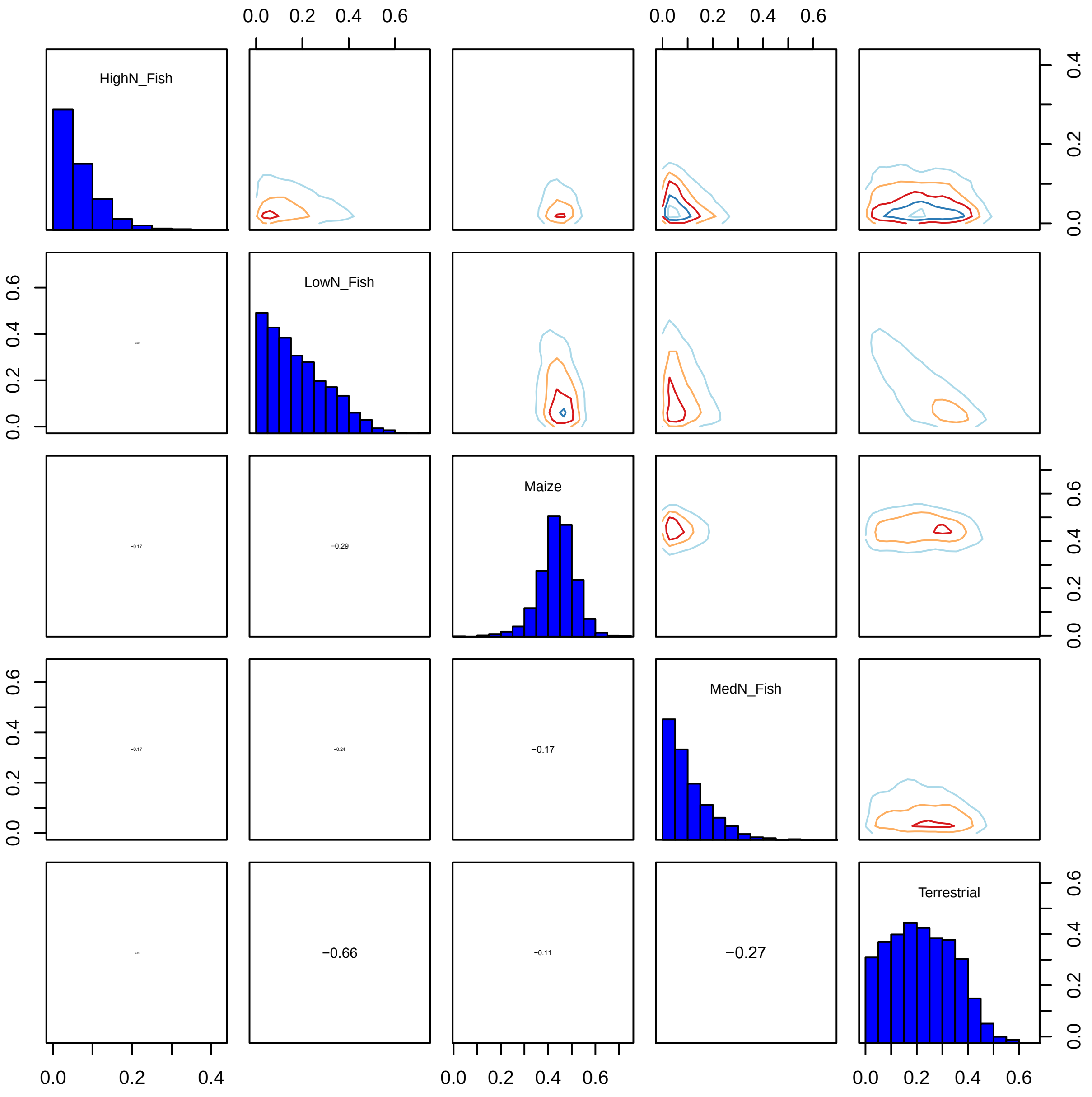

Seed-Barker Village Dog Model 2 Diagnostics (n=5)

Gelman Diagnostic (variables=12)

>1.01	>1.05	>1.10
0	0	0

Geweke diagnostic (variables=12)

Chain 1	Chain 2	Chain 3
0	0	0

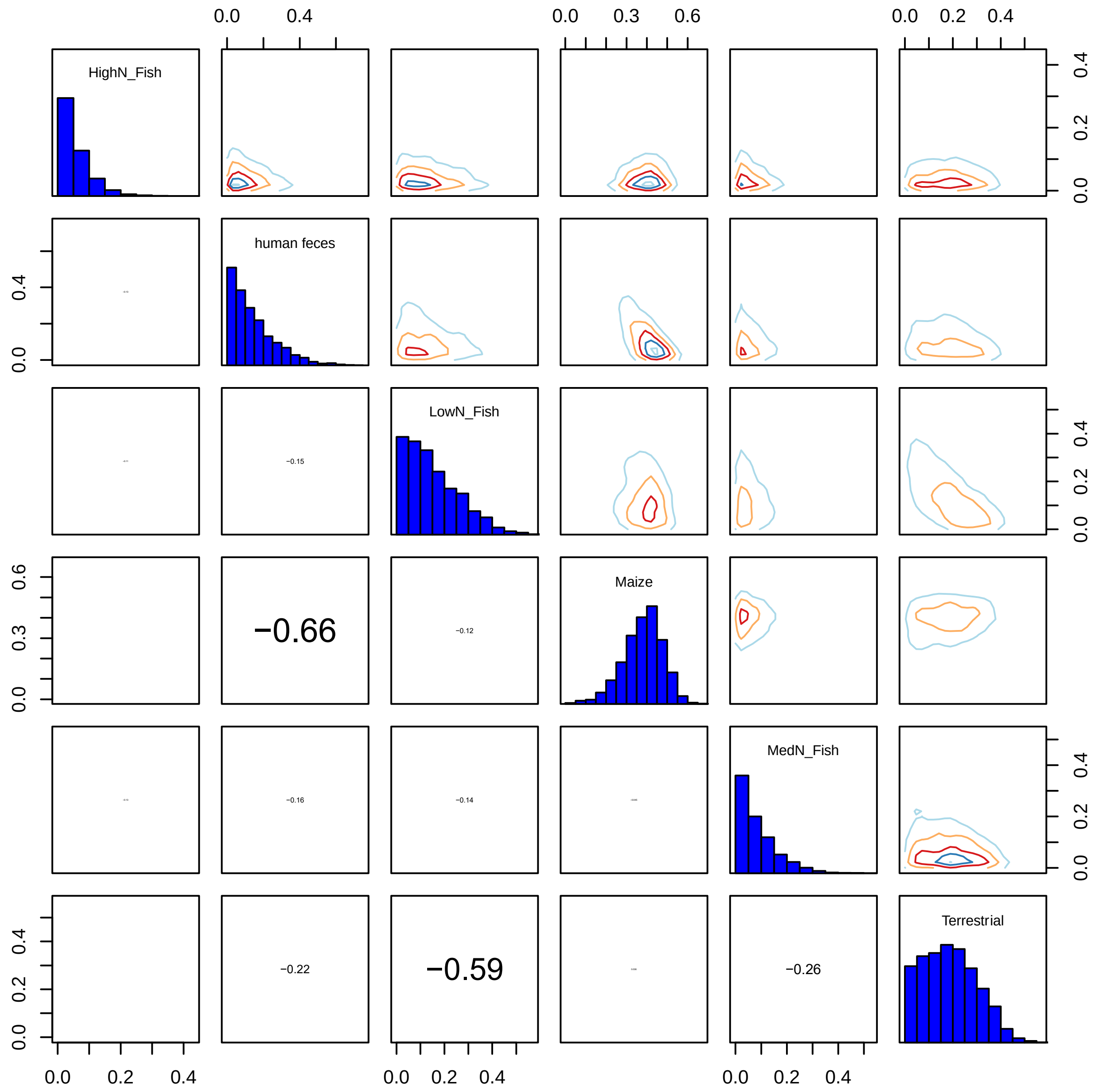

Seed-Barker Village Dog Model 3 Diagnostics (n=5)

Gelman Diagnostic (variables=13)

>1.01	>1.05	>1.10
0	0	0

Geweke diagnostic (variables=13)

Chain 1	Chain 2	Chain 3
0	0	0

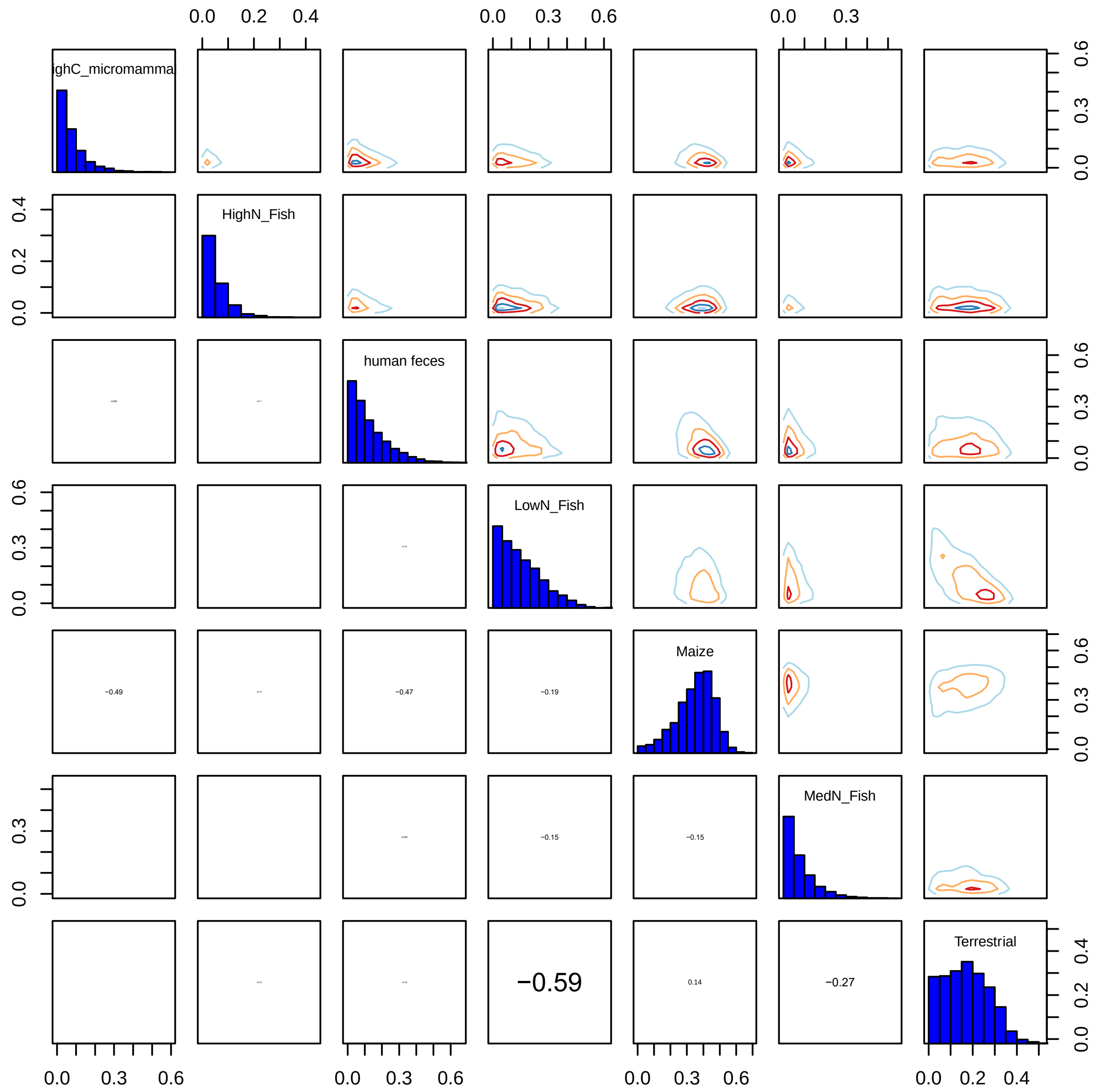

Ball Village Human Model Disgnostics (n=6)

Gelman Diagnostic (variables=12)

>1.01	>1.05	>1.10
1	0	0

Geweke diagnostic (variables=12)

Chain 1	Chain 2	Chain 3
0	0	0

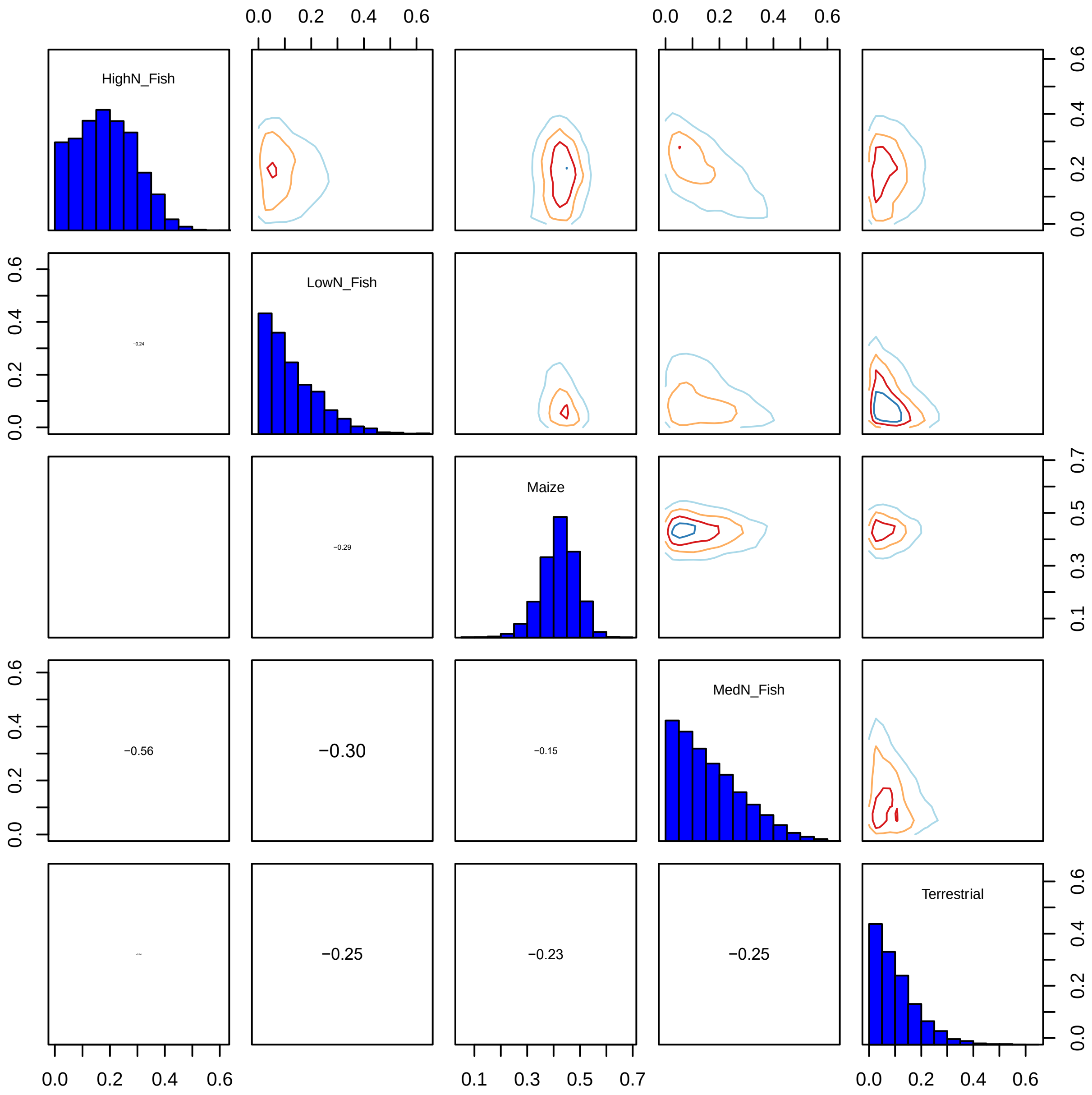

Ball Village Dog Model 1 Disgnostics (n=12)

Gelman Diagnostic (variables=18)

>1.01	>1.05	>1.10
1	0	0

Geweke diagnostic (variables=18)

Chain 1	Chain 2	Chain 3
0	1	0

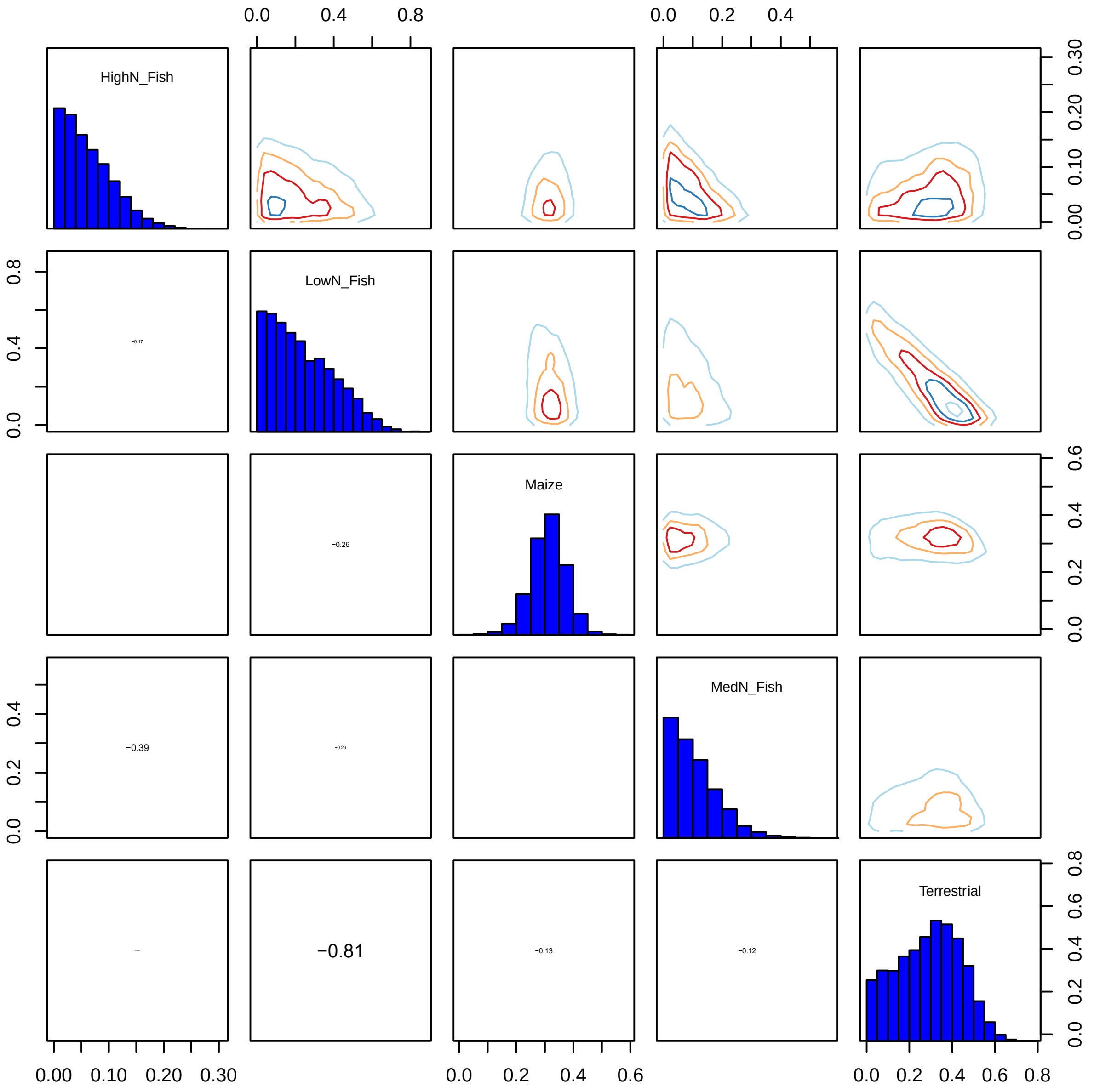

Ball Village Dog Model 2 Diagnostics (n=12)

Gelman Diagnostic (variables=19)

>1.01	>1.05	>1.10
0	0	0

Geweke diagnostic (variables=19)

Chain 1	Chain 2	Chain 3
1	0	0

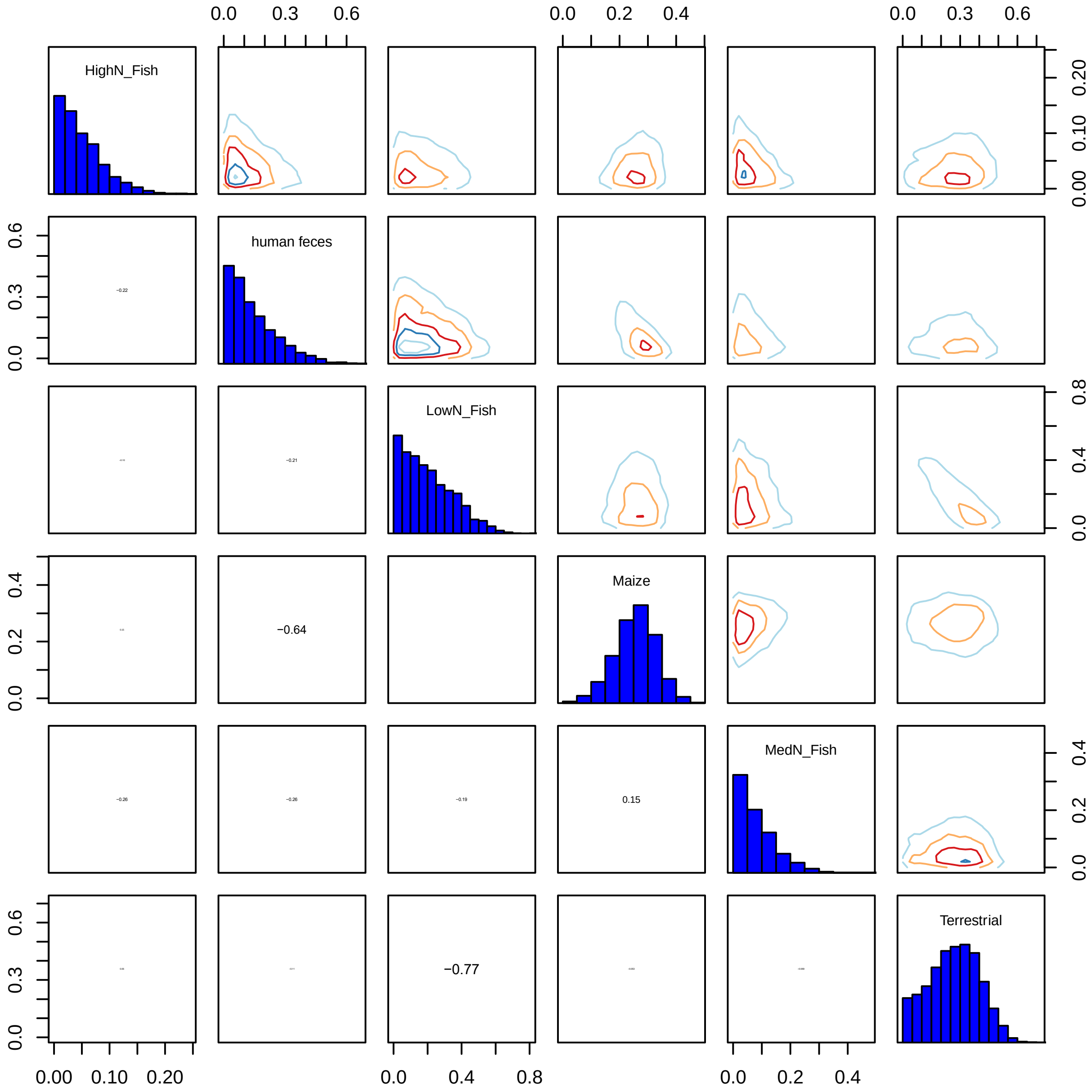

Ball Village Dog Model 3 Diagnostics (n=12)

Gelman Diagnostic (variables=20)

>1.01	>1.05	>1.10
0	0	0

Geweke diagnostic (variables=20)

Chain 1	Chain 2	Chain 3
0	1	0

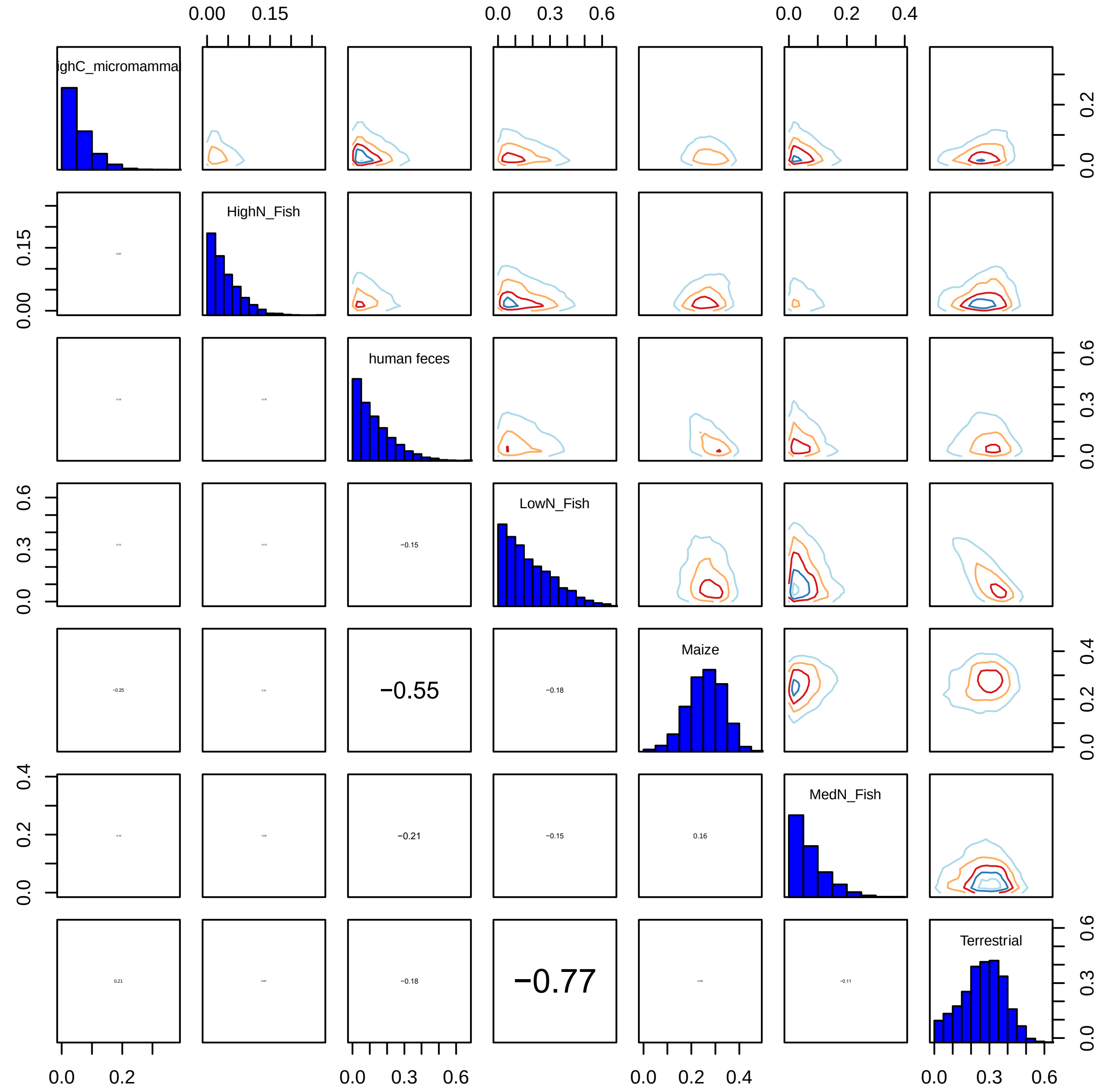

Kelly Campbell Village Human Model Diagnostics (n=6)

Gelman Diagnostic (variables=12)

>1.01	>1.05	>1.10
0	0	0

Geweke diagnostic (variables=12)

Chain 1	Chain 2	Chain 3
0	0	0

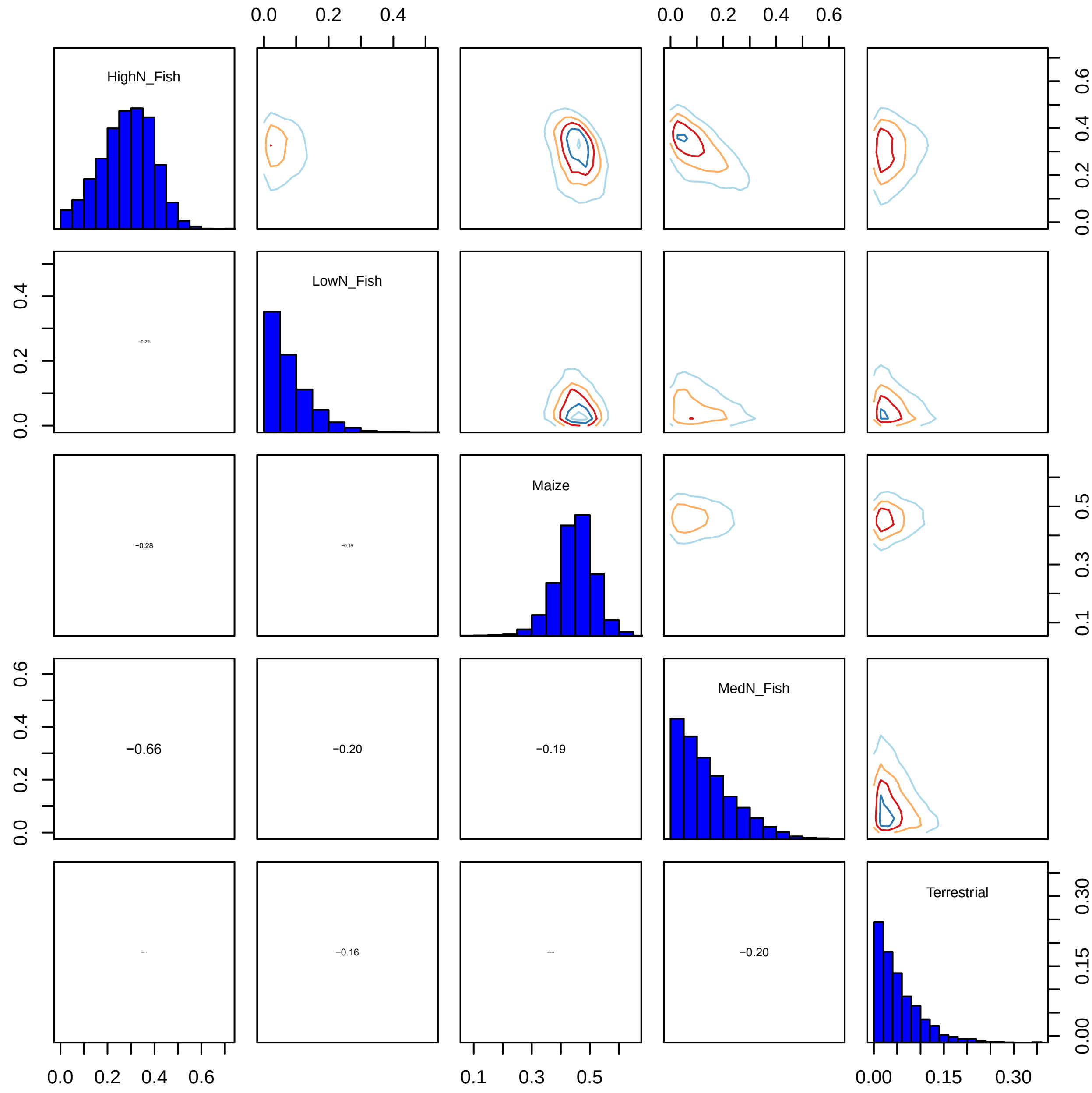

Kelly Campbell Village Dog Model 1 Diagnostics (n=8)

Gelman Diagnostic (variables=14)

>1.01	>1.05	>1.10
0	0	0

Geweke diagnostic (variables=14)

Chain 1	Chain 2	Chain 3
0	0	0

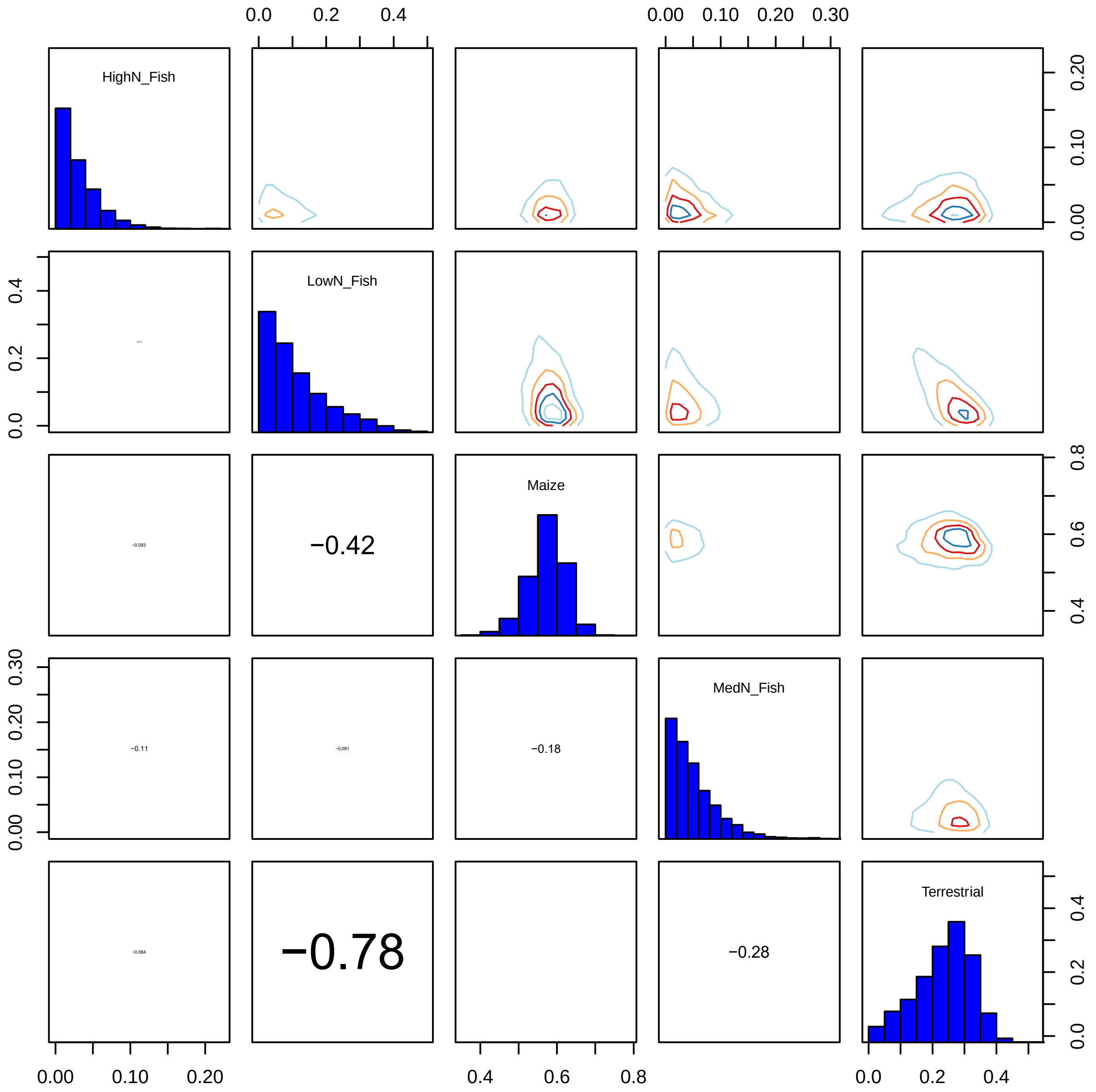

Kelly Campbell Village Dog Model 2 Diagnostics (n=8)

Gelman Diagnostic (variables=15)

>1.01	>1.05	>1.10
1	0	0

Geweke diagnostic (variables=15)

Chain 1	Chain 2	Chain 3
0	0	0

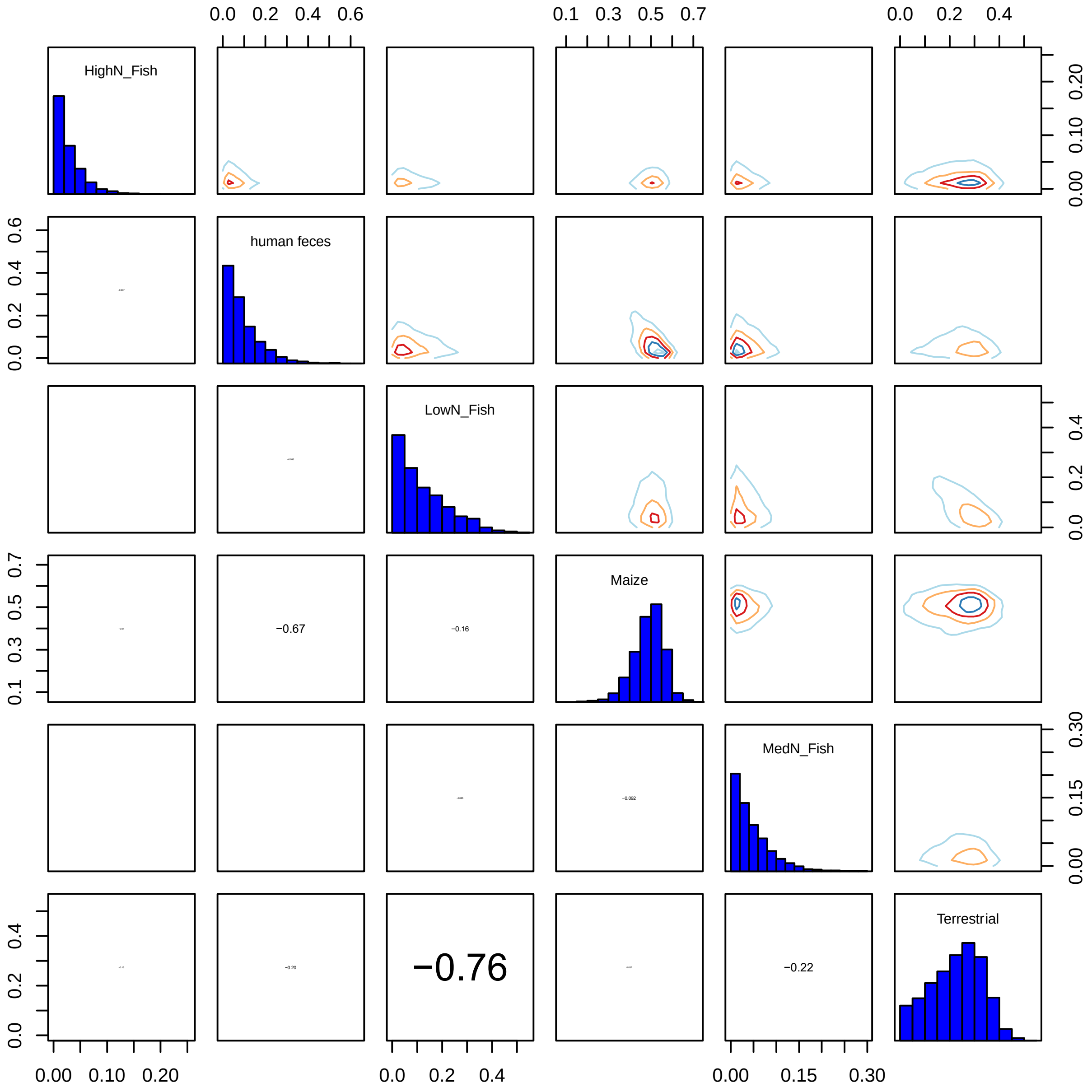

Kelly Campbell Village Dog Model 2 Diagnostics (n=8)

Gelman Diagnostic (variables=16)

>1.01	>1.05	>1.10
0	0	0

Geweke diagnostic (variables=16)

Chain 1	Chain 2	Chain 3
0	0	0

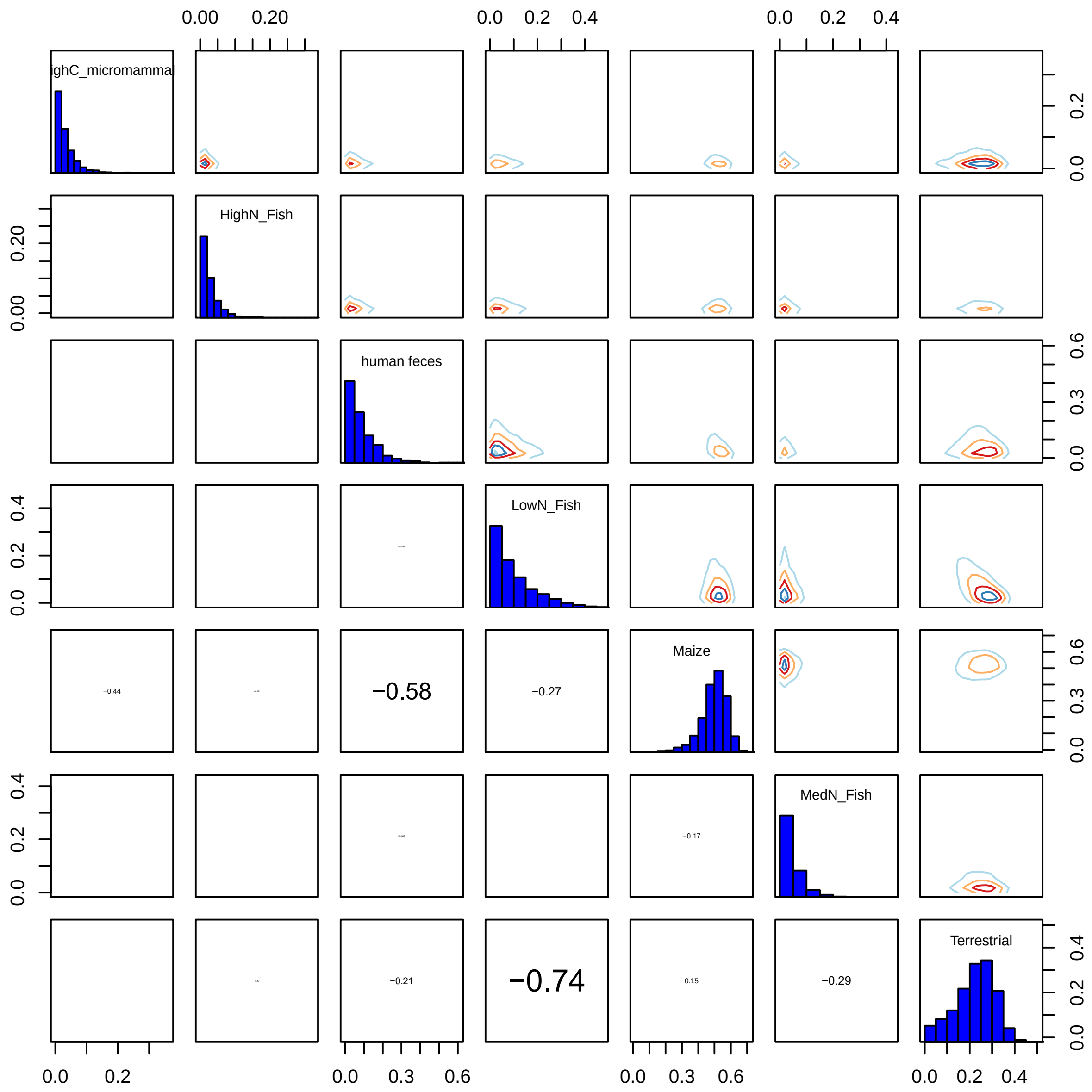

Ossossoané Ossuary Human Model Diagnostics (n=5)

Gelman Diagnostic (variables=11)

>1.01	>1.05	>1.10
0	0	0

Geweke diagnostic (variables=11)

Chain 1	Chain 2	Chain 3
1	0	0

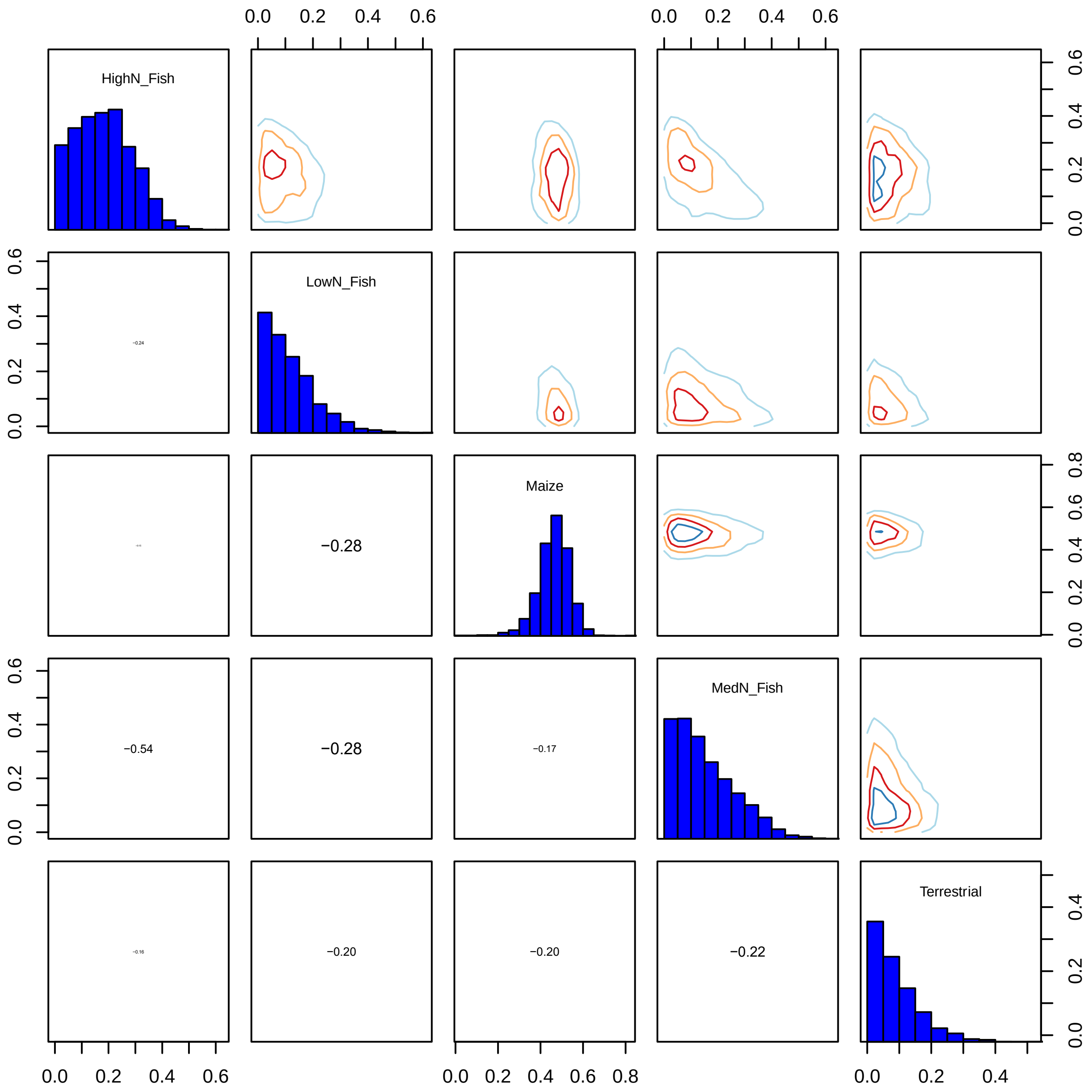

Ossossoané Village Dog Model 1 Diagnostics (n=11)

Gelman Diagnostic (variables=17)

>1.01	>1.05	>1.10
0	0	0

Geweke diagnostic (variables=17)

Chain 1	Chain 2	Chain 3
0	0	0

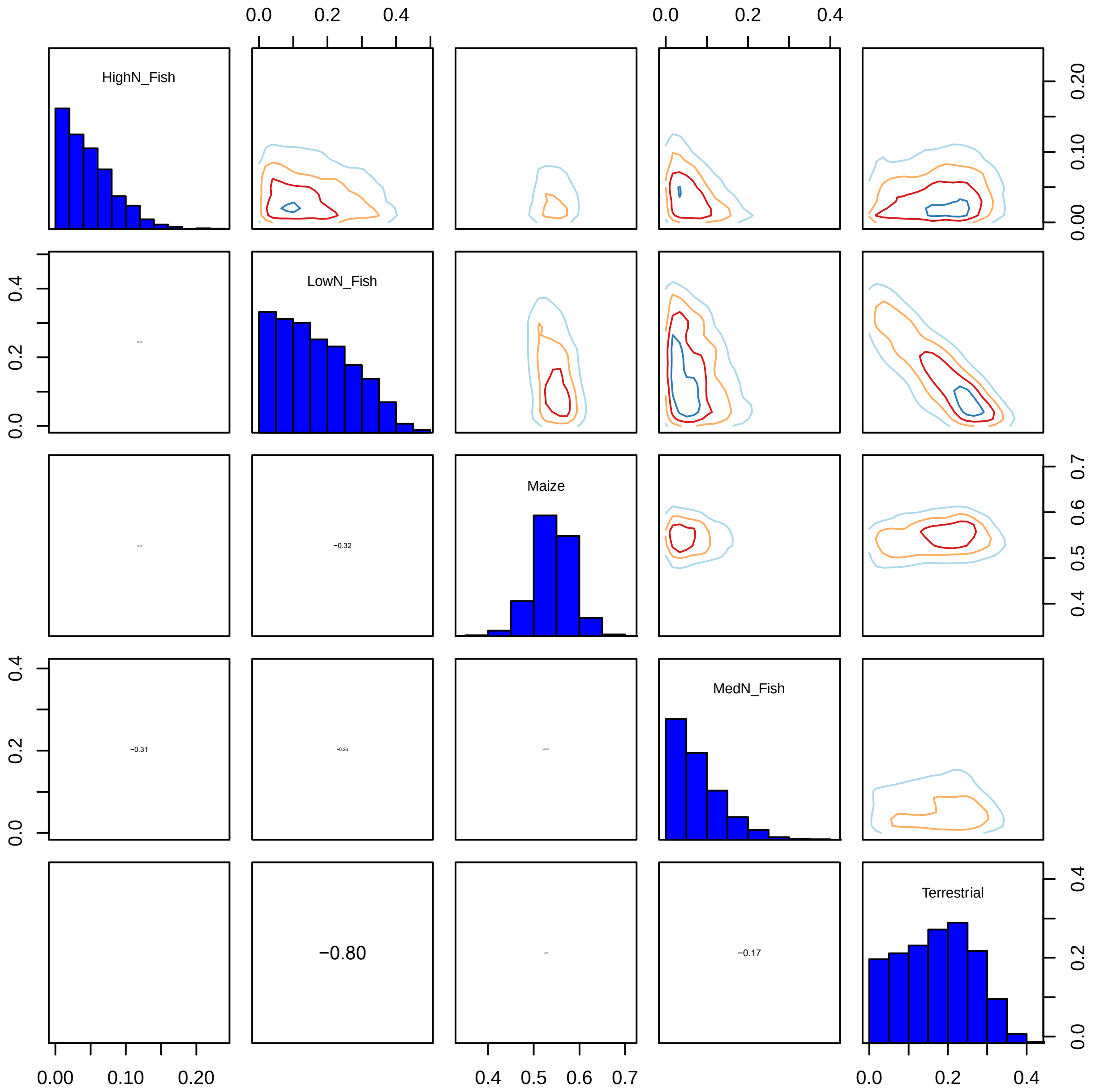

Ossossoané Village Dog Model 2 Diagnostics (n=11)

Gelman Diagnostic (variables=18)

>1.01	>1.05	>1.10
0	0	0

Geweke diagnostic variables=18)

Chain 1	Chain 2	Chain 3
0	1	0

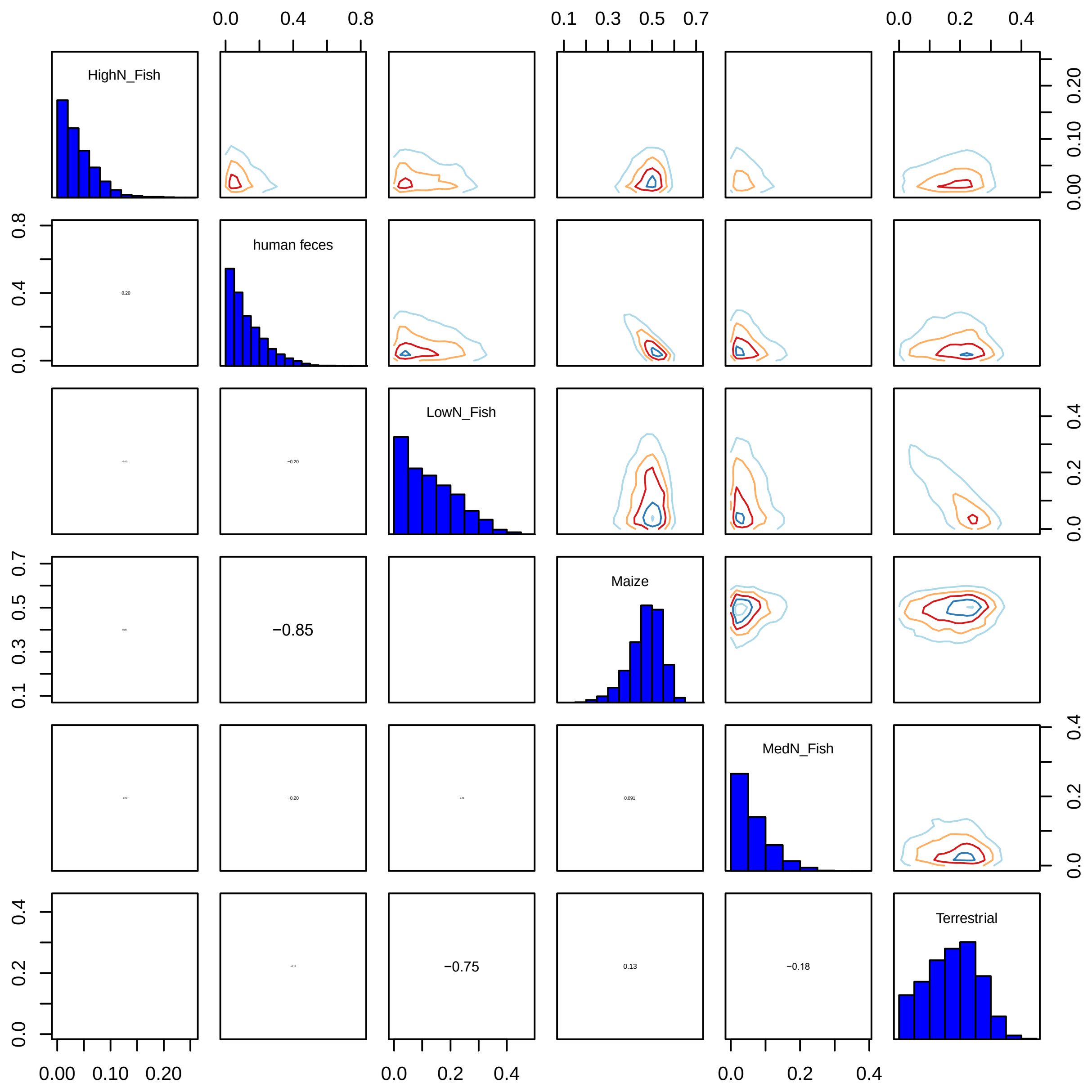

Ossossoané Village Dog Model 3 Diagnostics (n=11)

Gelman Diagnostic (variables=19)

>1.01	>1.05	>1.10
0	0	0

Geweke diagnostic (variables=19)

Chain 1	Chain 2	Chain 3
0	0	0

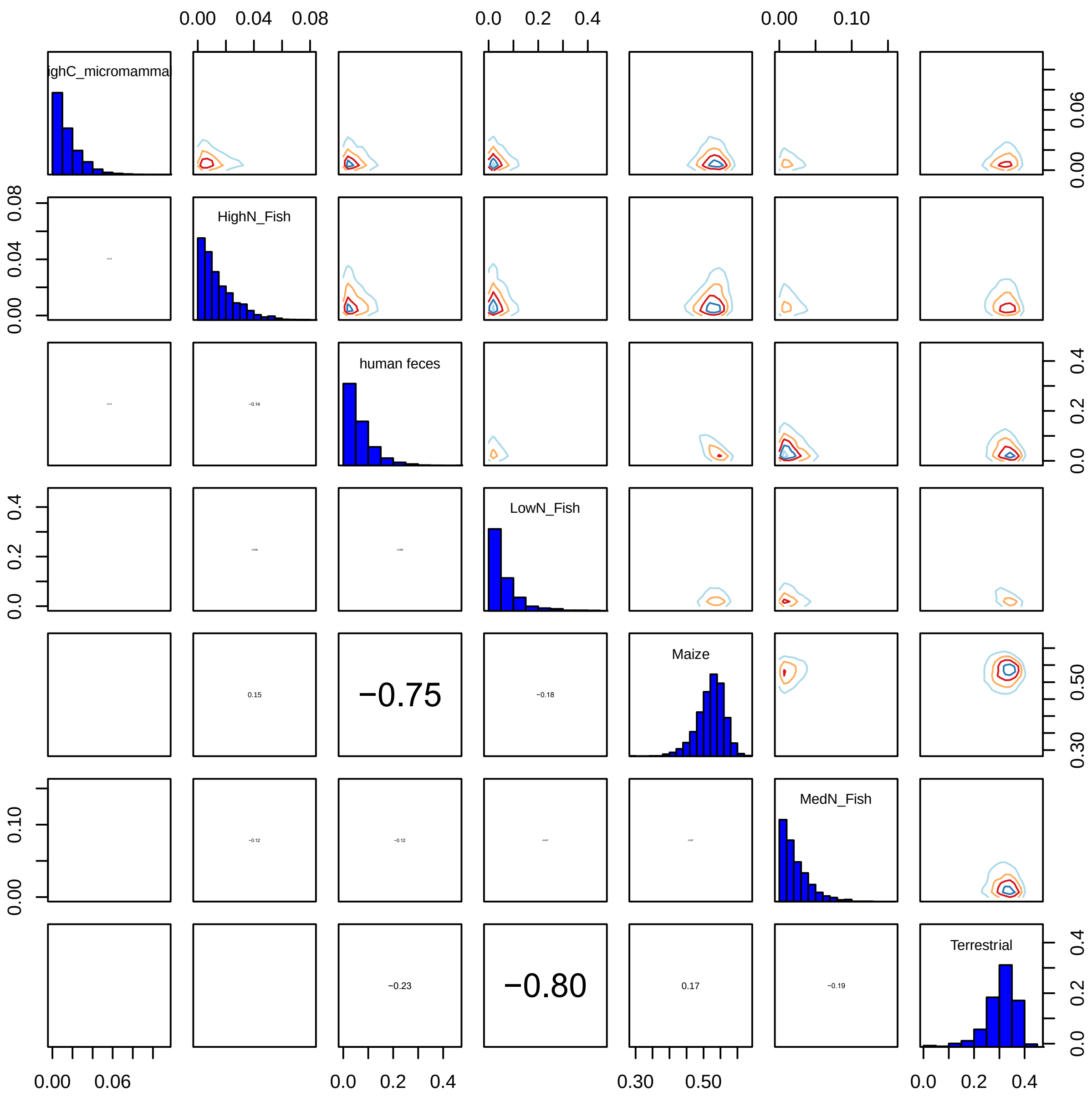

Supplement: Supplementary file 1 — Supplementary Information 1. [file 41598_2023_34216_MOESM1_ESM.pdf]
