# Supplementary material for: Human and dog Bayesian dietary mixing models using bone collagen stable isotope ratios from ancestral Iroquoian sites in southern Ontario
Source: Sci Rep. 2023 May 3;13:7177. doi: 10.1038/s41598-023-34216-6 (PMC10156743; doi:10.1038/s41598-023-34216-6)
Supplement: Supplementary file 2 — Supplementary Information 2. [file 41598_2023_34216_MOESM2_ESM.pdf]

Human and Dog Bayesian Dietary Mixing Models using Bone Collagen Stable Isotope Ratios from Ancestral Iroquoian Sites in Southern Ontario

Supplementary Data S2
Data used in Bayesian dietary mixing models

John P. Hart and Robert S. Feranec
Research and Collections Division
New York State Museum
Albany, New York 12230

Contents

Example MixSIAR model code, p. 2.
S2 Table 1. Dog collagen isotope ratios used in Bayesian dietary mixing models. pp.4-6
S2 Table 2. Human collagen and feces estimate isotope ratios used in Bayesian dietary mixing models. pp. 7-9
S2 Table 3. Terrestrial prey collagen isotope ratios used in Bayesian dietary mixing models. pp. 9-22
S2 Table 4. Fish collagen isotope ratios used in Bayesian dietary mixing models. pp. 22-27
S2 Table 5. Micromammal collagen isotope ratios used in Bayesian dietary mixing models. p. 28
S2 Table 6. Source isotope means, standard deviations and concentrations used in models. p. 29
S2 Table 7. C and N concentration calculations. p. 29
S2 Table 8. Trophic enrichment factors used in models. p. 29
References Cited. p. 30

```
# source$data_type: means
# source$by_factor: NA
# random effects: 0
# fixed effects: 0
# nested factors:
# factors:
# continuous effects: 0
# error structure: Residual only
# source$conc_dep: TRUE

model{
  for(src in 1:n.sources){
    for(iso in 1:n.iso){
      src_mu[src,iso] ~ dnorm(MU_array[src,iso], n_array[src]/SIG2_array[src,iso]);  # Eqn 3.8 but with
precision instead of variance
      tmp.X[src,iso] ~ dchisqr(n_array[src]);
      src_tau[src,iso] <- tmp.X[src,iso]/(SIG2_array[src,iso]*(n_array[src] - 1));   # Eqn 3.9, following the
simulation on p.580
    }
  }

    # draw p.global (global proportion means) from an uninformative Dirichlet,
    # then ilr.global is the ILR-transform of p.global
    p.global[1:n.sources] ~ ddirch(alpha[1:n.sources]);
    for(src in 1:(n.sources-1)){
      gmean[src] <- prod(p.global[1:src])^(1/src);
      ilr.global[src] <- sqrt(src/(src+1))*log(gmean[src]/p.global[src+1]); # page 296, Egozcue 2003
    }

   # DON'T generate individual deviates from the global/region/pack mean (but keep same model structure)
   for(i in 1:N) {
      for(src in 1:(n.sources-1)) {
        ilr.ind[i,src] <- 0;
        ilr.tot[i,src] <- ilr.global[src] + ilr.ind[i,src]; # add all effects together for each individual (in ilr-space)
      }
   }

   # Inverse ILR math (equation 24, page 294, Egozcue 2003)
   for(i in 1:N){
      for(j in 1:(n.sources-1)){
        cross[i,,j] <- (e[,j]^ilr.tot[i,j])/sum(e[,j]^ilr.tot[i,j]);
      }
      for(src in 1:n.sources){
        tmp.p[i,src] <- prod(cross[i,src,]);
      }
      for(src in 1:n.sources){
```

```
      p.ind[i,src] <- tmp.p[i,src]/sum(tmp.p[i,]);
    }
  }

  for(src in 1:n.sources) {
    for(i in 1:N){
      # these are weights for variances
      p2[i,src] <- p.ind[i,src]*p.ind[i,src];
    }
  }


  # for each isotope and population, calculate the predicted mixtures
  for(iso in 1:n.iso) {
    for(i in 1:N) {

      mix.mu[iso,i] <- (inprod(src_mu[,iso],(p.ind[i,]*conc[,iso])) +
inprod(frac_mu[,iso],(p.ind[i,]*conc[,iso]))) / inprod(p.ind[i,],conc[,iso]);
    }
  }

  # Mixture covariance prior (residual error only model)
  Sigma ~ dwish(I,n.iso+1);

  # Likelihood
  for(i in 1:N) {
    X_iso[i,] ~ dmnorm(mix.mu[,i], Sigma);
    loglik[i] <- logdensity.mnorm(X_iso[i,], mix.mu[,i], Sigma);
  }
} # end model
```

S2 Table 1. Dog collagen isotope ratios used in Bayesian dietary mixing models.

Site	Century	Sample ID	Sample Description	$\delta^{13}C$	$\delta^{15}N$	Source
Slack-Caswell	14th	Sla-018 DUP		-10.6	9.8	Guiry et al. 2021
Slack-Caswell	14th	Sla-019	phalanx	-11.4	9.4	Guiry et al. 2021
New	14th	BC 6765	phalanx, proximal	-14.0	9.5	Guiry et al. 2021
New	14th	BC 6764	tooth, molar, lower 1	-11.0	11.3	Guiry et al. 2021
Robb	14th	Robb 01	radius	-12.7	10.3	Glencross et al 2022
Robb	14th	Robb 02	lumbar vertibra	-10.6	9.8	Glencross et al 2022
Robb	14th	Robb 04	ulna	-13.6	10.0	Glencross et al 2022
Robb	14th	Robb 07	temporal	-12.2	10.6	Glencross et al 2022
Robb	14th	Robb 08	ulna	-12.1	10.1	Glencross et al 2022
Robb	14th	Robb 09	humerus	-17.7	9.1	Glencross et al 2022
Robb	14th	Robb 11	cervical vertibra	-12.1	10.0	Glencross et al 2022
Robb	14th	Robb 12	ulna	-10.9	9.5	Glencross et al 2022
Robb	14th	Robb 13	humerus	-11.7	9.8	Glencross et al 2022
Bogle II	15th	Bog-016	mandible	-13.7	9.4	Morris 2017
Carson	15th	CAR01.1	left humerus	-11.5	9.6	Booth 2014
Carson	15th	CAR15.1	metacarpal	-13.5	9.0	Booth 2014
Carson	15th	CAR16.1	metacarpal	-13.9	8.8	Booth 2014
Carson	15th	CAR25.1	right mandible	-10.9	10.1	Booth 2014
Carson	15th	CAR30.1	metacarpal	-11.7	8.8	Booth 2014
Carson	15th	CAR37.1	metatarsal	-12.2	9.0	Booth 2014
Crawford Lake	15th	Crf-054	axis	-11.9	9.5	Morris 2017
Pipeline	15th	Pip-(1)-138B Dup	radius, left complete	-10.6	9.8	Booth 2014
Pipeline	15th	Pip-(1)-180+	ulna, left complete	-11.4	10.9	Booth 2014
Pipeline	15th	Pip(2)-018	atlas	-12.5	9.1	Booth 2014
Pipeline	15th	Pip(2)-044	scapula, left	-10.9	10.3	Booth 2014
Pipeline	15th	Pip(2)-087	unla, right proximal	-12.2	10.1	Booth 2014
Pipeline	15th	PIP01.1	atlas	-12.5	9.1	Booth 2014
Pipeline	15th	PIP02.1	atlas	-10.0	9.7	Booth 2014
Pipeline	15th	PIP03.1	right mandible	-10.6	9.9	Booth 2014
Pipeline	15th	PIP04.1	atlas	-9.3	9.8	Booth 2014
Rife	15th	Rif-008	femer, right	-10.5	9.2	Morris 2017

Rife	15th	Rif-019	mandible, left	-10.8	9.5	Morris 2017
Cleveland	16th	CLV01.1	mandible	-11.8	9.8	Booth 2014
Cleveland	16th	CLV02.1	skull fragment	-11.5	10.5	Booth 2014
Cleveland	16th	CLV03.1	left tibia	-12.6	9.4	Booth 2014
McKeown	16th	MCK36.1	distal humerus	-14.3	9.2	Booth 2014
Seed-Barker	16th	SEED 01	tibia	-12.0	10.4	Glencross et al. 2022
Seed-Barker	16th	SEED 02	radius	-13.1	9.8	Glencross et al. 2022
Seed-Barker	16th	SEED 03	femur	-12.1	9.9	Glencross et al. 2022
Seed-Barker	16th	SEED 04	radius	-11.5	10.6	Glencross et al. 2022
Seed-Barker	16th	SEED 07	humerus	-12.7	9.9	Glencross et al. 2022
Ball	16th	BALL 01	cranium	-11.6	10.9	Glencross et al. 2022
Ball	16th	BALL 01 dup		-11.8	10.7	Glencross et al. 2022
Ball	16th	BALL 02	mandible	-15.7	10.8	Glencross et al. 2022
Ball	16th	BALL 03	mandible	-13.2	11.2	Glencross et al. 2022
Ball	16th	BALL 04	mandible	-12.0	10.4	Glencross et al. 2022
Ball	16th	BALL 05	mandible	-13.8	11.2	Glencross et al. 2022
Ball	16th	BALL 06	mandible	-18.7	10.5	Glencross et al. 2022
Ball	16th	BALL 07	radius	-13.5	10.4	Glencross et al. 2022
Ball	16th	BALL 08	radius	-17.9	10.0	Glencross et al. 2022
Ball	16th	BALL 08 dup		-18.1	10.1	Glencross et al. 2022
Ball	16th	BALL 09	mandible	-12.5	10.2	Glencross et al. 2022
Ball	16th	BALL 10	mandible	-12.1	10.5	Glencross et al. 2022
Kelly-Campbell	17th	55Ezl 3		-11.5	10.0	Katzenberg 1989
Kelly-Campbell	17th	50Eh16		-11.0	9.6	Katzenberg 1989
Kelly-Campbell	17th	60Em4		-10.6	9.5	Katzenberg 1989
Kelly-Campbell	17th	50Ee54		-12.4	9.6	Katzenberg 1989
Kelly-Campbell	17th	50Eg13		-10.7	9.3	Katzenberg 1989
Kelly-Campbell	17th	Em8		-10.1	9.7	Katzenberg 1989
Kelly-Campbell	17th	55E2		-12.2	9.5	Katzenberg 1989
Kelly-Campbell	17th	Ed69		-10.3	9.7	Katzenberg 1989
Hamilton	17th	Ham-026	mandible, right	-13.2	9.5	Morris 2017
Hamilton	17th	Ham-027	mandible, right	-15.1	9.3	Morris 2017

Hamilton	17th	Ham-028	mandible, right	-13.8	9.7	Morris 2017
Hamilton	17th	Ham-029	mandible, right	-15.8	9.2	Morris 2017
Thorold	17th	Tho-006	mandible	-12.8	8.9	Morris 2017
Thorold	17th	Tho-010	unla, right proximal	-12.7	8.5	Morris 2017
Thorold	17th	Tho-053	calcaneous, left	-13.1	7.9	Morris 2017
Fonger	17th	Fon-061	mandible, left	-12.8	8.4	Morris 2017
Fonger	17th	FON-117	mandible, right	-12.6	8.8	Morris 2017
Fonger	17th	Fon-121	mandible, left	-14.8	9.0	Morris 2017
Walker	17th	Wal-032	right maxilla	-14.0	8.7	Morris 2017
Walker	17th	Wal-057	mandible, left	-12.9	9.4	Morris 2017
Walker	17th	Wal-058	mandible, left	-13.8	9.1	Morris 2017
Walker	17th	Wal-059	mandible, left	-12.2	9.2	Morris 2017
Walker	17th	Wal-060	mandible, left	-13.2	8.8	Morris 2017
Ossossané	17th	OSSO 01	mandible	-11.0	10.6	Glencross et al. 2022
Ossossané	17th	OSSO 02	mandible	-9.9	9.7	Glencross et al. 2022
Ossossané	17th	OSSO 03	mandible	-12.6	11.5	Glencross et al. 2022
Ossossané	17th	OSSO 04	radius	-12.1	9.8	Glencross et al. 2022
Ossossané	17th	OSSO 05	mandible	-10.7	10.3	Glencross et al. 2022
Ossossané	17th	OSSO 06	mandible	-10.7	10.2	Glencross et al. 2022
Ossossané	17th	OSSO 07	mandible	-9.1	9.5	Glencross et al. 2022
Ossossané	17th	OSSO 08	mandible	-10.0	9.9	Glencross et al. 2022
Ossossané	17th	OSSO 09	radius	-11.0	9.5	Glencross et al. 2022
Ossossané	17th	OSSO 10	mandible	-12.5	11.1	Glencross et al. 2022
Ossossané	17th	OSSO 11	radius	-10.5	11.1	Glencross et al. 2022
Mantle	17th	MANT 01	radius	-14.4	10.4	Glencross et al. 2022
Mantle	17th	MANT 02	ulna	-10.9	9.8	Glencross et al. 2022
Mantle	17th	MANT 03	ulna	-11.1	9.5	Glencross et al. 2022
Mantle	17th	MANT 05	ulna	-11.1	9.0	Glencross et al. 2022
Mantle	17th	MANT 06	tibia	-10.2	10.0	Glencross et al. 2022
Mantle	17th	MANT 09	mandible	-12.7	9.9	Glencross et al. 2022
Mantle	17th	MANT 10	mandible	-11.8	10.9	Glencross et al. 2022

S2 Table 2. Human collagen and feces estimate isotope ratios used in Bayesian dietary mixing models.

			Bone Collagen			Feces Estimates	
Site Name	Century	ID	δ13C	δ15N	Source	δ13C	δ15N
Moatfield	14th	LM1	-11.60	11.60	van der Merwe et al. 2003	-15.50	9.00
Moatfield	14th	RM3	-12.00	11.70	van der Merwe et al. 2003	-15.90	9.10
Moatfield	14th	RM2	-10.20	11.70	van der Merwe et al. 2003	-14.10	9.10
Moatfield	14th	RM2	-11.60	12.50	van der Merwe et al. 2003	-15.50	9.90
Moatfield	14th	LM1	-10.80	11.90	van der Merwe et al. 2003	-14.70	9.30
Moatfield	14th	RM3	-14.40	11.90	van der Merwe et al. 2003	-18.30	9.30
Moatfield	14th	RM3	-13.20	12.70	van der Merwe et al. 2003	-17.10	10.10
Moatfield	14th	RM2	-12.90	12.50	van der Merwe et al. 2003	-16.80	9.90
Moatfield	14th	LM1	-13.90	12.10	van der Merwe et al. 2003	-17.80	9.50
Moatfield	14th	LM3	-11.00	13.20	van der Merwe et al. 2003	-14.90	10.60
Moatfield	14th	LM3	-13.20	14.00	van der Merwe et al. 2003	-17.10	11.40
Fairty	14th	cranmand0006 (739)	-10.60	10.90	Pfeiffer et al. 2016	-14.50	8.30
Fairty	14th	cranmand0041 (126)	-10.50	11.90	Pfeiffer et al. 2016	-14.40	9.30
Fairty	14th	cranmand0023 (47)	-11.40	12.10	Pfeiffer et al. 2016	-15.30	9.50
Fairty	14th	cranmand0053 (54)	-14.20	10.90	Pfeiffer et al. 2016	-18.10	8.30
Fairty	14th	cranmand0038 (52)	-10.20	11.90	Pfeiffer et al. 2016	-14.10	9.30
Fairty	14th	cranmand0157 (25)	-10.00	11.50	Pfeiffer et al. 2016	-13.90	8.90
Fairty	14th	cranmand0156 (153)	-10.80	11.50	Pfeiffer et al. 2016	-14.70	8.90
Fairty	14th	FAI R 2	-10.10	11.60	Schwarcz et al 1985	-14.00	9.00
Staines	14th	R max M2	-11.90	12.00	Pfeiffer et al. 2014	-15.80	9.40
Staines	14th	R mand M1	-11.90	12.00	Pfeiffer et al. 2014	-15.80	9.40
Staines	14th	R max M1	-12.50	12.80	Pfeiffer et al. 2014	-16.40	10.20
Staines	14th	L mand M1	-14.10	10.60	Pfeiffer et al. 2014	-18.00	8.00
Teston	15th	L max M1	-11.90	11.00	Pfeiffer et al 2014	-15.80	8.40
Teston	15th	R max M2	-12.50	11.80	Pfeiffer et al 2014	-16.40	9.20
Teston	15th	L max M1	-12.00	11.60	Pfeiffer et al 2014	-15.90	9.00
Teston	15th	L max M1	-12.50	10.70	Pfeiffer et al 2014	-16.40	8.10
Teston	15th	L max M1	-11.90	11.30	Pfeiffer et al 2014	-15.80	8.70
Teston	15th	L max M2	-11.80	10.90	Pfeiffer et al 2014	-15.70	8.30

Uxbridge	15th	cranmand0168 (U3L9S8 162)	-13.20	13.80	Pfeiffer et al 2016	-17.10	11.20
Uxbridge	15th	cranmand0085 (U2L9S6 232)	-11.90	14.10	Pfeiffer et al 2016	-15.80	11.50
Uxbridge	15th	cranmand0007 (L20S3 95)	-10.40	15.10	Pfeiffer et al 2016	-14.30	12.50
Uxbridge	15th	cranmand0040 (U3 L10 S7 397)	-12.00	12.10	Pfeiffer et al 2016	-15.90	9.50
Uxbridge	15th	cranmand0069 (U2 L5 S4 A6)	-11.80	11.90	Pfeiffer et al 2016	-15.70	9.30
Uxbridge	15th	cranmand0106 (U2 L8 S4 211)	-10.30	11.60	Pfeiffer et al 2016	-14.20	9.00
Uxbridge	15th	cranmand0022 (East wall 106)	-11.70	15.80	Pfeiffer et al 2016	-15.60	13.20
Uxbridge	15th	UX1	-10.10	10.20	Harrison and Katzenberg 2003	-14.00	7.60
Uxbridge	15th	UX2	-11.30	11.00	Harrison and Katzenberg 2003	-15.20	8.40
Uxbridge	15th	UX3	-11.20	12.00	Harrison and Katzenberg 2003	-15.10	9.40
Uxbridge	15th	UX4	-11.00	10.10	Harrison and Katzenberg 2003	-14.90	7.50
Uxbridge	15th	UX5	-10.20	11.00	Harrison and Katzenberg 2003	-14.10	8.40
Uxbridge	15th	UX6	-10.80	11.80	Harrison and Katzenberg 2003	-14.70	9.20
Uxbridge	15th	UX7	-11.20	10.90	Harrison and Katzenberg 2003	-15.10	8.30
Uxbridge	15th	UX8	-10.30	11.40	Harrison and Katzenberg 2003	-14.20	8.80
Uxbridge	15th	UX9	-11.30	11.60	Harrison and Katzenberg 2003	-15.20	9.00
Hidden Spring	16th	L max M2	-13.00	11.80	Pfeiffer et al. 2014	-16.90	9.20
Milne	16th	cranmand0002 (mm-19)	-11.20	15.20	Pfeiffer et al. 2016	-15.10	12.60
Kleinburg	16th	#2030	-13.00	12.20	Pfeiffer et al. 2016	-16.90	9.60
Kleinburg	16th	#190	-11.00	12.30	Pfeiffer et al. 2016	-14.90	9.70
Kleinburg	16th	18:14 05	-12.30	12.70	Pfeiffer et al. 2016	-16.20	10.10
Kleinburg	16th	#213	-12.50	11.70	Pfeiffer et al. 2016	-16.40	9.10
Kleinburg	16th	#2073	-10.10	12.30	Pfeiffer et al. 2016	-14.00	9.70
Kleinburg	16th	#31	-12.20	11.70	Pfeiffer et al. 2016	-16.10	9.10
Kleinburg	16th	#2130	-12.00	11.90	Pfeiffer et al. 2016	-15.90	9.30
Kleinburg	16th		-11.20	12.70	Pfeiffer et al. 2016	-15.10	10.10
Kleinburg	16th		-12.60	11.70	Pfeiffer et al. 2016	-16.50	9.10
Kleinburg	16th	KLE R2	-11.70	12.00	Schwarcz et al 1985	-15.60	9.40
Kleinburg	16th	KLE R3	-12.20	12.30	Schwarcz et al 1985	-16.10	9.70
Kleinburg	16th	KLE R4	-12.20	12.40	Schwarcz et al 1985	-16.10	9.80
Ball	16th	R1	-12.70	11.70	Schwarcz et al 1985	-16.60	9.10
Ball	16th	R2	-13.70	12.20	Schwarcz et al 1985	-17.60	9.60

Ball	16th	R3	-11.20	10.80	Schwarcz et al 1985	-15.10	8.20
Ball	16th	R4	-12.10	11.00	Schwarcz et al 1985	-16.00	8.40
Ball	16th	R5	-13.20	12.20	Schwarcz et al 1985	-17.10	9.60
Ball	16th	R6	-9.70	14.60	Schwarcz et al 1985	-13.60	12.00
Maurice	17th	cranmand0004 (W8 N4 1292)	-12.20	14.10	Pfeiffer et al. 2016	-16.10	11.50
Maurice	17th	cranmand0049 (W10 N4 1121)	-13.20	13.30	Pfeiffer et al. 2016	-17.10	10.70
Warminster	17th	cranmand0001 (91)	-11.50	12.00	Pfeiffer et al. 2016	-15.40	9.40
Warminster	17th	cranmand0021 (114)	-12.60	12.30	Pfeiffer et al. 2016	-16.50	9.70
Warminster	17th	cranmand0013 (115)	-11.50	11.30	Pfeiffer et al. 2016	-15.40	8.70
Christian Is	17th	maxillary, no provenience/M_2	-10.80	11.40	Pfeiffer et al. 2016	-14.70	8.80
Kelley Campbell	17th	OSS1	-11.40	12.00	Schwarcz et al. 1985	-15.30	9.40
Kelley Campbell	17th	OSS2	-11.30	12.20	Schwarcz et al. 1985	-15.20	9.60
Kelley Campbell	17th	OSS8	-12.50	14.10	Schwarcz et al. 1985	-16.40	11.50
Kelley Campbell	17th	OSS11	-13.00	13.10	Schwarcz et al. 1985	-16.90	10.50
Kelley Campbell	17th	OSS16	-12.60	13.20	Schwarcz et al. 1985	-16.50	10.60
Ossossané Ossuary	17th	OS2	-12.10	12.40	Schwarcz et al. 1985	-16.00	9.80
Ossossané Ossuary	17th	OS3	-11.10	13.90	Schwarcz et al. 1985	-15.00	11.30
Ossossané Ossuary	17th	OS4	-11.50	10.80	Schwarcz et al. 1985	-15.40	8.20
Ossossané Ossuary	17th	OS5	-11.80	12.40	Schwarcz et al. 1985	-15.70	9.80
Ossossané Ossuary	17th	OS6	-11.00	11.00	Schwarcz et al. 1985	-14.90	8.40

S2 Table 3. Terrestrial prey collagen isotope ratios used in Bayesian dietary mixing models.

Sample	Species	$\delta^{15}N$	$\delta^{13}C$	Reference
DOR04.1	bear	5.58	-24.03	Booth (2014)
DOR05.1	bear	6.38	-22.83	Booth (2014)
DOR06.1	bear	7.61	-22.64	Booth (2014)
DOR07.1	bear	5.34	-25.02	Booth (2014)
DOR08.1	bear	6.68	-23.96	Booth (2014)
DOR09.1	bear	7.38	-23.09	Booth (2014)
DOR10.1	bear	6.64	-23.62	Booth (2014)
HOL02.1	bear	5.63	-22.8	Booth (2014)
HOL16.1	bear	3.77	-21.25	Booth (2014)

WIA02.1	bear	5.68	-20.44	Booth (2014)
CLV04.1	bear	7.88	-20.58	Booth (2014)
CLV06.1	bear	4.99	-22.44	Booth (2014)
MCK01.1	bear	4.75	-20.02	Booth (2014)
MCK07.1	bear	4.89	-22.6	Booth (2014)
MCK15.1	bear	3.43	-20.29	Booth (2014)
MCK16.12	bear	4.66	-19.87	Booth (2014)
MCK25.1	bear	5.37	-20.54	Booth (2014)
MCK27.1	bear	5.17	-20.43	Booth (2014)
MCK28.1	bear	5.45	-21.76	Booth (2014)
MCK34.1	bear	3.43	-20.61	Booth (2014)
MCK38.1	bear	5.08	-19.03	Booth (2014)
MCK42.1	bear	3.77	-20.58	Booth (2014)
MCK43.1	bear	3.96	-20.23	Booth (2014)
MCK44.1	bear	4.74	-19.85	Booth (2014)
MCK45.1	bear	4.5	-20.71	Booth (2014)
CAR02.1	bear	5.93	-20.3	Booth (2014)
CAR19.1	bear	5.77	-21.18	Booth (2014)
CAR33.1	bear	4.61	-22.14	Booth (2014)
CAR35.1	bear	4.63	-20.98	Booth (2014)
CAR36.1	bear	5.28	-18.54	Booth (2014)
IWP08.1	bear	4.35	-21.12	Booth (2014)
Bog-033	bear	5.97	-20	Booth (2014)
Bog-043	bear	4.75	-20.46	Booth (2014)
Fon-067	bear	4.98	-20.89	Booth (2014)
Fon-072	bear	5.78	-22.32	Booth (2014)
Ham-024	bear	4.9	-19.92	Booth (2014)
IWP(01)-052	bear	5.71	-22.2	Booth (2014)
IWP(09)-058	bear	4.35	-21.12	Booth (2014)
Lig-012	bear	4.47	-19.98	Booth (2014)
Pri-018	bear	4.92	-20.86	Booth (2014)
Sil-018	bear	6.24	-21.1	Booth (2014)

Sil-020	bear	6.21	-20.71	Booth (2014)
Van-039	bear	5.62	-20.15	Booth (2014)
Van-067	bear	6.59	-22.9	Booth (2014)
Van-071	bear	6.19	-21.45	Booth (2014)
Van-097	bear	5.98	-21.79	Booth (2014)
Van-114	bear	4.69	-21.38	Booth (2014)
Wal-045	bear	4.76	-20.23	Booth (2014)
Wal-046	bear	5.87	-21.85	Booth (2014)
Wal-047	bear	5.89	-20.48	Booth (2014)
Bog-038	beaver	4.79	-21.37	Booth (2014)
Wal-040	beaver	1.4	-19.48	Booth (2014)
Wal-041	beaver	6.12	-22.34	Booth (2014)
DOR16.1	deer	5.44	-23.52	Booth (2014)
DOR17.1	deer	6.06	-23	Booth (2014)
DOR18.1	deer	4.9	-24.76	Booth (2014)
DOR19.1	deer	5.86	-25.31	Booth (2014)
HOL03.1	deer	6.12	-23.13	Booth (2014)
HOL15.1	deer	4.62	-19.4	Booth (2014)
HOL23.1	deer	6.35	-22.03	Booth (2014)
HOL24.1	deer	5.46	-22.31	Booth (2014)
MCK03.1	deer	6.63	-22.81	Booth (2014)
MCK04.1	deer	4.83	-22.01	Booth (2014)
MCK12.1	deer	5.08	-22.53	Booth (2014)
MCK13.1	deer	4.82	-21.78	Booth (2014)
MCK17.1	deer	4.77	-21.95	Booth (2014)
MCK19.1	deer	6.21	-21.31	Booth (2014)
MCK20.1	deer	3.92	-22.19	Booth (2014)
MCK21.1	deer	3.74	-22.77	Booth (2014)
MCK22.1	deer	4.3	-24.28	Booth (2014)
MCK23.1	deer	5.11	-22.43	Booth (2014)
MCK33.1	deer	5.75	-23.22	Booth (2014)
MCK46.1	deer	6.49	-21.91	Booth (2014)

BrB-010	deer	4.75	-23.46	Morris (2015)
BrB-011	deer	4.88	-23.63	Morris (2015)
BrB-012	deer	4.35	-21.6	Morris (2015)
BrB-013	deer	4.99	-23.6	Morris (2015)
Bog-054	deer	5.03	-23.02	Morris (2015)
Clv-015	deer	6.12	-22.05	Morris (2015)
Clv-016	deer	5.68	-22.38	Morris (2015)
Clv-017	deer	8.16	-21.19	Morris (2015)
Clv-019	deer	5.13	-22.65	Morris (2015)
Cra-001	deer	4.58	-23.33	Morris (2015)
Crf-002	deer	4.15	-23.45	Morris (2015)
Crf-095	deer	5.17	-21.85	Morris (2015)
Dav-001	deer	3.9	-23.92	Morris (2015)
Dav-003	deer	4.27	-23.16	Morris (2015)
Dav-004	deer	6.16	-22.01	Morris (2015)
Fon-001	deer	4.98	-22.8	Morris (2015)
Fon-009	deer	6.14	-22.79	Morris (2015)
Fon-014	deer	5.81	-22.19	Morris (2015)
Fon-019	deer	5.46	-22.79	Morris (2015)
Fon-019 mDUP	deer	5.61	-22.64	Morris (2015)
Fon-047	deer	5.12	-22.91	Morris (2015)
Fon-047 DUP	deer	2.83	-24.88	Morris (2015)
Ham-004	deer	4.98	-22.02	Morris (2015)
IWP(01)-001	deer	5.29	-23.34	Morris (2015)
IWP(01)-001 mDUP	deer	5.31	-23.38	Morris (2015)
IWP(01)-009	deer	5.68	-23.36	Morris (2015)
IWP(01)-025	deer	5.65	-24.07	Morris (2015)
IWP(01)-025 DUP	deer	5.34	-23.85	Morris (2015)
IWP(01)-036 DUP	deer	5.47	-23.64	Morris (2015)
IWP(01)-036 mDUP	deer	4.96	-23.68	Morris (2015)
IWP(03)-23	deer	4.85	-23.49	Morris (2015)
IWP(09)-002	deer	4.92	-23.84	Morris (2015)

IWP(09)-047	deer	4.78	-22.95	Morris (2015)
IWP(09)-047 DUP	deer	4.8	-23.32	Morris (2015)
IWP(09)-047 DUP	deer	4.91	-22.79	Morris (2015)
IWP(09)-054	deer	5.07	-23.31	Morris (2015)
IWP(09)-054 mDUP	deer	5.25	-23.54	Morris (2015)
IWP(09)-134	deer	5.19	-23.43	Morris (2015)
IWP(09)-134 mDUP	deer	5.13	-23.4	Morris (2015)
IWP(12)-003	deer	4.7	-22.54	Morris (2015)
IWP(12)-004	deer	5.35	-22.14	Morris (2015)
IWP(12)-005	deer	5.14	-22.94	Morris (2015)
Lia-006	deer	6.82	-23.82	Morris (2015)
Lia-010	deer	8.62	-20.72	Morris (2015)
Mon-004	deer	5.96	-22.48	Morris (2015)
Mon-005	deer	4.4	-21.79	Morris (2015)
Mon-006	deer	4.78	-22.66	Morris (2015)
Mon-007	deer	5.33	-23.05	Morris (2015)
Mon-008	deer	5.41	-23.1	Morris (2015)
OLG-001	deer	7	-22.12	Morris (2015)
OLG-002	deer	6.07	-21.7	Morris (2015)
OLG-013	deer	5.08	-23.22	Morris (2015)
Pip(1)-103	deer	3.73	-22.06	Morris (2015)
Pip(1)-157	deer	4.54	-22.43	Morris (2015)
Por-009	deer	6.12	-22.09	Morris (2015)
Por-017	deer	5.01	-22.13	Morris (2015)
Por-017 mDUP	deer	5.04	-21.99	Morris (2015)
Pri-008	deer	4.95	-23.05	Morris (2015)
Pri-017	deer	4.91	-22.35	Morris (2015)
Pri-017 DUP	deer	5.85	-20.24	Morris (2015)
Pri-019	deer	4.41	-22.54	Morris (2015)
Pri-019 DUP	deer	2.99	-22.46	Morris (2015)
Rif-007	deer	8.23	-21.25	Morris (2015)
Rif-007 mDUP	deer	8.05	-21.32	Morris (2015)

Rif-077	deer	6.8	-22.65	Morris (2015)
Sil-019	deer	5.54	-22.65	Morris (2015)
Sil-019 DUP	deer	5.48	-23.25	Morris (2015)
Sil-026	deer	6.09	-23.81	Morris (2015)
Sil-026 DUP	deer	6.17	-23.95	Morris (2015)
Sla-017	deer	5.82	-24.18	Morris (2015)
Tho-002	deer	5.73	-22.07	Morris (2015)
Tho-012	deer	5.75	-22.08	Morris (2015)
Tho-012 mDUP	deer	5.87	-22	Morris (2015)
Tho-018	deer	5.69	-22.07	Morris (2015)
Van-001	deer	5.52	-23	Morris (2015)
Van-001 mDUP	deer	5.82	-23.03	Morris (2015)
Van-003	deer	4.49	-21.82	Morris (2015)
Van003 mDUP	deer	4.47	-21.82	Morris (2015)
Van-018	deer	6.33	-21.45	Morris (2015)
Van-019	deer	5.56	-22.2	Morris (2015)
Van-020	deer	5.35	-23.84	Morris (2015)
Van-022	deer	4.92	-24.06	Morris (2015)
Van-108	deer	5.45	-23.1	Morris (2015)
Wal-003	deer	4.18	-23.79	Morris (2015)
Wal-005	deer	4.29	-23.29	Morris (2015)
Wal-008	deer	6.11	-23.71	Morris (2015)
Wal-009	deer	5.86	-24.32	Morris (2015)
Wal-010	deer	5.54	-23.65	Morris (2015)
Wal-011	deer	4.77	-22.56	Morris (2015)
Wal-013	deer	4.49	-23.54	Morris (2015)
Wal-014	deer	5.61	-24.06	Morris (2015)
Wal-014	deer	4.26	-23.56	Morris (2015)
Wal-016	deer	4.26	-23.84	Morris (2015)
Wal-018	deer	5.08	-22.37	Morris (2015)
Wal-021	deer	5.35	-24.66	Morris (2015)
Wal-036	deer	6.13	-22.4	Morris (2015)

Wal-037	deer	4.76	-23.36	Morris (2015)
Wal-038	deer	5.54	-21.91	Morris (2015)
Win-157	deer	8.17	-21.19	Morris (2015)
Win-159	deer	7.29	-21.93	Morris (2015)
BrB-02	deer	5.5	-20.89	Morris (2015)
BrB-03	deer	5.28	-20.68	Morris (2015)
Clv-033 +	deer	6.25	-20.77	Morris (2015)
Crf-043~	deer	6.04	-20.61	Morris (2015)
Crf-044~	deer	5.77	-20.16	Morris (2015)
Crf-045~	deer	6.74	-17.75	Morris (2015)
Crf-046~	deer	6.6	-18.53	Morris (2015)
Crf-047~	deer	7.24	-17.44	Morris (2015)
Crf-048~	deer	6.56	-18.78	Morris (2015)
Crf-051~	deer	6.17	-20.92	Morris (2015)
Fon-020	deer	6.31	-21.59	Morris (2015)
Fon-033	deer	6.9	-21.01	Morris (2015)
Fon-104	deer	5.39	-21.29	Morris (2015)
Ham-05	deer	8.17	-9.93	Morris (2015)
Ham-06	deer	6.25	-19.8	Morris (2015)
Ham-07	deer	6.13	-20.64	Morris (2015)
Ham-08	deer	6.75	-17.08	Morris (2015)
Ham-09~	deer	6.39	-19.59	Morris (2015)
Ham-10~	deer	4.88	-22.83	Morris (2015)
Ham-11~	deer	5.55	-19.13	Morris (2015)
IWP(01)-30	deer	6.75	-22.37	Morris (2015)
IWP(01)-30 DUP	deer	6.76	-22.31	Morris (2015)
IWP(03)-02	deer	6.76	-21.88	Morris (2015)
IWP(03)-06	deer	6.58	-21.45	Morris (2015)
IWP(03)-07	deer	5.79	-23.48	Morris (2015)
IWP(03)-08	deer	5.47	-23.18	Morris (2015)
IWP(03)-15	deer	6.14	-21.85	Morris (2015)
IWP(09)-009	deer	5.67	-21.49	Morris (2015)

IWP(09)-012	deer	5.75	-23.56	Morris (2015)
IWP(09)-012 DUP	deer	6.6	-23.28	Morris (2015)
IWP(09)-032	deer	6.78	-22.07	Morris (2015)
IWP(09)-048	deer	4.72	-20.21	Morris (2015)
IWP(09)-079	deer	6.67	-23.24	Morris (2015)
IWP(09)-083	deer	5.96	-23.67	Morris (2015)
IWP(09)-083 mDUP	deer	6.29	-23.75	Morris (2015)
IWP(09)-088	deer	8.49	-22.45	Morris (2015)
IWP(09)-119	deer	5.71	-23.17	Morris (2015)
IWP(09)-122	deer	7.64	-21.3	Morris (2015)
Pip(1)-010^	deer	6.18	-20.52	Morris (2015)
Pip(1)-023 +	deer	6.39	-20.76	Morris (2015)
Pip(1)-024 +	deer	6.03	-21.05	Morris (2015)
Pip(1)-024 mDUP	deer	6.15	-20.83	Morris (2015)
Pip(1)-024 mDUP DUP	deer	5.21	-20.77	Morris (2015)
Pip(1)-025	deer	6.03	-21.52	Morris (2015)
Pip(1)-048	deer	6.84	-19.88	Morris (2015)
Pip(1)-075	deer	8.49	-20.62	Morris (2015)
Pip(1)-179	deer	5.97	-20.4	Morris (2015)
Pip(1)-184	deer	5.44	-22.5	Morris (2015)
Pip(2)-070	deer	6.86	-20.07	Morris (2015)
Pri-007	deer	5.24	-18.33	Morris (2015)
Rif-062	deer	7.12	-20.63	Morris (2015)
Rif-080	deer	6.81	-22.34	Morris (2015)
Rif-092	deer	6.78	-19.8	Morris (2015)
Rif-092 mDUP	deer	6.72	-19.74	Morris (2015)
Rif-107	deer	5.18	-22.96	Morris (2015)
Rif-107 DUP	deer	5.05	-23.07	Morris (2015)
Tho-035	deer	5.03	-22.36	Morris (2015)
Tho-046	deer	5.86	-22.31	Morris (2015)
Tho-054	deer	4	-21.78	Morris (2015)
Tho-054 DUP	deer	4.81	-21.8	Morris (2015)

Tho-058	deer	4.87	-22.22	Morris (2015)
Tho-065	deer	6.6	-22.16	Morris (2015)
Van-011	deer	5.56	-21.33	Morris (2015)
Van-012	deer	6.76	-21.32	Morris (2015)
Van-017	deer	6.31	-20.93	Morris (2015)
Wal-050	deer	5.46	-20.19	Morris (2015)
Wal-050 mDUP	deer	5.61	-20.2	Morris (2015)
Wal-051	deer	5.63	-21.95	Morris (2015)
Win-047	deer	6.92	-19.55	Morris (2015)
Win-047 DUP	deer	6.85	-19.58	Morris (2015)
Win-221	deer	6.11	-19.02	Morris (2015)
Crf-077 DUP	fox	10.4	-19.62	Morris (2015)
Crf-077	fox	10.3	-19.67	Morris (2015)
Pip(2)-016	fox	8.51	-18.59	Morris (2015)
Tho-011	fox	7.57	-17.95	Morris (2015)
Van-070	fox	8.9	-19.53	Morris (2015)
Win-154	fox	9.08	-18.37	Morris (2015)
Cra-015	groundhog	4.25	-23.3	Morris (2015)
Fon-025	groundhog	2.53	-24.22	Morris (2015)
Fon-049	groundhog	3.94	-19.4	Morris (2015)
Lig-004	groundhog	2.27	-23.21	Morris (2015)
Lig-009	groundhog	2.79	-23.05	Morris (2015)
Lig-014	groundhog	2.67	-23.3	Morris (2015)
Tho-007	groundhog	2.27	-23.76	Morris (2015)
Van-044	groundhog	3.09	-25.6	Morris (2015)
Van-056	groundhog	3.11	-23.66	Morris (2015)
Van-069	groundhog	3.05	-25.49	Morris (2015)
Van-072	groundhog	3.13	-26.39	Morris (2015)
Van-080	groundhog	2.73	-25.75	Morris (2015)
Van-093	groundhog	3.2	-25.67	Morris (2015)
Van-095	groundhog	2.95	-26.45	Morris (2015)
Van-113	groundhog	4.86	-22.89	Morris (2015)

Van-119	groundhog	3.01	-25.76	Morris (2015)
Wal-017	groundhog	2.13	-23.17	Morris (2015)
Wal-020	groundhog	3.34	-25.05	Morris (2015)
Lia-014	muskrat	4.71	-20.43	Morris (2015)
Wal-025	muskrat	7.28	-23	Morris (2015)
Wal-052	muskrat	6.79	-20.55	Morris (2015)
Lia-007	porcupine	4.93	-19.86	Morris (2015)
Van-102	porcupine	5.6	-21.41	Morris (2015)
Wal-054	porcupine	4.42	-20.21	Morris (2015)
OLG-015	rabbit	4.66	-19.74	Morris (2015)
Pip(2)-017	rabbit	4.11	-19.49	Morris (2015)
Tho-019	rabbit	3.44	-22.12	Morris (2015)
Tho-023	rabbit	3.46	-22.08	Morris (2015)
Van-068	rabbit	3.96	-27.35	Morris (2015)
Van-118	rabbit	4.14	-23.1	Morris (2015)
Wal-024	rabbit	4.08	-22.05	Morris (2015)
Wal-053	rabbit	2.09	-27.08	Morris (2015)
PRY01.1	raccoon	9.36	-21.34	Booth (2014)
PRY02.1	raccoon	10.37	-20.91	Booth (2014)
PRY03.1	raccoon	8.87	-21.08	Booth (2014)
PRY07.1	raccoon	9.35	-21.82	Booth (2014)
PRY08.1	raccoon	9.65	-23.13	Booth (2014)
PRY09.1	raccoon	9.45	-21.16	Booth (2014)
PRY11.1	raccoon	8.8	-20.56	Booth (2014)
PRY12.1	raccoon	9.16	-20.39	Booth (2014)
PRY13.1	raccoon	8.72	-20.45	Booth (2014)
PRY15.1	raccoon	9.18	-20.69	Booth (2014)
PRY16.1	raccoon	9.83	-21.45	Booth (2014)
PRY17.1	raccoon	6.18	-20.93	Booth (2014)
PRY18.1	raccoon	8.81	-21.23	Morris (2015)
Bog-002	raccoon	8.09	-20.96	Morris (2015)
Crf-039	raccoon	6.76	-13.98	Morris (2015)

Crf-040	raccoon	7.51	-15.52	Morris (2015)
Fon-109	raccoon	8.95	-20.96	Morris (2015)
IWP(01)-017	raccoon	9.47	-20.36	Morris (2015)
IWP(09)-001	raccoon	7.84	-19.88	Morris (2015)
IWP(09)-004	raccoon	9.65	-21.28	Morris (2015)
IWP(09)-010	raccoon	9.72	-20.66	Morris (2015)
IWP(09)-014	raccoon	9.03	-20.07	Morris (2015)
IWP(09)-018	raccoon	9.45	-21.56	Morris (2015)
IWP(09)-040	raccoon	10.35	-21.27	Morris (2015)
IWP(09)-078	raccoon	7.68	-20.13	Morris (2015)
IWP(09)-111	raccoon	9.13	-20.82	Morris (2015)
IWP(09)-116	raccoon	5.83	-20.77	Morris (2015)
IWP(09)-118	raccoon	8.49	-20.93	Morris (2015)
IWP(09)-131	raccoon	9.64	-20.48	Morris (2015)
Lia-001	raccoon	9.84	-23.48	Morris (2015)
Lia-012	raccoon	4.62	-21.36	Morris (2015)
Lia-013	raccoon	9.11	-23.61	Morris (2015)
Lia-013 DUP	raccoon	9.09	-23.72	Morris (2015)
Mon-001	raccoon	9.25	-21.29	Morris (2015)
Pip(1)-151	raccoon	9.58	-22.87	Morris (2015)
Sil-006	raccoon	9.08	-20.46	Morris (2015)
Van-103	raccoon	9.59	-20.5	Morris (2015)
Van-106	raccoon	8.87	-20.94	Morris (2015)
Wal-042	raccoon	7.44	-21.27	Morris (2015)
Wal-055	raccoon	8.3	-22.25	Morris (2015)
Wal-056	raccoon	8.81	-21.27	Morris (2015)
Win-218	raccoon	7.46	-19.73	Morris (2015)
Win-218 DUP	raccoon	7.44	-19.73	Morris (2015)
Win-233	raccoon	9.56	-21.67	Morris (2015)
Sla-007	raccoon	5.04	-23.18	Morris (2015)
Sla-034	raccoon	5.08	-22.96	Morris (2015)
Wal-026	raccoon	7.23	-25.05	Morris (2015)

Fon-030	squirrel	5.54	-18.53	Morris (2015)
Fon-064	squirrel	4.64	-20.45	Morris (2015)
Fon-091	squirrel	5.04	-19.83	Morris (2015)
Fon-113	squirrel	5.15	-20.02	Morris (2015)
Sla-032	squirrel	4.34	-19.38	Morris (2015)
Tho-005	squirrel	5.03	-19.2	Morris (2015)
Van-041	squirrel	4.52	-20.42	Morris (2015)
Van-042	squirrel	4.09	-20.28	Morris (2015)
Van-052	squirrel	6.66	-18.5	Morris (2015)
Van-085	squirrel	4.97	-19.49	Morris (2015)
Van-090	squirrel	6.39	-19.76	Morris (2015)
Van-091	squirrel	4.86	-19.6	Morris (2015)
Wal-048	squirrel	4.54	-19.32	Morris (2015)
Wal-049	squirrel	3.84	-19.47	Morris (2015)
BrB-02	turkey	5.5	-20.89	Morris et al (2016)
BrB-03	turkey	5.28	-20.68	Morris et al (2016)
Clv-033 +	turkey	6.25	-20.77	Morris et al (2016)
Crf-043~	turkey	6.04	-20.61	Morris et al (2016)
Crf-044~	turkey	5.77	-20.16	Morris et al (2016)
Crf-045~	turkey	6.74	-17.75	Morris et al (2016)
Crf-046~	turkey	6.6	-18.53	Morris et al (2016)
Crf-047~	turkey	7.24	-17.44	Morris et al (2016)
Crf-048~	turkey	6.56	-18.78	Morris et al (2016)
Crf-051~	turkey	6.17	-20.92	Morris et al (2016)
Fon-020	turkey	6.31	-21.59	Morris et al (2016)
Fon-033	turkey	6.9	-21.01	Morris et al (2016)
Fon-104	turkey	5.39	-21.29	Morris et al (2016)
Ham-05	turkey	8.17	-9.93	Morris et al (2016)
Ham-06	turkey	6.25	-19.8	Morris et al (2016)
Ham-07	turkey	6.13	-20.64	Morris et al (2016)
Ham-08	turkey	6.75	-17.08	Morris et al (2016)
Ham-09~	turkey	6.39	-19.59	Morris et al (2016)

Ham-10~	turkey	4.88	-22.83	Morris et al (2016)
Ham-11~	turkey	5.55	-19.13	Morris et al (2016)
IWP(01)-30	turkey	6.75	-22.37	Morris et al (2016)
IWP(03)-02	turkey	6.76	-21.88	Morris et al (2016)
IWP(03)-06	turkey	6.58	-21.45	Morris et al (2016)
IWP(03)-07	turkey	5.79	-23.48	Morris et al (2016)
IWP(03)-08	turkey	5.47	-23.18	Morris et al (2016)
IWP(03)-15	turkey	6.14	-21.85	Morris et al (2016)
IWP(09)-009	turkey	5.67	-21.49	Morris et al (2016)
IWP(09)-012	turkey	5.75	-23.56	Morris et al (2016)
IWP(09)-048	turkey	4.72	-20.21	Morris et al (2016)
IWP(09)-079	turkey	6.67	-23.24	Morris et al (2016)
IWP(09)-083	turkey	5.96	-23.67	Morris et al (2016)
IWP(09)-032	turkey	6.78	-22.07	Morris et al (2016)
IWP(09)-088	turkey	8.49	-22.45	Morris et al (2016)
IWP(09)-119	turkey	5.71	-23.17	Morris et al (2016)
IWP(09)-122	turkey	7.64	-21.3	Morris et al (2016)
Pip(1)-010^	turkey	6.18	-20.52	Morris et al (2016)
Pip(1)-023 +	turkey	6.39	-20.76	Morris et al (2016)
Pip(1)-024 +	turkey	6.03	-21.05	Morris et al (2016)
Pip(1)-025	turkey	6.03	-21.52	Morris et al (2016)
Pip(1)-048	turkey	6.84	-19.88	Morris et al (2016)
Pip(1)-075	turkey	8.49	-20.62	Morris et al (2016)
Pip(1)-179	turkey	5.97	-20.4	Morris et al (2016)
Pip(1)-184	turkey	5.44	-22.5	Morris et al (2016)
Pip(2)-070	turkey	6.86	-20.07	Morris et al (2016)
Pri-007	turkey	5.24	-18.33	Morris et al (2016)
Rif-062	turkey	7.12	-20.63	Morris et al (2016)
Rif-080	turkey	6.81	-22.34	Morris et al (2016)
Rif-092	turkey	6.78	-19.8	Morris et al (2016)
Rif-107	turkey	5.18	-22.96	Morris et al (2016)
Tho-035	turkey	0.03	-22.36	Morris et al (2016)

Tho-046	turkey	5.86	-22.31	Morris et al (2016)
Tho-054	turkey	4	-21.78	Morris et al (2016)
Tho-058	turkey	4.87	-22.22	Morris et al (2016)
Tho-065	turkey	6.6	-22.16	Morris et al (2016)
Van-011	turkey	5.56	-21.33	Morris et al (2016)
Van-012	turkey	6.76	-21.32	Morris et al (2016)
Van-017	turkey	6.31	-20.93	Morris et al (2016)
Wal-050	turkey	5.46	-20.19	Morris et al (2016)
Wal-051	turkey	5.63	-21.95	Morris et al (2016)
Win-047	turkey	6.92	-19.55	Morris et al (2016)
Win-221	turkey	6.11	-19.02	Morris et al (2016)

S2 Table 4. Fish collagen isotope ratios used in Bayesian dietary mixing models.

Specimen	Group	$\delta^{15}N$	$\delta^{13}C$	Reference	Category
Anguilla rostrata (American eel)	Catadromous	7.8	-15.2	Pfeiffer et al. (2016)	Medium N Fish
Anguilla rostrata (American eel)	Catadromous	10.2	-23.5	Pfeiffer et al. (2016)	Medium N Fish
Anguilla rostrata (American eel)	Catadromous	8.6	-14.6	Pfeiffer et al. (2016)	Medium N Fish
Anguilla rostrata (American eel)	Catadromous	9.2	-17.2	Pfeiffer et al. (2016)	Medium N Fish
Anguilla rostrata (American eel)	Catadromous	9.8	-25.8	Pfeiffer et al. (2016)	Medium N Fish
Anguilla rostrata (American eel)	Catadromous	6.4	-22.5	Pfeiffer et al. (2016)	Medium N Fish
Anguilla rostrata (American eel)	Catadromous	8.3	-15.9	Pfeiffer et al. (2016)	Medium N Fish
Anguilla rostrata (American eel)	Catadromous	9.2	-17.1	Pfeiffer et al. (2016)	Medium N Fish
Anguilla rostrata (American eel)	Catadromous	10.5	-16.9	Pfeiffer et al. (2016)	Medium N Fish
Anguilla rostrata (American eel)	Catadromous	10.5	-16.9	Pfeiffer et al. (2016)	Medium N Fish
Anguilla rostrata (American eel)	Catadromous	8.1	-16.2	Pfeiffer et al. (2016)	Medium N Fish
Salmonidae (2)	Salmonidae	10.1	-19.4	Pfeiffer et al. (2016)	High N Fish
Salmo salar (Atlantic salmon)	Salmonidae	10.4	-19.7	Pfeiffer et al. (2016)	High N Fish
Salmo salar (Atlantic salmon)	Salmonidae	10.7	-19.2	Pfeiffer et al. (2016)	High N Fish
Salmo salar (Atlantic salmon)	Salmonidae	10.2	-19.6	Pfeiffer et al. (2016)	High N Fish
Salmo salar (Atlantic salmon)	Salmonidae	9.6	-19.1	Pfeiffer et al. (2016)	High N Fish
Salmo salar (Atlantic salmon)	Salmonidae	10.7	-19.6	Pfeiffer et al. (2016)	High N Fish
Salmo salar (Atlantic salmon)	Salmonidae	9.9	-19.3	Pfeiffer et al. (2016)	High N Fish

Salmo salar (Atlantic salmon)	Salmonidae	10.1	-19.3	Pfeiffer et al. (2016)	High N Fish
Salvelinus namaycush (Lake trout)	Salmonidae	9.7	-19.5	Pfeiffer et al. (2016)	High N Fish
Salvelinus namaycush (Lake trout)	Salmonidae	12.1	-20.7	Pfeiffer et al. (2016)	High N Fish
Salvelinus namaycush (Lake trout)	Salmonidae	11	-20.3	Pfeiffer et al. (2016)	High N Fish
Salvelinus namaycush (Lake trout)	Salmonidae	11.8	-21	Pfeiffer et al. (2016)	High N Fish
Coregonus clupeaformis (Lake whitefish)	Salmonidae	8.8	-20.6	Pfeiffer et al. (2016)	High N Fish
Coregonus clupeaformis (Lake whitefish)	Salmonidae	8.3	-21.7	Pfeiffer et al. (2016)	High N Fish
Coregonus clupeaformis (Lake whitefish)	Salmonidae	8.8	-21.3	Pfeiffer et al. (2016)	High N Fish
Coregonus clupeaformis (Lake whitefish)	Salmonidae	7.6	-20.9	Pfeiffer et al. (2016)	High N Fish
9350	Salmonidae	10.06	-19.89	Guiry et al. (2016)	High N Fish
9351	Salmonidae	10.23	-19.67	Guiry et al. (2016)	High N Fish
9352	Salmonidae	10.44	-19.73	Guiry et al. (2016)	High N Fish
9353	Salmonidae	9.97	-20.28	Guiry et al. (2016)	High N Fish
9354	Salmonidae	9.59	-20.65	Guiry et al. (2016)	High N Fish
9354	Salmonidae	9.7	-20.71	Guiry et al. (2016)	High N Fish
9355	Salmonidae	10.42	-20.1	Guiry et al. (2016)	High N Fish
9356	Salmonidae	10.79	-20.28	Guiry et al. (2016)	High N Fish
9357	Salmonidae	11.01	-20.35	Guiry et al. (2016)	High N Fish
9359	Salmonidae	10.18	-20	Guiry et al. (2016)	High N Fish
9361	Salmonidae	10.31	-20.49	Guiry et al. (2016)	High N Fish
9364	Salmonidae	10.49	-19.64	Guiry et al. (2016)	High N Fish
10254	Salmonidae	10.03	-22.12	Guiry et al. (2016)	High N Fish
10255	Salmonidae	9.9	-22.2	Guiry et al. (2016)	High N Fish
10256	Salmonidae	10.28	-22.68	Guiry et al. (2016)	High N Fish
10257	Salmonidae	10.9	-22.56	Guiry et al. (2016)	High N Fish
10258	Salmonidae	13.72	-15.11	Guiry et al. (2016)	High N Fish
10259	Salmonidae	11.77	-15.38	Guiry et al. (2016)	High N Fish
10260	Salmonidae	10.53	-15.16	Guiry et al. (2016)	High N Fish
10261	Salmonidae	10.72	-21.9	Guiry et al. (2016)	High N Fish
10262	Salmonidae	10.33	-22.1	Guiry et al. (2016)	High N Fish
10642	Salmonidae	13.25	-18.85	Guiry et al. (2016)	High N Fish
10643	Salmonidae	13.07	-19.91	Guiry et al. (2016)	High N Fish

10649	Salmonidae	10.28	-19.62	Guiry et al. (2016)	High N Fish
10650	Salmonidae	10.3	-19.61	Guiry et al. (2016)	High N Fish
10651	Salmonidae	10	-20.31	Guiry et al. (2016)	High N Fish
10652	Salmonidae	10.46	-20.73	Guiry et al. (2016)	High N Fish
10653	Salmonidae	10.19	-19.67	Guiry et al. (2016)	High N Fish
10654	Salmonidae	10.12	-20.1	Guiry et al. (2016)	High N Fish
10655	Salmonidae	10.19	-20.32	Guiry et al. (2016)	High N Fish
10656	Salmonidae	10.2	-20.34	Guiry et al. (2016)	High N Fish
10657	Salmonidae	10.15	-19.83	Guiry et al. (2016)	High N Fish
10658	Salmonidae	12.9	-19.44	Guiry et al. (2016)	High N Fish
10659	Salmonidae	14.75	-18.92	Guiry et al. (2016)	High N Fish
10660	Salmonidae	12.91	-19.42	Guiry et al. (2016)	High N Fish
10661	Salmonidae	12.92	-18.73	Guiry et al. (2016)	High N Fish
10664	Salmonidae	10.53	-20.02	Guiry et al. (2016)	High N Fish
10665	Salmonidae	10.18	-20.15	Guiry et al. (2016)	High N Fish
10666	Salmonidae	9.74	-20.22	Guiry et al. (2016)	High N Fish
10667	Salmonidae	10.32	-20.01	Guiry et al. (2016)	High N Fish
10668	Salmonidae	10.39	-19.96	Guiry et al. (2016)	High N Fish
10669	Salmonidae	10.06	-20.05	Guiry et al. (2016)	High N Fish
10670	Salmonidae	10.72	-20.13	Guiry et al. (2016)	High N Fish
10671	Salmonidae	10.83	-19.92	Guiry et al. (2016)	High N Fish
10672	Salmonidae	9.79	-20.84	Guiry et al. (2016)	High N Fish
10673	Salmonidae	10.77	-20.1	Guiry et al. (2016)	High N Fish
10674	Salmonidae	10.76	-19.63	Guiry et al. (2016)	High N Fish
10675	Salmonidae	10.36	-19.84	Guiry et al. (2016)	High N Fish
10676	Salmonidae	10.1	-19.77	Guiry et al. (2016)	High N Fish
10677	Salmonidae	10.12	-20.9	Guiry et al. (2016)	High N Fish
10678	Salmonidae	10.23	-19.63	Guiry et al. (2016)	High N Fish
10679	Salmonidae	10.56	-19.84	Guiry et al. (2016)	High N Fish
10680	Salmonidae	10.55	-19.36	Guiry et al. (2016)	High N Fish
10681	Salmonidae	10.5	-19.83	Guiry et al. (2016)	High N Fish
10682	Salmonidae	10.02	-19.71	Guiry et al. (2016)	High N Fish

10683	Salmonidae	10.36	-20.1	Guiry et al. (2016)	High N Fish
10684	Salmonidae	10.08	-19.95	Guiry et al. (2016)	High N Fish
10685	Salmonidae	10.36	-19.66	Guiry et al. (2016)	High N Fish
10687	Salmonidae	10.23	-19.55	Guiry et al. (2016)	High N Fish
10688	Salmonidae	10.57	-20.57	Guiry et al. (2016)	High N Fish
10689	Salmonidae	10.05	-20.13	Guiry et al. (2016)	High N Fish
11703	Salmonidae	9.94	-15.26	Guiry et al. (2016)	High N Fish
11704	Salmonidae	9.84	-15.87	Guiry et al. (2016)	High N Fish
11705	Salmonidae	9.96	-16.63	Guiry et al. (2016)	High N Fish
11706	Salmonidae	9.92	-16.6	Guiry et al. (2016)	High N Fish
11707	Salmonidae	9.81	-15.96	Guiry et al. (2016)	High N Fish
10538-1	Salmonidae	11.45	-15.7	Guiry et al. (2016)	High N Fish
10538-2	Salmonidae	11.47	-15.53	Guiry et al. (2016)	High N Fish
10539-1	Salmonidae	11.5	-15.75	Guiry et al. (2016)	High N Fish
10539-2	Salmonidae	11.52	-15.72	Guiry et al. (2016)	High N Fish
10540-1	Salmonidae	12.53	-20.06	Guiry et al. (2016)	High N Fish
10540-2	Salmonidae	12.55	-19.93	Guiry et al. (2016)	High N Fish
10541-1	Salmonidae	13.68	-19.55	Guiry et al. (2016)	High N Fish
10541-2	Salmonidae	12.47	-19.98	Guiry et al. (2016)	High N Fish
10542-1	Salmonidae	13.05	-19.82	Guiry et al. (2016)	High N Fish
10542-2	Salmonidae	12.54	-19.7	Guiry et al. (2016)	High N Fish
10642	Salmonidae	13.25	-18.85	Guiry et al. (2016)	High N Fish
10643	Salmonidae	13.07	-19.91	Guiry et al. (2016)	High N Fish
Coregonus clupeaformis (Lake whitefish)	Salmonidae	8.5	-21.1	Pfeiffer et al. (2016)	High N Fish
Amia calva (Bowfin)	Nominally piscivorous	8	-23.4	Pfeiffer et al. (2016)	Medium N Fish
Amia calva (Bowfin)	Nominally piscivorous	7.6	-23.7	Pfeiffer et al. (2016)	Medium N Fish
Ameiurus nebulosus (Brown bullhead)	Nominally piscivorous	7.1	-21.1	Pfeiffer et al. (2016)	Medium N Fish
Ameiurus nebulosus (Brown bullhead)	Nominally piscivorous	7.2	-23	Pfeiffer et al. (2016)	Medium N Fish
Ameiurus nebulosus (Brown bullhead)	Nominally piscivorous	5.8	-18.5	Pfeiffer et al. (2016)	Medium N Fish
Ameiurus nebulosus (Brown bullhead)	Nominally piscivorous	7	-19.9	Pfeiffer et al. (2016)	Medium N Fish
Ameiurus nebulosus (Brown bullhead)	Nominally piscivorous	6.3	-20.8	Pfeiffer et al. (2016)	Medium N Fish
Ameiurus nebulosus (Brown bullhead)	Nominally piscivorous	5.6	-18.1	Pfeiffer et al. (2016)	Medium N Fish

Esox americanus (Grass pickerel)	Piscivorous	7.5	-23.6	Pfeiffer et al. (2016)	Medium N Fish
Esox americanus (Grass pickerel)	Piscivorous	7.8	-22.9	Pfeiffer et al. (2016)	Medium N Fish
Esox lucius (Northern pike)	Piscivorous	8.8	-20.8	Pfeiffer et al. (2016)	Medium N Fish
Esox lucius (Northern pike)	Piscivorous	9.4	-18.2	Pfeiffer et al. (2016)	Medium N Fish
Esox lucius (Northern pike)	Piscivorous	8.6	-20.6	Pfeiffer et al. (2016)	Medium N Fish
Esox lucius (Northern pike)	Piscivorous	9.3	-18.3	Pfeiffer et al. (2016)	Medium N Fish
Esox lucius (Northern pike)	Piscivorous	9.8	-18.7	Pfeiffer et al. (2016)	Medium N Fish
Micropterus dolomieu (Smallmouth bass)	Nominally piscivorous	8.9	-17.3	Pfeiffer et al. (2016)	Medium N Fish
Micropterus dolomieu (Smallmouth bass)	Nominally piscivorous	9.1	-17.6	Pfeiffer et al. (2016)	Medium N Fish
Micropterus salmoides (Largemouth bass)	Nominally piscivorous	8.5	-18.8	Pfeiffer et al. (2016)	Medium N Fish
Micropterus salmoides (Largemouth bass)	Nominally piscivorous	8	-20	Pfeiffer et al. (2016)	Medium N Fish
Micropterus salmoides (Largemouth bass)	Nominally piscivorous	8.4	-19.2	Pfeiffer et al. (2016)	Medium N Fish
Micropterus salmoides (Largemouth bass)	Nominally piscivorous	7.2	-23.1	Pfeiffer et al. (2016)	Medium N Fish
Micropterus salmoides (Largemouth bass)	Nominally piscivorous	7.9	-18.9	Pfeiffer et al. (2016)	Medium N Fish
Perca flavescens (Yellow perch)	Nominally piscivorous	10.2	-19	Pfeiffer et al. (2016)	Medium N Fish
Perca flavescens (Yellow perch)	Nominally piscivorous	10.2	-21.4	Pfeiffer et al. (2016)	Medium N Fish
Perca flavescens (Yellow perch)	Nominally piscivorous	9.9	-17.8	Pfeiffer et al. (2016)	Medium N Fish
Perca flavescens (Yellow perch)	Nominally piscivorous	8.8	-19	Pfeiffer et al. (2016)	Medium N Fish
Perca flavescens (Yellow perch)	Nominally piscivorous	8.6	-24.1	Pfeiffer et al. (2016)	Medium N Fish
Perca flavescens (Yellow perch)	Nominally piscivorous	9.3	-19	Pfeiffer et al. (2016)	Medium N Fish
Perca flavescens (Yellow perch)	Nominally piscivorous	9.2	-17.4	Pfeiffer et al. (2016)	Medium N Fish
Perca flavescens (Yellow perch)	Nominally piscivorous	9.3	-18.5	Pfeiffer et al. (2016)	Medium N Fish
Stizostedion sp. (Walleye or sauger)	Nominally piscivorous	11.7	-17.4	Pfeiffer et al. (2016)	Medium N Fish
Sander vitreus (Walleye)	Nominally piscivorous	8	-16.3	Pfeiffer et al. (2016)	Medium N Fish
Lota lota (Burbot)	Oily liver	12	-21	Pfeiffer et al. (2016)	High N Fish
Lota lota (Burbot)	Oily liver	12.1	-19.4	Pfeiffer et al. (2016)	High N Fish
Lota lota (Burbot)	Oily liver	14	-20.2	Pfeiffer et al. (2016)	High N Fish
Lota lota (Burbot)	Oily liver	11.3	-21.1	Pfeiffer et al. (2016)	High N Fish
Lota lota (Burbot)	Oily liver	10.5	-16.9	Pfeiffer et al. (2016)	High N Fish
Lota lota (Burbot)	Oily liver	12.7	-20.6	Pfeiffer et al. (2016)	High N Fish
Lota lota (Burbot)	Oily liver	10.2	-19.4	Pfeiffer et al. (2016)	High N Fish
Lota lota (Burbot)	Oily liver	12.8	-19.5	Pfeiffer et al. (2016)	High N Fish

Lota lota (Burbot)	Oily liver	12	-19.7	Pfeiffer et al. (2016)	High N Fish
Catostomus sp. (Sucker)	Non-piscivorous	7.1	-18.8	Pfeiffer et al. (2016)	Low N Fish
Catostomus sp. (Sucker)	Non-piscivorous	6.6	-18.4	Pfeiffer et al. (2016)	Low N Fish
Catostomus sp. (Sucker)	Non-piscivorous	3.6	-23.1	Pfeiffer et al. (2016)	Low N Fish
Catostomus catostomus (Longnose sucker)	Non-piscivorous	5.8	-17.5	Pfeiffer et al. (2016)	Low N Fish
Catostomus commersoni (White sucker)	Non-piscivorous	6	-22.2	Pfeiffer et al. (2016)	Low N Fish
Catostomus commersoni (White sucker)	Non-piscivorous	5.4	-18.5	Pfeiffer et al. (2016)	Low N Fish
Catostomus commersoni (White sucker)	Non-piscivorous	5.5	-18.2	Pfeiffer et al. (2016)	Low N Fish
Catostomus commersoni (White sucker)	Non-piscivorous	5.1	-16.7	Pfeiffer et al. (2016)	Low N Fish
Ictalurus cf. punctatus (probable Channel catfish)	Non-piscivorous	8.1	-17.4	Pfeiffer et al. (2016)	Low N Fish
Ictalurus cf. punctatus (probable Channel catfish)	Non-piscivorous	9.1	-17.4	Pfeiffer et al. (2016)	Low N Fish
(Sunfish)	Non-piscivorous	9.1	-18.2	Pfeiffer et al. (2016)	Low N Fish
Ambloplites rupestris (Rock bass)	Non-piscivorous	8	-24.9	Pfeiffer et al. (2016)	Low N Fish
Ambloplites rupestris (Rock bass)	Non-piscivorous	8.3	-20.9	Pfeiffer et al. (2016)	Low N Fish
Ambloplites rupestris (Rock bass)	Non-piscivorous	8.6	-21.6	Pfeiffer et al. (2016)	Low N Fish
Ambloplites rupestris (Rock bass)	Non-piscivorous	7.8	-21.1	Pfeiffer et al. (2016)	Low N Fish
Ambloplites rupestris (Rock bass)	Non-piscivorous	8.7	-22.4	Pfeiffer et al. (2016)	Low N Fish
Lepomis gibbosus (Pumpkinseed)	Non-piscivorous	6.6	-15.8	Pfeiffer et al. (2016)	Low N Fish
Lepomis gibbosus (Pumpkinseed)	Non-piscivorous	7.2	-23.2	Pfeiffer et al. (2016)	Low N Fish
Lepomis gibbosus (Pumpkinseed)	Non-piscivorous	7.1	-17.6	Pfeiffer et al. (2016)	Low N Fish
Lepomis gibbosus (Pumpkinseed)	Non-piscivorous	4.4	-20.3	Pfeiffer et al. (2016)	Low N Fish
Pomoxis cf. nigromaculatus	Non-piscivorous	7.9	-20.1	Pfeiffer et al. (2016)	Low N Fish
Pomoxis cf. nigromaculatus	Non-piscivorous	8	-21.5	Pfeiffer et al. (2016)	Low N Fish
Aplodinotus grunniens (Freshwater drum)	Non-piscivorous	6.1	-11.5	Pfeiffer et al. (2016)	Low N Fish

S2 Table 5. Micromammal collagen isotope ratios used in Bayesian dietary mixing models.

$\delta^{13}C$	$\delta^{15}N$	Reference
-16.6	6.6	Guiry et al. 2021
-7.8	9.9	Guiry et al. 2021
-14.1	10.1	Guiry et al. 2021
-13.6	8.4	Guiry et al. 2021
-8.1	9.7	Guiry et al. 2021
-8.6	9.3	Guiry et al. 2021
-8.7	9.4	Guiry et al. 2021
-8.9	10.2	Guiry et al. 2021
-14.8	13.2	Guiry et al. 2021
-13.1	9.7	Guiry et al. 2021
-9.8	10.9	Guiry et al. 2021
-10.5	11.5	Guiry et al. 2021
-6.5	10	Guiry et al. 2021
-7.1	9.2	Guiry et al. 2021

S2 Table 6. Source isotope means, standard deviations and concentrations used in models.

Sources	Mean $\delta^{13}C$	SD $\delta^{13}C$	Mean $\delta^{15}N$	SD $\delta^{15}N$	Conc C	Conc N	n
Maize	-9.36	1.67	4.76	1.63	0.5	0.02	81
Terrestrial	-21.64	1.94	5.89	1.63	0.56	0.12	404
HighN_Fish	-19.59	1.63	10.82	1.29	0.48	0.1	105
MedN_Fish	-19.49	2.74	8.54	1.32	0.52	0.12	43
LowN_Fish	-19.45	2.97	6.96	1.52	0.51	0.15	23
HighC_micromammals	-10.59	3.23	9.86	1.49	0.56	0.12	14
human feces	varies by site and century see S2 Table 2				0.2	0.03	13

S2 Table 7. Cand N concentration calculations.

Source	Protein	Carb	Fat	N Conc.	C Conc.
deer	0.76	0	0.25	0.12	0.58
salmon`	0.63	0	0.2	0.10	0.48
perch	0.93	0	0.04	0.15	0.51
bass	0.78	0	0.15	0.12	0.52
turkey	0.71	0	0.28	0.11	0.58
squirrel	0.81	0	0.12	0.13	0.51
maize	0.1	0.91	0.05	0.02	0.50
beaver	0.83	0	0.17	0.13	0.56
feces				0.03	0.20
terrestrial average	0.78	0	0.21	0.12	0.56

S2 Table 8. Trophic enrichment factors used in models.

Sources	Mean $\delta^{13}C$	SD $\delta^{13}C$	Mean $\delta^{15}N$	SD $\delta^{15}N$
Maize	5	0.1	3	0.1
Terrestrial	1.1	0.2	3.8	0.2
HighN_Fish	1.1	0.2	3.8	0.2
MedN_Fish	1.1	0.2	3.8	0.2
LowN_Fish	1.1	0.2	3.8	0.2
HighC_micromammals	1.1	0.2	3.8	0.2
human feces	5	0.1	3	0.1

Reference Cited

Booth, Laura. 2014. A stable isotope analysis of faunal remains from special deposits on Ontario Iroquoian tradition sites. PhD. dissertation, The University of Western Ontario, London. https://ir.lib.uwo.ca/etd/2644/

Glencross, Bonnie, Gary Warrick, Taylor Smith, and Tracy L. Prowse. 2022. Estimating ancient Huron-Wendat diet in southern Ontario using stable isotopes from dogs. *Journal of Archaeological Science: Reports* 41:103324. doi:10.1016/j.jasrep.2021.103324

Guiry, Eric, Trevor J. Orchard, Suzanne Needs-Howarth, and Paul Szpak. 2021. Isotopic evidence for garden hunting and resource depression in the Late Woodland of Northeastern North America. *American Antiquity* 86: 90-110. doi:10.1017/aaq.2020.86

Guiry, Eric J., Suzanne Needs-Howarth, Kevin D. Friedland, Alicia L. Hawkins, Paul Szpak, Rebecca Macdonald, Michelle Courtemanche, Erling Holm, and Michael P. Richards. 2016. Lake Ontario salmon (*Salmo salar*) were not migratory: A long-standing historical debate solved through stable isotope analysis. *Scientific Reports* 6:36249. https://doi.org/10.1038/srep36249

Harrison, Roman G., and M. Anne Katzenberg. 2003. Paleodiet studies using stable carbon isotopes from bone apatite and collagen: examples from Southern Ontario and San Nicolas Island, California. *Journal of Anthropological Archaeology* 22: 227-244. doi:10.1016/S0278-4165(03)00037-0

Katzenberg, M. Anne. 1989. Stable isotope analysis of archaeological faunal remains from southern Ontario. *Journal of Archaeological Science* 16: 319-329. doi:10.1016/0305-4403(89)90008-3

Morris, Zoe H. 2015. Reconstructing subsistence practices of southwestern Ontario Late Woodland Peoples (AD 900-1600) using stable isotopic analyses of faunal material. Ph.D. dissertation. The University of Western Ontario. https://ir.lib.uwo.ca/etd/2921/

Morris, Zoe, Christine White, Lisa Hodgetts, and Fred Longstaffe. 2016. Maize provisioning of Ontario Late Woodland turkeys: isotopic evidence of seasonal, cultural, spatial and temporal variation." *Journal of Archaeological Science: Reports* 10: 596-606. doi:10.1016/j.jasrep.2016.06.017

Pfeiffer, Susan, Judith C. Sealy, Ronald F. Williamson, Suzanne Needs-Howarth, and Louis Lesage. 2016. Maize, fish, and deer: investigating dietary staples among ancestral Huron-Wendat villages, as documented from tooth samples. *American Antiquity* 81: 515-532. doi:10.1017/S0002731600003978

Pfeiffer, Susan, Ronald F. Williamson, Judith C. Sealy, David G. Smith, and Meradeth H. Snow. 2014. Stable dietary isotopes and mtDNA from Woodland period southern Ontario people: results from a tooth sampling protocol. *Journal of Archaeological Science* 42: 334-345. doi:10.1016/j.jasrep.2020.102466

Schwarcz, Henry P., Jerry Melbye, M. Anne Katzenberg, and Martin Knyf. 1985. Stable isotopes in human skeletons of southern Ontario: reconstructing palaeodiet. *Journal of Archaeological Science* 12: 187-206. doi:10.1016/0305-4403(85)90020-2

Van der Merwe, Nikolaas J., Ronald F. Williamson, Susan Pfeiffer, Stephen Cox Thomas, and Kim Oakberg Allegretto. 2003. The Moatfield ossuary: isotopic dietary analysis of an Iroquoian community, using dental tissue. *Journal of Anthropological Archaeology* 22:245-261. doi:10.1016/S0278-4165(03)00038-2
